# CBP/p300 Bromodomains Regulate Amyloid-like Protein Aggregation upon Aberrant Lysine Acetylation

**DOI:** 10.1016/j.chembiol.2016.11.009

**Published:** 2017-01-19

**Authors:** Heidi Olzscha, Oleg Fedorov, Benedikt M. Kessler, Stefan Knapp, Nicholas B. La Thangue

**Affiliations:** 1 Department of Oncology, University of Oxford, Old Road Campus Research Building, Old Road Campus, Roosevelt Drive, Oxford OX3 7DQ, UK; 2 Structural Genomics Consortium, University of Oxford, Old Road Campus Research Building, Old Road Campus, Roosevelt Drive, Oxford OX3 7DQ, UK; 3 Nuffield Department of Medicine, Target Discovery Institute, University of Oxford, Roosevelt Drive, Oxford OX3 7FZ, UK

**Keywords:** acetylation, aggregation, amyloid, bromodomain, histone deacetylase, p300, CBP, huntingtin, proteostasis, inhibitor

## Abstract

Lysine acetylation is becoming increasingly recognized as a general biological principle in cellular homeostasis, and is subject to abnormal control in different human pathologies. Here, we describe a global effect on amyloid-like protein aggregation in human cells that results from aberrant lysine acetylation. Bromodomain reader proteins are involved in the aggregation process and, using chemical biology and gene silencing, we establish that p300/CBP bromodomains are necessary for aggregation to occur. Moreover, protein aggregation disturbs proteostasis by impairing the ubiquitin proteasome system (UPS) and protein translation, resulting in decreased cell viability. p300/CBP bromodomain inhibitors impede aggregation, which coincides with enhanced UPS function and increased cell viability. Aggregation of a pathologically relevant form of huntingtin protein is similarly affected by p300/CBP inhibition. Our results have implications for understanding the molecular basis of protein aggregation, and highlight the possibility of treating amyloid-like pathologies and related protein folding diseases with bromodomain inhibitor-based strategies.

## Highlights

• Aberrant acetylation causes amyloid-like aggregation and disturbs proteostasis
• p300/CBP bromodomain proteins are involved in the protein aggregation
• p300/CBP bromodomain inhibitors reverse the cytotoxicity and restore proteostasis
• Bromodomain inhibitors also decrease pathological huntingtin protein aggregation

## Graphical Abstract

**Figure:**
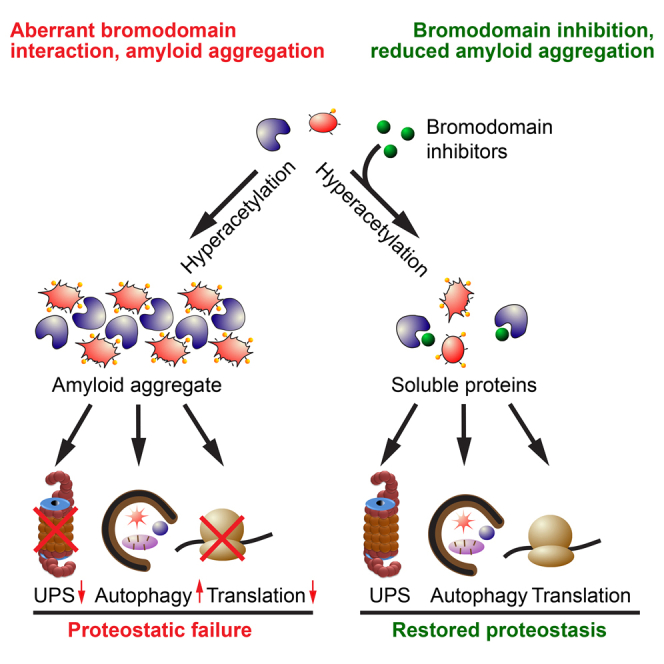

## Teaser

Olzscha et al. demonstrate that aberrantly acetylated proteins form amyloid-like aggregates in human cells. Inhibition of the p300/CBP bromodomains with small-molecule inhibitors reverses the aggregation-induced cytotoxicity. Aggregates caused by a pathologically elongated huntingtin behave in a similar way.

## Introduction

Protein acetylation on lysine side chains is a widespread post-translational modification involved in diverse cellular processes and biological pathways (Choudhary et al., 2014). Histone acetyltransferases (HATs) such as CBP/p300 “write” the acetylation mark, contrasting with histone deacetylases (HDACs) which “erase” the mark (Kouzarides, 1999), while bromodomain-containing proteins including CBP/p300 “read” the mark (Chan and La Thangue, 2001, Filippakopoulos and Knapp, 2014, Zeng and Zhou, 2002). It is generally believed that the equilibrium between modified and unmodified lysine acetylation states has to be tightly regulated to maintain the normal biological role of acetylation in cellular homeostasis. Thus, acetylation is involved in a variety of biological processes, including transcription, protein translation, and degradation as well as cell-cycle control and apoptosis (New et al., 2012). Acetylation can affect the structure of proteins; for example, in the N-terminal region of histone proteins it increases the α-helical content (Wang et al., 2000) and intrinsically unstructured regions (Hansen et al., 2006), perhaps leading to misfolding and aggregation. The cell death caused by aberrant levels of protein acetylation resulting, for example, from HDAC inhibitor (HDI) treatment, is consistent with the biological importance of lysine acetylation (Choudhary et al., 2009, New et al., 2012).

Deregulated acetylation has been suggested to be involved in the pathology of several types of disease, including cancer, inflammation, and metabolic and neurodegenerative diseases (Khan and La Thangue, 2012, Marks et al., 2001, Saha and Pahan, 2006, Zhao et al., 2010). The precise contribution made by acetylation to the disease pathology is, however, much debated. Since lysine acetylation of histones is involved with chromatin control, abnormal gene expression is one level that might be affected (Grunstein, 1997, Marks et al., 2001). Nevertheless, as diverse proteins involved with multiple biological processes are acetylated (Choudhary et al., 2009), acetylation is likely to affect a variety of pathologically relevant mechanisms in addition to chromatin control. One idea that we have considered, given the widespread effect on proteins, is whether lysine acetylation affects protein homeostasis (often referred to as proteostasis), namely the balance between protein synthesis, maturation, and degradation (Balch et al., 2008, Hartl et al., 2011). We have therefore examined whether protein aggregation and, potentially, misfolding, occur in cellular conditions of abnormal lysine acetylation. To reflect the writer-reader interplay established for lysine acetylation (Filippakopoulos and Knapp, 2014), we also addressed whether any influence of lysine acetylation on proteostasis involves bromodomain proteins.

Here, we describe amyloid-like protein aggregates that occur under conditions of aberrant lysine acetylation, dependent on p300/CBP bromodomain proteins for their formation. The presence of amyloid-like aggregates coincides with increased cytotoxicity, and cell viability can be restored upon co-treating cells with small-molecule p300/CBP bromodomain inhibitors (BDIs), which reflect reduced levels of protein aggregates. The p300/CBP bromodomain proteins, together with proteins involved in proteostasis, are present in the aggregates, and p300/CBP proteins are necessary for the aggregates to occur. The presence of amyloid-like aggregates impinges on proteostasis, as both protein degradation and protein translation are affected, which similarly can be relieved upon treating cells with p300/CBP-specific BDIs. Furthermore, the amyloid-like protein aggregates formed by a pathologically relevant polyglutamine-expanded Huntington's disease protein are affected by p300/CBP BDIs. Our results suggest a crucial role for protein acetylation and bromodomains as a global regulator of cellular proteostasis, and highlight a novel therapeutic approach based on bromodomain inhibition for treating pathologies dependent upon protein misfolding and aggregation.

## Results

### Amyloid-like Aggregates in HDAC Inhibitor-Treated Cells

**Figure 1:**
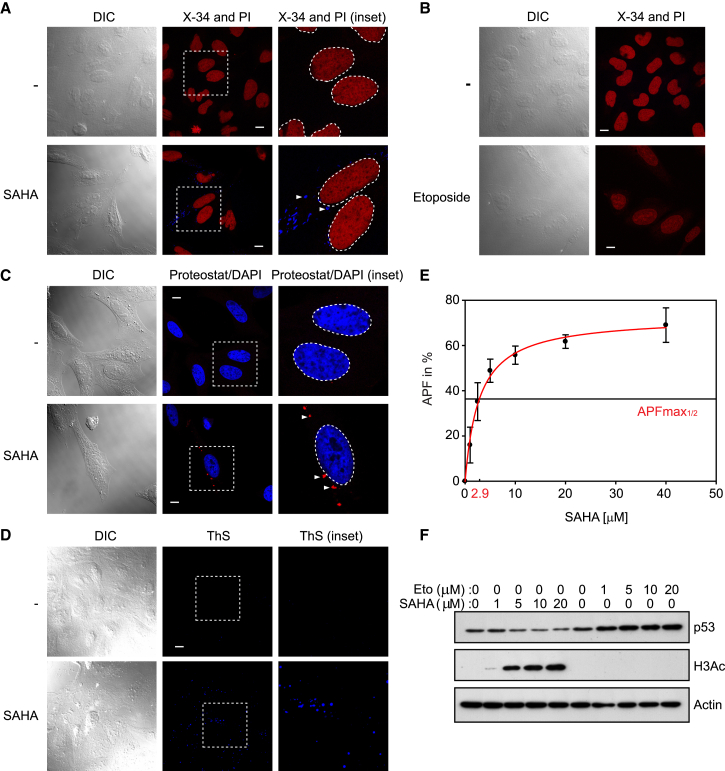
Amyloid-like Protein Aggregates in HDAC Inhibitor-Treated Cells (A) U2OS cells were treated with either DMSO (control, −) or SAHA (5 μM) for 24 hr and stained with the dye X-34 (blue), which specifically recognizes amyloid-like structures. The cells were then fixed, permeabilized, and counterstained with propidium iodide (PI) to visualize the nuclei (red). Nuclei are indicated with white dashed circles. White arrowheads indicate the position of X-34-positive aggregates. Scale bar represents 10 μm. (B) U2OS cells were treated with either DMSO (−) or etoposide (5 μM) for 24 hr and stained with X-34 (blue). The cells were then fixed, permeabilized, and counterstained with PI to visualize the nuclei (red). Scale bar represents 10 μm. (C) U2OS cells were treated with either DMSO as negative control (−) or SAHA (5 μM) for 24 hr, fixed, and permeabilized. The cells were then stained with Proteostat, recognizing aggregated (not necessarily amyloid-like) structures (red), indicated by white arrowheads. Cells were counterstained with DAPI (blue) to visualize their nuclei, indicated here by white circles. Scale bar represents 10 μm. (D) U2OS cells were treated with either DMSO (−) as negative control or SAHA (5 μM) for 24 hr, fixed, and permeabilized. The cells were then stained with thioflavin S solution, which can specifically stain amyloid structures in in situ specimens (blue). Scale bar represents 10 μm. (E) U2OS cells were treated with SAHA inducing the formation of Proteostat-positive aggregates, as shown for instance in (C). Cells were measured by flow cytometry in the FL3 channel and the aggregation propensity factor (APF) was calculated. From at least five independent biological replicates APFs were averaged and APF_max½_ was calculated, indicating that at half-maximal concentration aggregation occurs. Error bars denote SD. (F) Immunoblot corresponding to (A) and (B) measuring acetylated histone H3Ac, demonstrating that the pan-HDAC inhibitor was active and hyperacetylation of proteins appeared. Increasing levels of p53 give a measure for the successful application of etoposide (Eto). β-Actin was used as a loading control. DIC, differential interference contrast. See also Figures S1 and S2.

**Figure 2:**
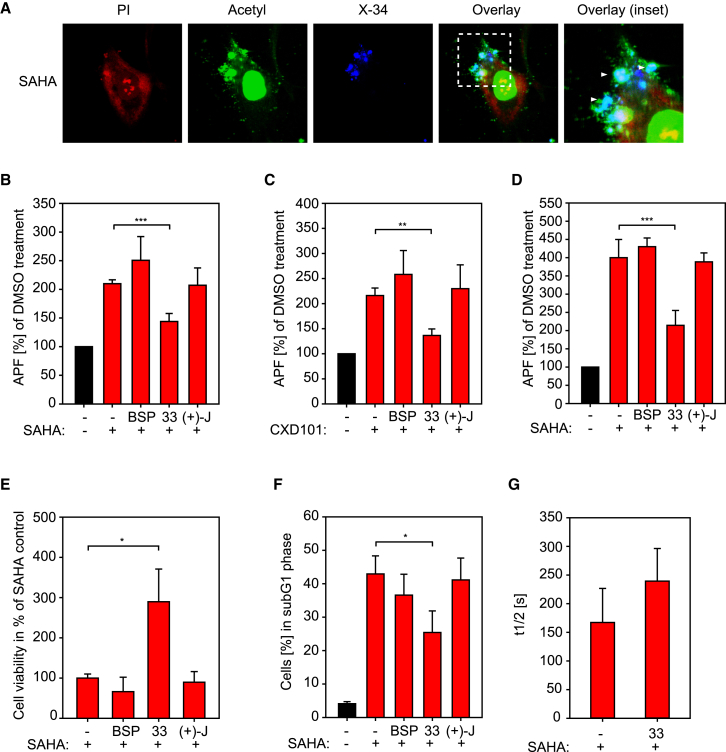
Acetylated Proteins Are Present in the Aggregates, and CBP/p300-Specific BDIs Reduce Aggregation and Cytotoxicity (A) U2OS cells were treated with the pan-HDAC inhibitor SAHA for 24 hr or DMSO (−). Living cells were stained with X-34, then fixed, permeabilized, and treated with an antibody against acetyl-lysine. Propidium iodide (PI) was used as a nuclear counterstain. Arrows indicate X-34 positive aggregates co-localizing with lysine acetylation (see also Figure S2E). (B) U2OS cells were treated with SAHA (5 μM) and BDIs (2.5 μM) as indicated and 24 hr later fixed, permeabilized, and stained with Proteostat. At least 20,000 cells were measured by fluorescence-activated cell sorting (FACS) in the FL-3 channel and the mean fluorescence recorded, which was normalized to the DMSO-only (−) treated control (black); SAHA-treated samples are depicted in red. The data were derived from four independent biological replicates, each performed in three technical replicates, and the aggregation propensity factor (APF) was calculated. Level of statistical significance is indicated (***p ≤ 0.001). Error bars denote SD (see also Figures S3A–S3C). BSP, bromosporine; 33, compound 33; (+)-J, (+)-JQ1. (C) U2OS cells were treated with CXD101 (5 μM) and BDIs (2.5 μM) as indicated and 24 hr later fixed, permeabilized, and stained with Proteostat. At least 20,000 cells were measured by FACS in the FL-3 channel and the mean fluorescence recorded, which was normalized to the DMSO-only (−) treated control (black); CXD101-treated samples are depicted in red. The data were derived from six independent biological replicates, and the APF was calculated. Level of statistical significance is indicated (**p ≤ 0.01). Error bars denote SD. BSP, bromosporine; 33, compound 33; (+)-J, (+)-JQ1. (D) SH-SY5Y cells were treated with SAHA (5 μM) and BDIs (2.5 μM) as indicated and 24 hr later fixed, permeabilized, and stained with Proteostat. At least 20,000 cells were measured by FACS in the FL-3 channel and the mean fluorescence recorded, which was normalized to the DMSO-only (−) treated control (black); SAHA-treated samples are depicted in red. The data were derived from six independent biological replicates, and the APF was calculated. Level of statistical significance is indicated (***p ≤ 0.001). Error bars denote SD. BSP, bromosporine; 33, compound 33; (+)-J, (+)-JQ1. (E) U2OS cells were treated with SAHA (5 μM) and co-treated with different BDIs at 2.5 μM. Cell viability was determined by means of the MTT assay 72 hr after co-treatment. The MTT level of SAHA alone was set to 100% and the data represent five independent biological replicates, each with at least three technical replicates. Level of statistical significance is indicated (*p ≤ 0.05). Error bars denote SD (see also Figure S4). BSP, bromosporine; 33, compound 33; (+)-J, (+)-JQ1. (F) U2OS cells were treated either with DMSO (−) or SAHA (5 μM), and different BDIs (2.5 μM). After harvesting and fixation, cells were stained with PI and the different cell-cycle phases were measured by FACS. The percentage of the cells in the sub-G1 phase is shown. Cells treated with DMSO only are depicted in black and SAHA-treated cells in red. Level of statistical significance is indicated (*p ≤ 0.05). Error bars denote SD. BSP, bromosporine; 33, compound 33; (+)-J, (+)-JQ1. (G) U2OS cells treated with pan-HDAC inhibitor and with DMSO (−) or 33 were stained with X-34 and subjected to FRAP analysis. Shown is the t_1/2_ recovery of the bleached aggregates. At least five independent experiments are represented; error bars denote SD. 33, compound 33. See also Figures S2E, S3, and S4.

We decided to facilitate high levels of lysine acetylation by treating cells with the pan-HDAC inhibitor SAHA. U2OS cells treated with SAHA at increasing concentration and stained with the amyloid-specific dye X-34 (Styren et al., 2000) exhibited inclusion bodies that were amyloid-like aggregates by virtue of their staining with X-34 (Figure 1A); X-34 was chosen because it offers increased sensitivity and stains viable cells (Link et al., 2001). Aggregates were apparent after 24 hr of treatment, and at increasing doses of SAHA they were even more pronounced with X-34 (Figure S1A). To test whether the aggregates could also be visualized by other dyes that detect amyloid-like structures, we compared X-34 with thioflavin S (Games et al., 1995). Amyloid-like aggregates were also observed upon thioflavin S staining of cells treated with SAHA (Figure 1D). Proteostat, which stains protein aggregates, but which are not necessarily amyloid-like (Shen et al., 2011), similarly stained the aggregates (Figures 1C, 1E, S1B, and S1C). Bortezomib treatment was used as a control as it is established to cause protein aggregation reflecting inhibition of the UPS (Hyun et al., 2003) (Figure S1A). Treating cells with a variety of mechanistically unrelated agents, including etoposide, doxorubicin, and 5-fluorouracil (5-FU), did not reveal any aggregates by X-34 staining (Figures 1B, S1A, and S1B; doxorubicin and 5-FU not shown). We confirmed that SAHA was active under these conditions, through increased acetylation of histone H3 after SAHA, but not etoposide treatment (Figure 1F). Conversely, dose-dependent upregulation of p53 occurred after etoposide treatment, contrasting with SAHA treatment which did not alter p53 levels (Figure 1F). The aggregates were observed with non-hydroxamic acid HDAC inhibitors, such as CXD101, and occurred in other cell lines, including the human DLBCL cell lines RIVA and HBL-1 and the neuroblastoma SH-SY5Y cell line (Figures 2, 4, S1, S2, and S5). In summary, dyes that detect protein aggregates in cells, including amyloid-like structures, identified aggregates that depend upon aberrant lysine acetylation.

### Protein Aggregation Decreases upon Treatment with p300/CBP Bromodomain Inhibitors

Given the amyloid-like nature of the aggregates in HDAC inhibitor-treated cells, we were interested to determine whether proteins present in the aggregates were acetylated and, should this be the case, what their identity might be. In cells treated with SAHA the same aggregates co-immunostained with anti-acetyl-lysine antibodies (Figures 2A and S2E). Other cell treatments, including etoposide and bortezomib, did not cause any anti-acetyl-lysine immunostaining (Figure S2E).

We wanted then to address whether the aggregates were a primary event of deregulated lysine acetylation. We reasoned that if they were, then the aggregates should be affected by agents that block lysine acetylation-dependent protein interactions, and considered bromodomains and their inhibitors (BDIs). Thus, we tested whether co-treating cells with HDAC inhibitors and small-molecule BDIs affected the level of protein aggregation, using a collection of BDIs which included the CBP/p300 inhibitors I-CBP112 (Picaud et al., 2015), SGC-CBP30 (Hammitzsch et al., 2015) and its analogs, compounds 17 and 33 (Hay et al., 2014), the BET inhibitor (+)-JQ1 (Filippakopoulos et al., 2010), and the broad-range BDI bromosporine (Theodoulou et al., 2016) (Table S1). Thereafter, cells were stained with Proteostat, and the amount and intensity of staining quantitated by flow cytometry. An increasing dose of SAHA led to a concomitant increase in the number of aggregates, until the level of aggregates plateaued and saturation was achieved (Figures 1E, S1C, and S2), from which APF (aggregation propensity factor) and AC_50_ (50% aggregation concentration) was determined (Figures 1E and S2A). Treatment of cells with each BDI alone did not result in the appearance of aggregates (data not shown). Remarkably, however, in SAHA- or CXD101-treated cells, co-treatment with some of the BDIs decreased the amount and number of aggregates (Figures 2, 4, S3, and S5). Specifically, BDI 17, 33, and SGC-CBP30 specific for the p300/CBP bromodomains (Table S1) reduced the amount of aggregation, whereas BDIs targeting other bromodomain families such as BET proteins had no discernible effect (Figures 2, 4, S3, and S5). We also investigated whether the BDIs had an influence on the stability and building rate of SAHA-induced aggregates using a fluorescence recovery after photobleaching (FRAP) assay. The results derived from cells treated with SAHA with or without the most potent p300/CBP BDI compound 33 (Figure 2) suggested that the half recovery time (t_1/2_) of the material into aggregates increased compared with control treatment (Figure 2G), implying that formation of the aggregates could be delayed when the p300/CBP bromodomain was inhibited.

We then assessed the effect of bromodomain inhibition on cell viability in cells treated with SAHA. The p300/CBP BDIs that reduced aggregate formation, namely BDI 17, 33, and SGC-CBP30, were similarly able to rescue cells from the cytotoxic effect of SAHA treatment and there was little effect of the other BDIs (Figures 2E and S4). Notably, bromosporine had the opposite effect, since it acted additively with SAHA to reduce cell viability even further (Figures 2E and S4). Cell-cycle analysis indicated that the normal cell-cycle profile was partially restored when SAHA-treated cells were co-treated with the p300/CBP BDIs, but not with the other compounds (Figure 2F). Thus, aggregate formation and cytotoxicity are diminished upon treatment with p300/CBP-specific BDIs.

### p300/CBP Bromodomains Co-localize with Aggregates and Regulate Aggregate Formation

**Figure 3:**
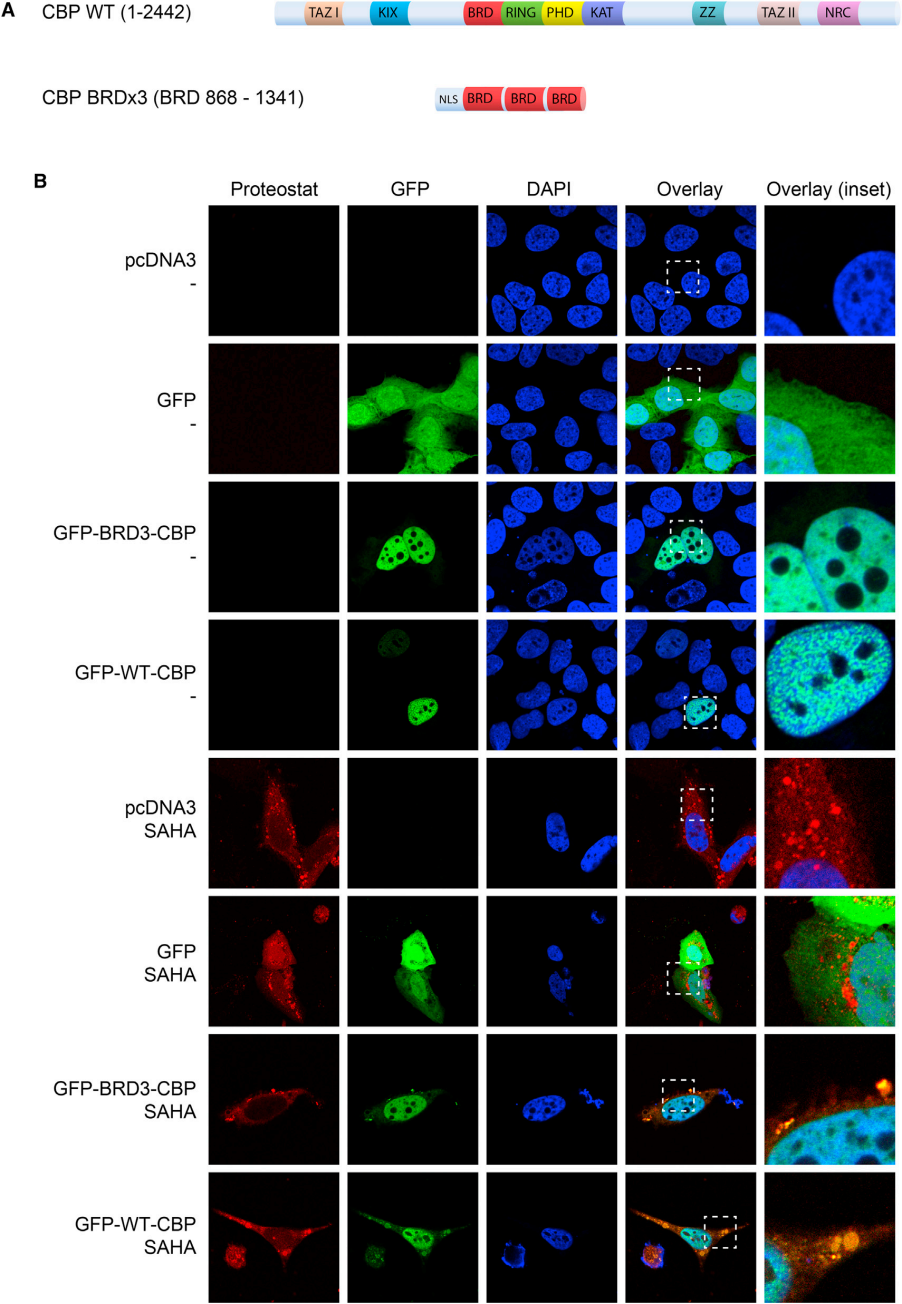
Bromodomains of p300/CBP Are Directly Involved in the Formation of Aggregates upon HDI Treatment (A) Schematic overview of the CBP full-length protein (1–2,442) and the CBP 3× bromodomain construct (868–1,341 for BRD). In the upper panel, wild-type (WT) CBP (1–2,442) is shown with all its domains; in the lower panel CBP BRD3 (868–1,341 for BRD) is shown, which consists only of three tandem bromodomains with an N-terminal NLS sequence. Both constructs are tagged with GFP. (B) U2OS cells were transfected with an empty vector (pcDNA3), GFP, full-length WT GFP-CBP (1–2,442), or GFP-BRD3 (868–1,341), treated 1 day later with DMSO (−) or SAHA (5 μM), and 24 hr later fixed, permeabilized, and stained with Proteostat. Representative examples of images are shown. Panels on the right represent the enlarged area shown by a dashed square on the panels to the left, and aggregates which co-localize with GFP-tagged full-length WT CBP (1–2,442) or 3BRD-GFP (868–1,341) appear as yellow/orange in the images. DAPI (blue) was used as a nuclear counterstain.

The effects of BDI treatment suggested that p300/CBP proteins are mechanistically involved in the acetylation-dependent aggregation process. We therefore tested whether expressing wild-type (WT) CBP, or a derivative containing the bromodomain, 3BRD, expressed at levels similar to that of WT CBP, could be incorporated into the aggregates (Figures 3A and 3B). In untreated cells, both WT CBP and 3BRD proteins localized to the nucleus, with a small proportion of the protein retained in the cytoplasm (Figure 3B). However, the amyloid-like aggregates that appeared in the cytoplasm of cells treated with SAHA co-localized with both WT CBP and 3BRD (Figure 3B); the unfused GFP protein was distributed evenly throughout the cell without any propensity to aggregate under SAHA treatment conditions (Figure 3B).

**Figure 4:**
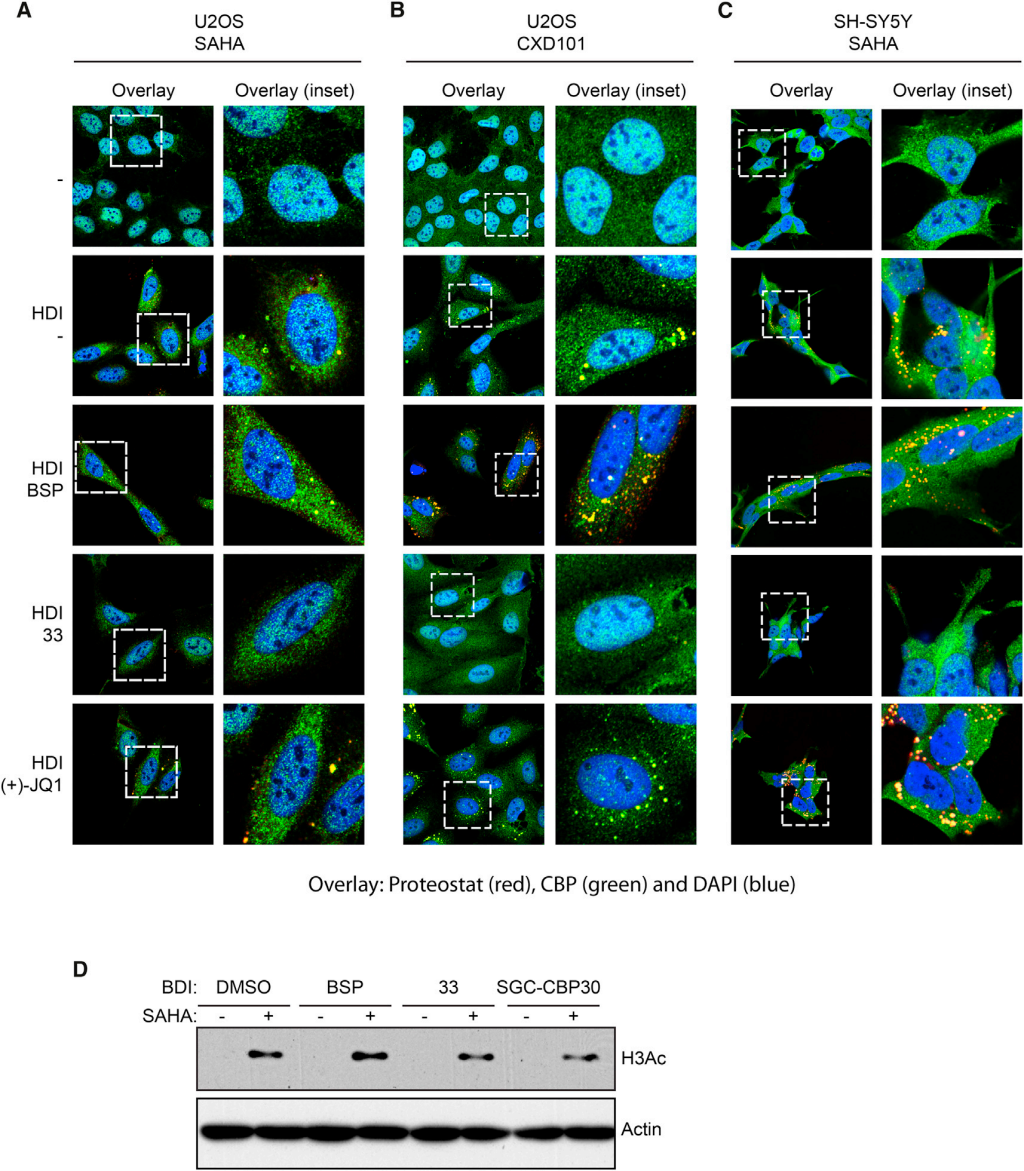
p300/CBP-Specific BDIs Reduce the Formation of Aggregates upon Hyperacetylation (A) U2OS cells were treated with SAHA (5 μM) or DMSO (−) with or without the indicated BDIs (2.5 μM). After 24 hr the cells were fixed, permeabilized, and stained with Proteostat (red), anti-CBP (green), and DAPI (blue) as a nuclear counterstain, and analyzed by confocal microscopy. Panels on the right of each pair represent the enlarged area shown by a dashed square on the left panels. Aggregates stained with Proteostat that co-localize with CBP appear as yellow/orange in the images. BSP, bromosporine; 33, compound 33. (B) U2OS cells were treated with CXD101 (5 μM) or DMSO (−) with or without the respective BDIs (2.5 μM). After 24 hr the cells were fixed, permeabilized, and stained with Proteostat (red), anti-CBP (green), and DAPI (blue) as a nuclear counterstain, and analyzed by confocal microscopy. Panels on the right of each pair represent the enlarged area shown by a dashed square on the left panels. Aggregates stained with Proteostat that co-localize with CBP appear as yellow/orange in the images. BSP, bromosporine; 33, compound 33. (C) SH-SY5Y cells were treated with SAHA (5 μM) or DMSO (−) with or without the respective BDIs (2.5 μM). After 24 hr the cells were fixed, permeabilized, and stained with Proteostat (red), anti-CBP (green), and DAPI (blue) as a nuclear counterstain, and analyzed by confocal microscopy. Panels on the right of each pair represent the enlarged area shown by a dashed square on the left panels. Aggregates stained with Proteostat that co-localize with CBP appear as yellow/orange in the images (see also Figure S5). BSP, bromosporine; 33, compound 33. (D) Corresponding immunoblot measuring acetylated histone H3Ac, which demonstrates that the pan-HDAC inhibitor was active, the appearance of hyperacetylation of proteins, and the influence of two CBP/p300-specific BDIs. U2OS cells were treated with SAHA (5 μM) and the respective BDIs (2.5 μM) and then harvested after 24 hr. β-Actin was used as a loading control. See also Figures S3 and S5.

**Figure 5:**
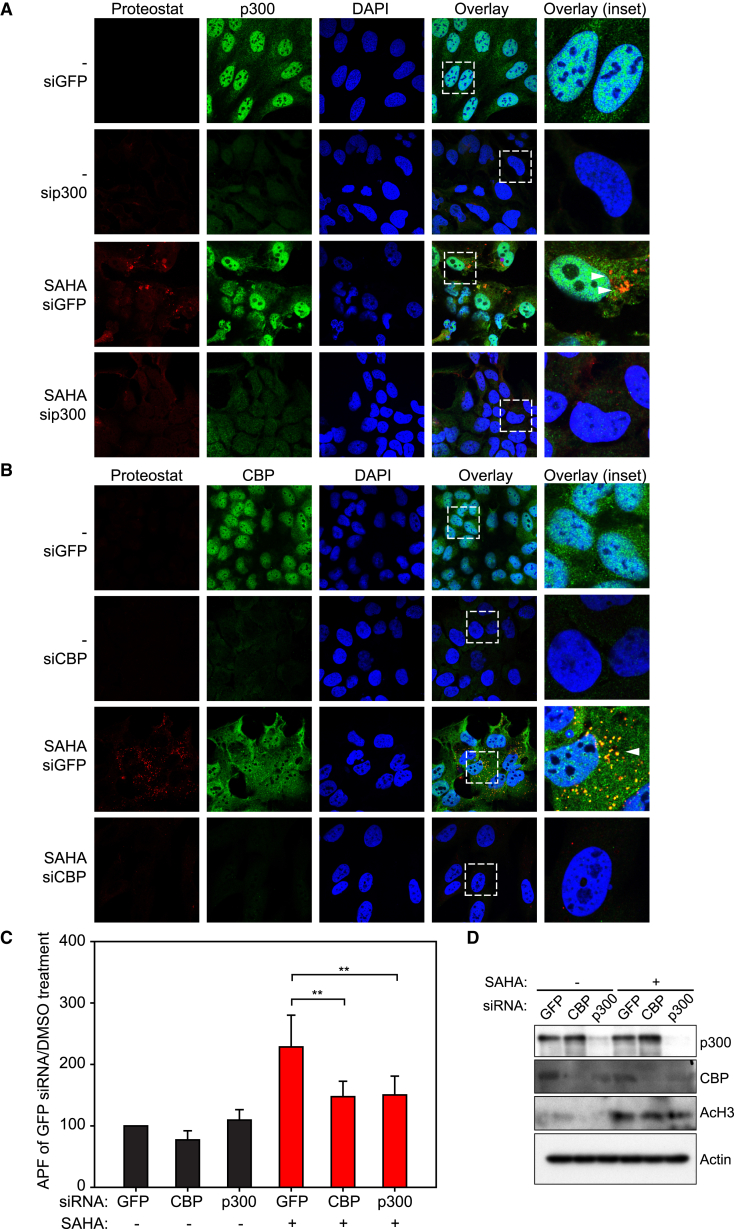
Depletion of CBP/p300 Reduces the Formation of Aggregates upon HDI Treatment (A) U2OS cells were transiently transfected with either a control siGFP or sip300 for 4 days. Twenty-four hours before fixation, cells were treated with SAHA. Cells were stained with Proteostat (red channel) and anti-p300 (green channel), and DAPI was used as a nuclear counterstain (blue). Arrowheads indicate Proteostat-positive aggregates. Co-localization of Proteostat-positive aggregates with p300 appears orange/yellow in the overlay. Panels on the right of each pair represent the enlarged area shown by a dashed square on the panels to the left. (B) U2OS cells were transiently transfected with either a control siGFP or siCBP for 4 days. Twenty-four hours before fixation, cells were treated with SAHA. Cells were stained with Proteostat (red channel) and anti-CBP (green channel), and DAPI was used as a nuclear counterstain (blue). Arrowheads indicate Proteostat-positive aggregates. Co-localization of Proteostat-positive aggregates with CBP appears orange/yellow in the overlay. Panels on the right of each pair represent the enlarged area shown by a dashed square on the panels to the left. (C) U2OS cells were treated with siRNAs for 4 days (control siGFP, sip300, or siCBP) and subsequently with SAHA (5 μM), and 24 hr later fixed, permeabilized, and stained with Proteostat. At least 20,000 cells were measured by FACS and the mean fluorescence recorded, which was normalized to the siGFP/DMSO-only (−) treated control. The data were derived from six independent biological replicates and the aggregation propensity factor (APF) was calculated. SAHA-treated cells are depicted in red and DMSO-treated cells in black. Level of statistical significance is indicated (**p ≤ 0.01). Error bars denote SD. (D) Immunoblot showing the protein level of CBP and p300 in siRNA-treated cells. Cells were treated for 4 days and 24 h before lysis, DMSO (−) or SAHA was added. β-Actin was used as a loading control, and an antibody against acetylation of the N terminus of histone 3 served as a control for the effect of SAHA.

Following on, we assessed whether endogenous p300/CBP was present and found both p300 and CBP to similarly co-localize with the aggregates upon SAHA treatment in different cell lines (Figures 4 and S5). Most significantly, co-treatment with p300/CBP-specific BDIs reduced the amount of amyloid-like aggregates and co-localization with p300/CBP, whereas the other inhibitors had little effect (Figures 4 and S5). The active BDIs caused a partial recovery of p300/CBP to nuclei (Figures 4 and S5), and similar effects were observed with SAHA and CXD101 treatment and in different cell lines (Figures 4 and S5). Moreover, the effect of p300/CBP BDIs was unlikely to be mediated through general effects on acetylation, because analysis of the acetylation level of a number of proteins, such as histone H3, failed to show any significant effect (Figure 4D). Furthermore, we silenced p300 and CBP using small interfering RNA (siRNA), which caused a significant reduction in aggregation upon treatment of the cells with SAHA compared with the control siRNA treatment (Figures 5A and 5B). We quantitated the effect by flow cytometry, which indicated that the APF in p300 or CBP silenced cells was significantly decreased (Figures 5C and 5D). Thus, amyloid-like aggregation in SAHA-treated cells occurs in a fashion dependent upon p300/CBP bromodomains.

### Amyloid-like Aggregates Harbor Components of the Proteostatic Machinery

**Figure 6:**
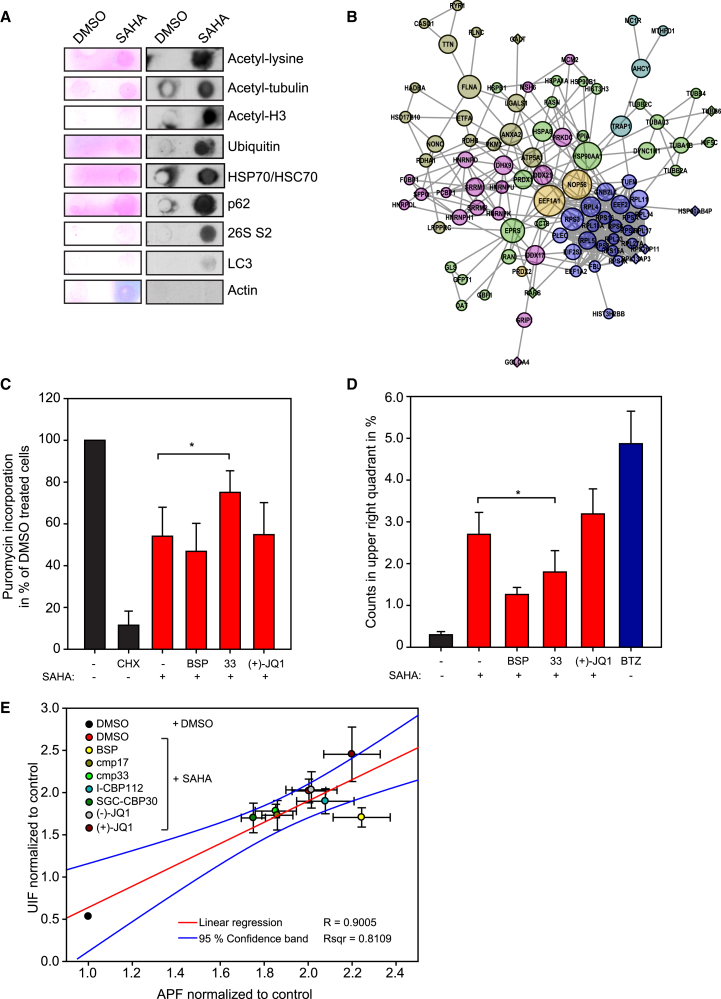
Composition of the HDAC Inhibitor-Induced Aggregates and Effect of CBP/p300-Specific BDIs (A) U2OS cells were treated with either DMSO or SAHA (5 μM) for 24 hr and then harvested; membranes and DNA were removed and the remaining aggregate fraction treated with detergent. The material captured in the filter retardation assay was visualized by Ponceau S staining or immunostaining with the indicated antibodies (see also Figures S6A and S6B). (B) U2OS cells were treated with either DMSO or SAHA (5 μM) for 24 hr and then harvested; membranes and DNA were removed and the remaining aggregate fraction treated with detergent. The aggregated proteins were trypsinized and subjected to tandem mass spectrometry analysis. Proteins were grouped according to their biological role, which is symbolized by the colors. The size of the symbols represent the betweenness-centrality of the proteins, and the circles represent proteins known to be acetylated, either determined to be so in this study or by mining Phosida, UniProt, MaxQB, CPLM, and PhosphoSitePlus (see also Figure S6A). (C) Cells were treated with SAHA (5 μM) and the indicated BDIs. The effect on mRNA translation was visualized by incorporation of puromycin in the nascent polypeptide chain after immunoblotting with a monoclonal antibody against puromycin in polypeptide chains. For comparison, Ponceau S served as a loading control. The intensities of the anti-puromycin signal in the different lanes were quantified and normalized against the intensities of the Ponceau S staining. The average of five independent experiments is shown, normalized to puromycin incorporation in percentage of DMSO (−)-treated cells. Level of statistical significance is indicated (*p ≤ 0.05). Error bars denote SD. Quantification of the independent biological replicates as shown in Figure S7A. BSP, bromosporine; 33, compound 33; (+)-J, (+)-JQ1. (D) HEK293T cells stably expressing Ub-EGFP were treated with 5 μM of the HDAC inhibitor and with the indicated BDIs. Cells were then analyzed by flow cytometry; green fluorescence (FL1 channel) of the Ub-EGFP is plotted on the y axis and red fluorescence of Proteostat staining (FL3 channel) on the x axis, as shown in Figure S7B. Quadrants were chosen according to the DMSO (−) control, which is set to the lower left quadrant, and the FL3 mean fluorescence in the upper right quadrant of the gated cells was quantified. Shown are three biological replicates, each in technical triplicates. Level of statistical significance is indicated (*p ≤ 0.05). Error bars denote SD. BSP, bromosporine; 33, compound 33; (+)-J, (+)-JQ1. (E) HEK293T cells stably expressing Ub-EGFP were treated with SAHA (5 μM) and the indicated BDIs (2.5 μM). Cells were then fixed, permeabilized, and stained with Proteostat. FACS was carried out and the cells were measured in the FL1 channel to detect accumulated Ub-EGFP and in the FL3 channel to detect Proteostat-positive aggregates. After gating and compensation, the mean fluorescence intensities in the upper right quadrants were plotted against each other. The data were derived from at least three independent biological replicates, each in technical triplicates. Linear regression resulted in R^2^ = 0.8109. BSP, bromosporine. See also Figures S6 and S7.

Although the results suggest that p300/CBP bromodomains are involved in aggregate formation, we were interested to characterize the aggregate proteome. Thus, we devised a biochemical filter-based procedure to purify aggregates from SAHA-treated cells, and subjected the purified material to a candidate-based investigation followed by unbiased mass spectrometry (Figure S6A). As expected, the aggregates contained proteins known to undergo lysine acetylation, such as α-tubulin and histone H3 (Figures 6A and S6B). Proteins involved in diverse aspects of proteostasis were present, such as the chaperones heat shock protein 70/heat shock cognate protein 70 (HSP70/HSC70), the autophagy regulator p62 (Bjorkoy et al., 2005), and the proteasomal component 26S S2 subunit, in addition to ubiquitinated proteins (Figures 6A and S6B). Some of these proteins co-localized with the aggregates, for example p62, where its co-localization increased with SAHA concentration (Figure S6C).

The candidate-based approach was supplemented with an unbiased proteomics analysis of the composition of the aggregates by mass spectrometry, whereby a broader group of proteins, many still connected with proteostasis, was identified (Figure 6B). A major functional protein cluster centered around chaperone function, including HSP70 and HSP90 (Figure 6B), and another cluster was connected with protein translation, with ribosome subunits and other components of the translation machinery, such as eukaryotic translation elongation factor 1α (Figure 6B). Analysis of the acetyl-lysine content found that many of the proteins had a high content of lysine residues and had been previously shown to be acetylated (Table S2), including ribosomal proteins, proteins associated with translation such as EEF1A1, EEF1A2, and TUFM (Tu translation elongation factor, mitochondrial), molecular chaperones in the HSP70 and HSP90 family, and histones (Table S2). The aggregate proteome showed on average an increased percentage of lysine residues per protein, namely 7.5% compared with 5.5% or 5.8% in the total cellular proteome (Jones et al., 1992, Tourasse and Li, 2000). It is possible that the high acetyl-lysine content explains their propensity to form aggregates.

### Lysine Acetylation Affects Translation and UPS Function

The fact that many proteins involved in protein translation were found in the aggregates led us to hypothesize that protein acetylation and aggregate formation affects protein translation. We therefore tested whether protein translation was affected in SAHA-treated cells and the effect of co-treatment with the p300/CBP BDIs. SAHA-treated cells exhibited a strong decrease in protein translation (Figures 6C and S7A), measured by the incorporation of puromycin into nascent polypeptide chains (Schmidt et al., 2009). Compared with the control treatment, there was an approximate 50% reduction in the occurrence of nascent polypeptide chains when cells were treated with SAHA (Figures 6C and S7A). The p300/CBP BDIs were also able to restore protein translation up to 80%, contrasting with the minimal effect of the other BDIs (Figures 6C and S7A). It appears, therefore, that formation of protein aggregates upon pan-HDAC inhibitor treatment and the role of p300/CBP are linked to protein translation.

We also assessed whether the UPS as part of the cellular proteostasis was altered in SAHA-treated cells and thereafter, the role of bromodomain proteins. We measured the activity of the UPS by means of the expression level of a reporter protein Ub-EGFP, which accumulates upon UPS impairment (Figures 6D and S7B). Significantly, SAHA treatment caused an increase in the Ub-EGFP signal, indicating that UPS activity was defective (Figures 6D and S7B). We then investigated whether there was a statistical relationship between UPS activity and formation of amyloid-like aggregates upon HDAC inhibitor treatment, with and without co-treatment with BDIs. Staining with Proteostat increased after treatment of the stable Ub-EGFP cells with SAHA (Figures 6D and S7B). The Ub-EGFP cells were also co-treated with BDIs, then stained with Proteostat and analyzed by flow cytometry, together with the level of Ub-EGFP. The data are presented graphically as UPS impairment (upper left and upper right quadrant) and formation of aggregates (lower right and upper right quadrant) in the presence and absence of co-treatment with BDI (Figure S7B). There was a linear relationship between the level of amyloid-like aggregates and UPS impairment (Figure 6E), suggesting a functional relationship between the two parameters. Furthermore, the p300/CBP BDIs were most effective at relieving UPS impairment, whereas the other BDIs had insignificant effects (Figure 6E). The coincidental relationship between the effect of p300/CBP BDIs on aggregate formation and UPS impairment argues strongly that p300/CBP bromodomains are linked to the UPS through protein aggregation.

### Huntingtin Aggregates and Bromodomain Inhibitors

**Figure 7:**
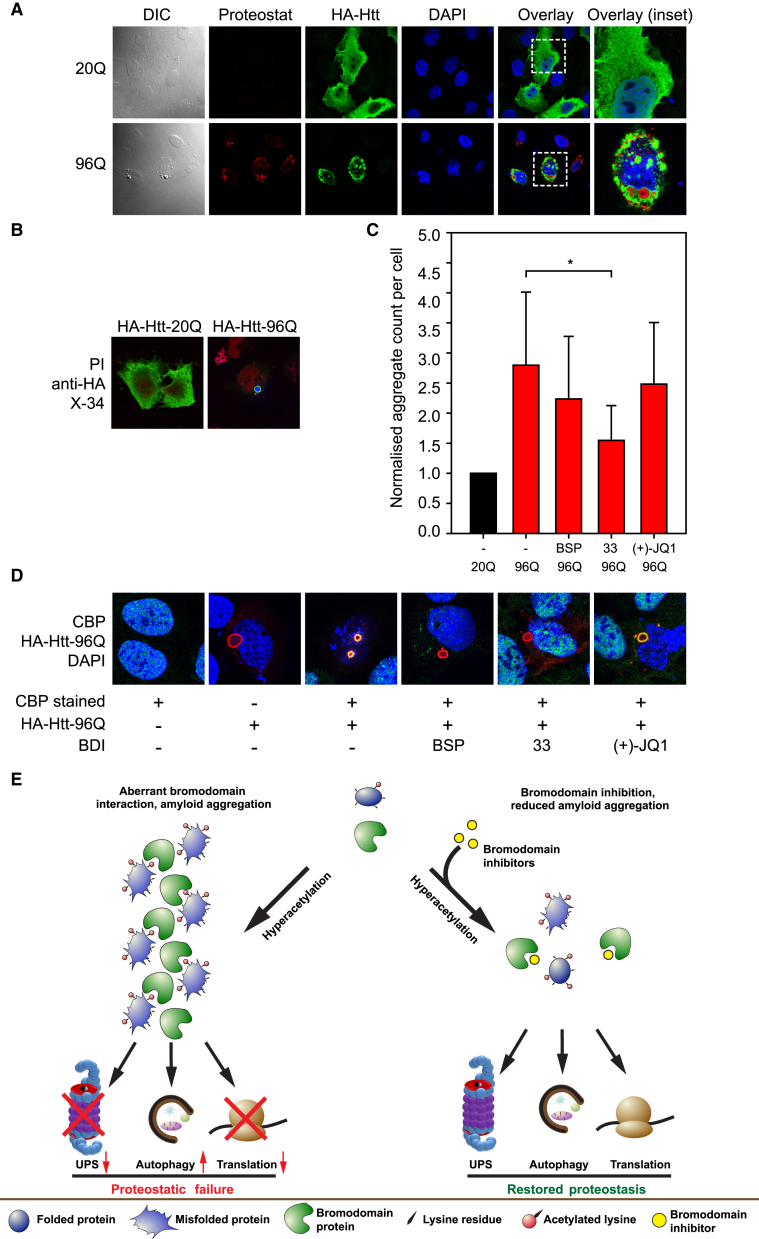
Effect of BDIs on Pathologically Elongated Huntingtin (A) U2OS cells expressing either the exon 1 of huntingtin (Htt) with 20Q or 96Q were fixed, permeabilized, and stained with an HA antibody (shown in green). Proteostat was applied to visualize aggregated structures (red), and nuclei were counterstained with DAPI (blue). Panels on the right represent the enlarged area shown by a dashed square on panels to the left. (B) U2OS cells expressing HA-Htt-96Q exon 1 form aggregates and are positively stained with X-34 (shown in blue). Nuclei were stained with propidium iodide (PI) (red) and the Htt constructs are shown in green. (C) U2OS cells expressing HA-Htt-96Q or HA-Htt-20Q were treated with the respective BDI (2.5 μM), then fixed, permeabilized, and stained with Proteostat and DAPI as a nuclear counterstain. The number of Proteostat-positive aggregates was then quantified with an IN Cell Analyzer 1000. The data were derived from at least three independent experiments each in triplicates, and for each condition at least 1,500 cells were analyzed relative to 20Q, which was given an arbitrary value of 1. Level of statistical significance is indicated (*p ≤ 0.05). Error bars denote SD. The sample expressing HA-Htt-20Q is depicted in black, and HA-Htt-96Q-expressing cells are depicted in red. BSP, bromosporine; 33, compound 33; (+)-J, (+)-JQ1. (D) U2OS cells expressing HA-Htt-96Q were treated with either DMSO (−) or BDIs (2.5 μM), then fixed, permeabilized, and stained with an antibody against CBP (green), HA (red), and DAPI (blue) as a nuclear counterstain. Co-localization of HA-Htt-96Q aggregates with endogenous CBP is depicted by yellow/orange color. Shown are the overlays from the three different channels. BSP, bromosporine; 33, compound 33; (+)-J, (+)-JQ1. (E) Schematic overview of protein aggregation and proteostatic failure upon hyperacetylation and rescue by bromodomain inhibitors. Proteins aggregate upon hyperacetylation and aberrant interactions occur with bromodomain-containing proteins such as CBP/p300. Essential protein quality control mechanisms are affected, such as degradation of proteins via the ubiquitin proteasome system and protein translation. BDIs, and especially those preferentially binding to CBP/p300, diminish the formation of aggregates and also restore to some extent protein degradation and translation. See also Figure S7.

The pathologically elongated form of huntingtin (Htt) contains a trinucleotide expansion resulting in a polyglutamine extension that has a propensity to form amyloid aggregates (DiFiglia et al., 1997). CBP has been suggested to be involved in forming Htt aggregates (Nucifora et al., 2001, Steffan et al., 2000). Given the results presented here highlighting the role of p300/CBP proteins in the formation of amyloid-like protein aggregates, we tested whether the p300/CBP BDIs could affect Htt aggregates. In U2OS cells expressing either Htt with a 20Q (hemagglutinin [HA]-Htt-20Q) or 96Q stretch (HA-Htt-96Q), Htt-20Q was evenly distributed in cells with no signs of aggregation, whereas cells expressing Htt-96Q exhibited aggregates (Figures 7A and 7B). Htt-96Q occurred as large aggregates in both the nucleus and cytoplasm and stained with Proteostat, whereas HA-Htt-20Q did not (Figure 7A). HA-Htt-96Q could be also stained with X-34, indicating an amyloid-like structure (Figure 7B). Cells expressing either Htt-20Q or Htt-96Q were treated with each BDI, and the number of aggregates measured in the IN Cell Analyzer. Significantly, p300/CBP BDIs caused a modest but reproducible level of inhibition of Htt-96Q aggregation (Figure 7C); immunoblotting of Htt-96Q showed little difference in the protein level (Figure S7C), suggesting that the effect was due to the direct effect of p300/CBP BDIs on the aggregation process. Since CBP interacts with Htt (Nucifora et al., 2001, Steffan et al., 2000), we also addressed whether treatment with the p300/CBP BDIs could influence the interaction. Both endogenous CBP as well as ectopic WT CBP co-localized with the Htt-96Q aggregates (Figures 7D and S7D). Significantly, treatment with the p300/CBP BDIs reduced the level of co-localization (Figures 7D and S7D). Thus, the results suggest that p300/CBP bromodomains are involved in formation of the Htt-96Q aggregates.

## Discussion

Amyloid fibrils are insoluble fibrous protein aggregates that contribute to a variety of diseases, including Alzheimer's disease, Parkinson's disease, and Huntington's disease (Chiti and Dobson, 2006, Dobson, 2003). It has been suggested that amyloid-like aggregates provide a protective role that the cell imposes to overcome the toxic effects of protein misfolding (Arrasate et al., 2004), but in doing so the aggregates disturb proteostasis and other cellular functions, leading to cell death (Olzscha et al., 2011). Because amyloid-like aggregates occur in a wide variety of tissues and diseases, and in addition share common structural features, there has been a great deal of interest in developing therapeutic agents that inhibit their formation and limit their toxicity (Powers et al., 2009).

An important conclusion from this study is that hyperacetylation of proteins has a global effect on amyloid-like protein aggregation and causes a general proteostatic failure (Figure 7E). Protein hyperacetylation occurs naturally in cells, for example, by cell stressors such as reactive oxygen species (Mackeh et al., 2014) and upon starvation (Geeraert et al., 2010). Our ability to define the aggregates as amyloid-like reflected their staining with different dyes specific for amyloid-like structures and protein aggregates (Games et al., 1995, Shen et al., 2011, Styren et al., 2000). The aggregates contained a concentration of acetylated proteins, and through a chemical biology-led approach using small-molecule BDIs with pre-defined specificities (Filippakopoulos et al., 2010, Hay et al., 2014, Picaud et al., 2015), we established the p300/CBP bromodomain protein family to be mechanistically and functionally involved in the aggregation process. Most notably, small-molecule inhibitors with specificity for the p300/CBP bromodomain family reduced the appearance of the aggregates, suggesting that the integrity and function of p300/CBP bromodomains is rate limiting in forming the aggregates. Furthermore, the presence of the aggregates coincided with impairment of the UPS and protein translation. The results therefore suggest that the proper balance between acetylation and deacetylation of lysine residues is required to maintain protein homeostasis in the cell.

Our understanding of the role of protein aggregation and amyloid formation has highlighted more than 50 human diseases where protein misfolding and conversion into amyloid-like aggregates plays a role in the pathology (Chiti and Dobson, 2006). Surprisingly, there appears to be little similarity in the overall sequence of the proteins identified in amyloid-like aggregates, and it has been suggested that the amyloid structure might be adoptable by virtually any polypeptide able to acquire a generic structure rich in β sheet (Dobson, 2001). The characterization of the amyloid-like aggregates described here indicates that they contain a high level of acetylated lysine proteins, as well as a variety of components connected with diverse aspects of protein quality control, and further that CBP/p300 bromodomains are directly involved in the aggregation process. The high content of lysine residues of many proteins in the aggregates may explain why they are susceptible to protein hyperacetylation upon treatment with HDAC inhibitors and more prone to subsequent aberrant interactions with other proteins.

An analysis of Htt allowed us to assess the impact of lysine acetylation on the aggregates formed by a pathologically relevant protein (DiFiglia et al., 1997). Expansion of a CAG trinucleotide repeat in pathologically mutant forms of Htt leads to the presence of extended polyglutamine tracts, which have been suggested to sequester CBP and related proteins, possibly through the polyglutamine region in CBP, and alter their normal biological roles (McCampbell et al., 2000, Nucifora et al., 2001, Steffan et al., 2000). Because of the relationship that we established between p300/CBP proteins and the amyloid-like aggregates, we reasoned that p300/CBP BDIs might affect the aggregates formed by an expanded Htt. Significantly, the CBP/p300-specific BDIs had a modest but reproducible effect on the Htt aggregates, suggesting that p300/CBP bromodomains contribute to aggregate formation of the elongated form of Htt and, therefore, the pathology of the disease. In turn, these results suggest that BDI-based drugs might provide a therapeutic strategy for treating Huntington's disease and related pathologies (Kirilyuk et al., 2012, Min et al., 2015, Nucifora et al., 2001, Steffan et al., 2000).

Our results established a correlation between amyloid-like aggregates caused by aberrant lysine acetylation and deficits in the UPS and translational activity, suggesting that the protein aggregation causes a global perturbation in proteostasis. The fact that p300/CBP proteins are rate limiting for amyloid-like aggregate formation provides further support for a causative relationship between lysine acetylation and readers of the acetylation mark. Furthermore, our results also bear on the mechanisms through which the cytotoxic effects of HDAC inhibitors are mediated by implying that global effects on protein aggregation make an important contribution. Moreover, protein aggregation may occur in normal cells and thereby contribute to the side effects of HDIs noted in clinical studies (New et al., 2012).

In conclusion, our study describes lysine acetylation, in addition to its role as a mediator of specific regulatory protein interactions through its “reader-writer” interplay, as a process that influences protein aggregation, thereby affecting proteostasis and cell viability. Our results thus highlight a new function for lysine acetylation as a global regulator of cellular proteostasis, and further suggest a role in diseases where aberrant protein folding contributes to the pathology.

## Significance

**Acetylation is one of the most common post-translational modifications of proteins. We demonstrate that hyperacetylation of proteins leads to amyloid-like aggregation involving proteins that function in proteostasis. p300/CBP bromodomain proteins are involved in the aggregation process, and p300/CBP BDIs partially abrogate protein aggregation. Significantly, protein aggregation coincides with defective proteostasis, which is relieved by p300/CBP BDIs. Importantly, the aggregation of Htt with an elongated polyglutamine stretch is influenced by p300/CBP BDIs. These results highlight a global role for acetylation and bromodomain readers in protein aggregation and proteostasis, and suggest that p300/CBP BDIs have potential in the treatment of protein-misfolding diseases.**

## Experimental Procedures

Further details of the procedures used in this study are described in Supplemental Experimental Procedures.

### Plasmids

A cell line stably expressing Ub(G76V)-EGFP in HEK293T cells was used for the experiments and was kindly provided by F.-Ulrich Hartl. pEGFP-N1 was used as a control plasmid for EGFP expression only. HA-tagged Htt constructs with 20Q and 96Q stretches were kindly provided by F.-Ulrich Hartl. GFP-tagged CBP constructs or GFP-tagged bromodomain constructs were created via the MultiSite Gateway System (Life Technologies, 12537100). The construct containing the 3× bromodomain from CBP contains an NLS sequence at the N terminus (Philpott et al., 2014).

### Bromodomain Inhibitors

The different BDIs are listed in Table S1 and have been described previously (Filippakopoulos et al., 2010, Hay et al., 2014, Picaud et al., 2015).

### Cell Culture and Transient Transfection

Human cells were cultured at 37°C in a humidified 5% CO_2_ incubator in DMEM (Sigma) (HEK293T and U2OS) containing 10% fetal bovine serum (FBS) (Labtech), 100 IU/mL penicillin G, and 100 mg/mL streptomycin sulfate (Gibco, Life Sciences, 15140122). HEK293T cells stably expressing Ub-EGFP were maintained in DMEM (Sigma, D6249) containing 10% FBS (Labtech), 100 IU/mL penicillin G, 100 mg/mL streptomycin sulfate (Gibco, Life Sciences), and G418 (Santa Cruz, sc-29065). RIVA and HBL-1 cells were maintained in RPMI (Sigma, R8758) supplemented with the same. SH-SY5Y cells were maintained in Ham's F12/Eagle’s minimal essential medium (EBSS) (1:1) (Sigma, M4655 and N6658), 2 mM glutamine (Gibco, Life Sciences), 1% non-essential amino acids (Gibco, Life Sciences), 15% FBS (Labtech), 100 IU/mL penicillin G, and 100 mg/mL streptomycin sulfate (Gibco, Life Sciences). Transient transfection was performed with GeneJuice (Novagen, 70967-4) according to the manufacturer's protocol.

### Immunofluorescence Staining

Cells were seeded onto coverslips and after different treatments washed once in PBS and then fixed in 4% paraformaldehyde for 15–45 min. Cells were then permeabilized in PBS containing 0.5% Triton X-100 for 5–30 min, depending on the following applications. Cells were washed twice with PBS and incubated for 20 min in 1% BSA in PBS as blocking reagent. Cells were then incubated overnight with the primary antibodies at 4°C in 1:50 to 1:100 dilutions dependent on the antibody. They were washed three times with PBS for 5 min and incubated for 1–2 hr with the corresponding secondary fluorescence antibodies Alexa Fluor 488 (Invitrogen) or Alexa Fluor 594 (Invitrogen) in a 1:400 dilution at room temperature. Cells were then incubated for 10 min in a 1 μg/mL DAPI solution (ThermoScientific). Cells were washed three times for 10 min in PBS, then washed for 5 s three times in distilled water and covered with fluorescence mounting medium (Dako). For visualization of aggregates in the human cell lines, cells were stained with X-34, a fluorescent derivative of Congo red, which has the advantage that it can pass cell membranes and should specifically detect amyloid-like aggregates (Styren et al., 2000). X-34 was synthesized at the core facility of the Max Planck Institute of Biochemistry and its Department of Cellular Biochemistry (F.-Ulrich Hartl and Karsten Klage). X-34 was dissolved in 1 M Tris solution (pH 8.8), and a drop of 5 M NaOH was added to stabilize the product in the dissolved state and prevent crystallization. From this 1-mM stock solution, a 50-μM working solution in DMEM without fetal calf serum was produced and living cells were incubated for 45 min with the X-34 solution. Cells were then washed twice for 10 min with pre-warmed PBS, then fixed and permeabilized as described above. Propidium iodide at a concentration of 1 μg/mL was used to counterstain X-34-treated cells.

Cells were fixed and permeabilized as described above, and Proteostat (Enzo Life Sciences) was added in a 1:2,000 dilution for 30 min. Cells were then counterstained with DAPI and washed three times with PBS for 10 min after they were mounted as described by Shen et al. (2011). Thioflavin S staining of cells was performed according to the improved protocol (Sun et al., 2002), except that cells were fixed and permeabilized as described above with KMnO_4_ and NaOH steps being omitted.

## Author Contributions

H.O. designed and performed the experiments and wrote the manuscript. O.F. and S.K. provided the inhibitors and B.M.K. provided the mass spectrometry expertise. N.B.l.T. conceived the project, directed the research, and wrote the manuscript.

## Supplemental Information

Document S1. Supplemental Experimental Procedures, Figures S1–S7, Tables S1 and S2, and Supplemental References

Document S2. Article plus Supplemental Information
