# Supplementary material for: CBP/p300 Bromodomains Regulate Amyloid-like Protein Aggregation upon Aberrant Lysine Acetylation
Source: Cell Chem Biol. 2017 Jan 19;24(1):9–23. doi: 10.1016/j.chembiol.2016.11.009 (PMC5266481; doi:10.1016/j.chembiol.2016.11.009)
Supplement: Document S1. Supplemental Experimental Procedures, Figures S1–S7, Tables S1 and S2, and Supplemental References [file mmc1.pdf]

Cell Chemical Biology, Volume *24*

Supplemental Information

CBP/p300 Bromodomains Regulate Amyloid-like Protein Aggregation upon Aberrant Lysine Acetylation

Heidi Olzscha, Oleg Fedorov, Benedikt M. Kessler, Stefan Knapp, and Nicholas B. La Thangue

Supplemental Information
Supplemental Figures

Fig. S1

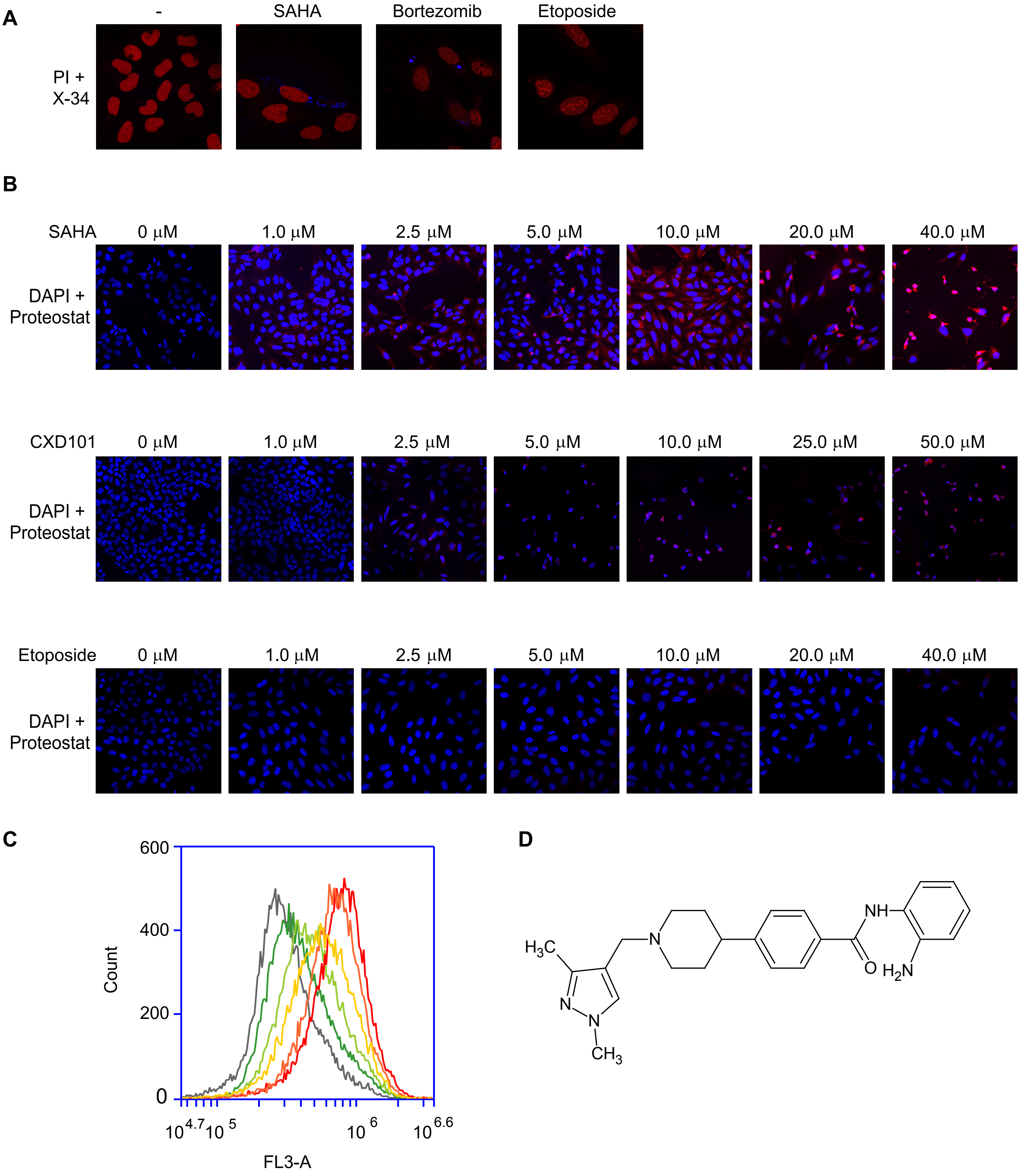

Figure S1 (related to Figure 1)
Detection of aggregates upon dose-escalation treatment with different HDIs.
(A) U2OS cells were treated with SAHA, bortezomib or etoposide for 24 h with concentrations to their respective IC50s. Cells were then incubated with the dye X-34 (blue) specifically recognizing amyloid-like structures in living systems. They were then fixed, permeabilized and propidium iodide (red) was used as a nuclear counterstain.
(B) Staining of U2OS cells upon treatment with increasing concentrations of the HDIs SAHA or CXD101 or the topoisomerase inhibitor etoposide with the dye Proteostat. Cells were treated with indicated concentrations of the compound for 24 h, fixed, permeabilized, and stained with the Proteostat dye in a 1:2000 dilution from the supplier's stock solution (red). They were counterstained with DAPI (blue) and analysed by confocal microscopy, in order to visualize the increasing staining upon SAHA, CXD101 or etoposide treatment.
(C) U2OS cells were treated with either DMSO (grey), or increasing concentrations of SAHA ranging from 1.0 µM (dark green), 2.5 µM (light green), 5.0 µM (yellow), 10.0 µM (orange) and 20.0 µM (red), for 24 h, fixed and permeabilized. Then the cells were stained with Proteostat and analyzed in the FL-3 channel by flow cytometry.
(D) Structure of CXD101.

Fig. S2

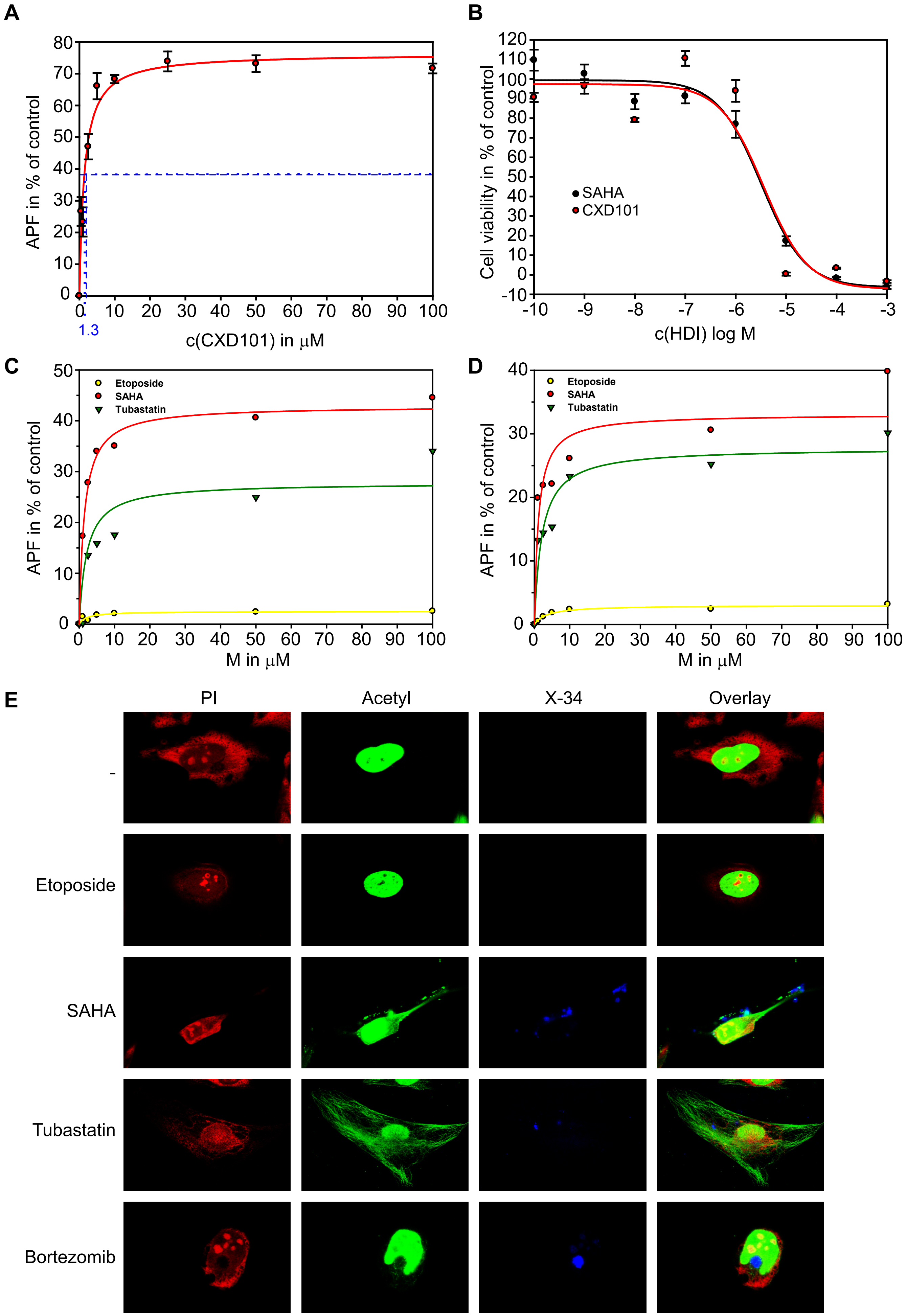
A
APF in % of control
c(CXD101) in µM
1.3
B
Cell viability in % of control
c(HDI) log M
SAHA
CXD101
C
APF in % of control
M in µM
Etoposide
SAHA
Tubastatin
D
APF in % of control
M in µM
Etoposide
SAHA
Tubastatin
E
PI
Acetyl
X-34
Overlay
-
Etoposide
SAHA
Tubastatin
Bortezomib

Figure S2 (related to Figure 1 and 2)

Co-localisation of acetylated proteins with X-34-positive aggregates upon SAHA treatment and quantitation of aggregate formation in different cell lines.

(A) U2OS cells were treated with CXD101, inducing the formation of Proteostat-positive aggregates, as shown for instance in Figure S1B. Cells were measured by flow cytometry in the FL3 channel and the aggregation propensity factor (APF) was calculated. APFs were averaged from three independent biological replicates and APFmax ½ was calculated, indicating the half maximal concentration, aggregation occurs.

(B) Cell viabilities of U2OS cells after treatment with different concentrations of the pan-HDIs SAHA or CXD101 to determine the IC50s and working range for the inhibitors.

Cell viability was determined after 24 h by means of MTT assay. Cell viability was normalized to cells treated DMSO only. Experiments were performed in three biological replicates, each in triplicates; error bars represent the standard deviation (SD). SAHA treated cells are shown in black, whereas values for CXD101 are shown in red.

(C) RIVA cells were treated with SAHA, tubastatin or etoposide inducing the formation of Proteostat-positive aggregates, as shown for instance in Figure S1B. Cells were measured *via* flow cytometry in the FL3 channel and the aggregation propensity factor (APF) was calculated. Shown is one representative experiment.

(D) HBL-1 cells were treated with SAHA, tubastatin or etoposide inducing the formation of Proteostat-positive aggregates, as shown for instance in Figure S1B. Cells were measured *via* flow cytometry in the FL3 channel and the aggregation propensity factor (APF) was calculated. Shown is one representative experiment.

(E) U2OS cells were treated with the indicated drugs and stained with X-34 (blue). After fixation and permeabilization, cells were stained with an antibody against acetylated lysines (green). PI staining was applied to visualize nuclei or nucleated bodies after treatment with the respective drugs (red). Confocal images showing in case of SAHA treatment co-localization of acetylated proteins with X-34 positive aggregates mainly in the cytoplasm.

Fig. S3

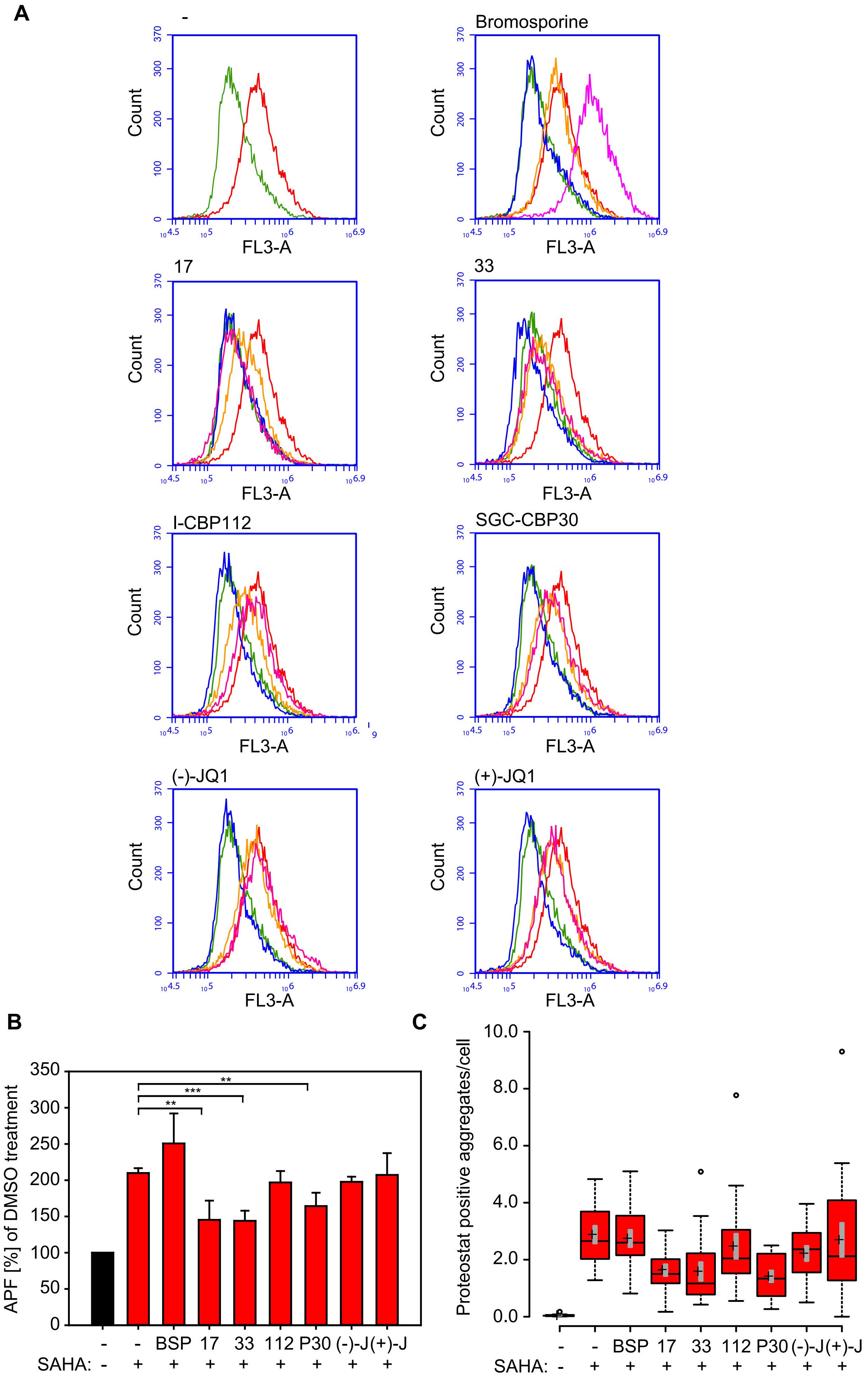

A
-
Bromosporine
17
33
I-CBP112
SGC-CBP30
(-)-JQ1
(+)-JQ1
Count
FL3-A
B
APF [%] of DMSO treatment
**
***
**
- - BSP 17 33 112 P30 (-)-J (+)-J
SAHA: - + + + + + + + +
C
Proteostat positive aggregates/cell
- - BSP 17 33 112 P30 (-)-J (+)-J
SAHA: - + + + + + + + +

Figure S3 (related to Figure 2)

Quantitative assessment of the reduction of aggregates upon BDI treatment

(A) FACS profiles of U2OS cells after co-treatment with SAHA and BDIs stained to detect aggregates. USOS cells were treated with the respective drug combinations, fixed, permeabilized and stained with Proteostat in order to quantitate changes in the aggregation propensity upon treatment. Cells were measured in the FL3 channel and cells displayed in the in FSC/SSC channels (data not shown) were gated to exclude debris, but not to exclude apoptotic cells. In green, cells are represented which were treated with DMSO only (-), red represents SAHA treated cells, blue shows cells which were treated with 2.5 μM of the respective BDI alone, yellow represents cells which are treated with SAHA and 1.0 μM of the respective BDI and in pink cells were treated with SAHA and 2.5 μM of the BDI.

(B) U2OS cells were treated with SAHA (5 μM) and BDIs (2.5 μM) as indicated and 24 h later fixed, permeabilized and stained with Proteostat. At least 20,000 cells were measured by FACS in the FL-3 channel and the mean fluorescence recorded which was normalized to the DMSO (-) only treated control (black), SAHA treated samples are depicted in (red). The data were derived from four independent biological replicates, each performed in three technical replicates and the aggregation propensity factor (APF) was calculated. Level of statistical significance is indicated (** $P \leq 0.001$, *** $P \leq 0.001$) and error bars represent the standard deviation (SD). BSP, bromosporine; 17, compound 17; 33, compound 33; 112, I-CBP112; P30, SGC-CBP30; (-)-J, (-)-JQ1; (+)-J, (+)-JQ1.

(C) U2OS cells were treated with SAHA (5 μM) and BDIs (2.5 μM), then fixed, permeabilized and stained with Proteostat and DAPI as a nuclear counterstain. The number of Proteostat-positive aggregates was then quantified with an IN Cell Analyzer 1000. The data were derived from three independent experiments, for each condition at least 1500 cells were analysed in technical replicates. DMSO-only treatment (-) are depicted in grey, SAHA treated cells in red. Sample means are indicated by crosses, confidence intervals (90%) by grey bars. BSP, bromosporine; 17, compound 17; 33, compound 33; 112, I-CBP112; P30, SGC-CBP30; (-)-J, (-)-JQ1; (+)-J, (+)-JQ1.

Fig. S4

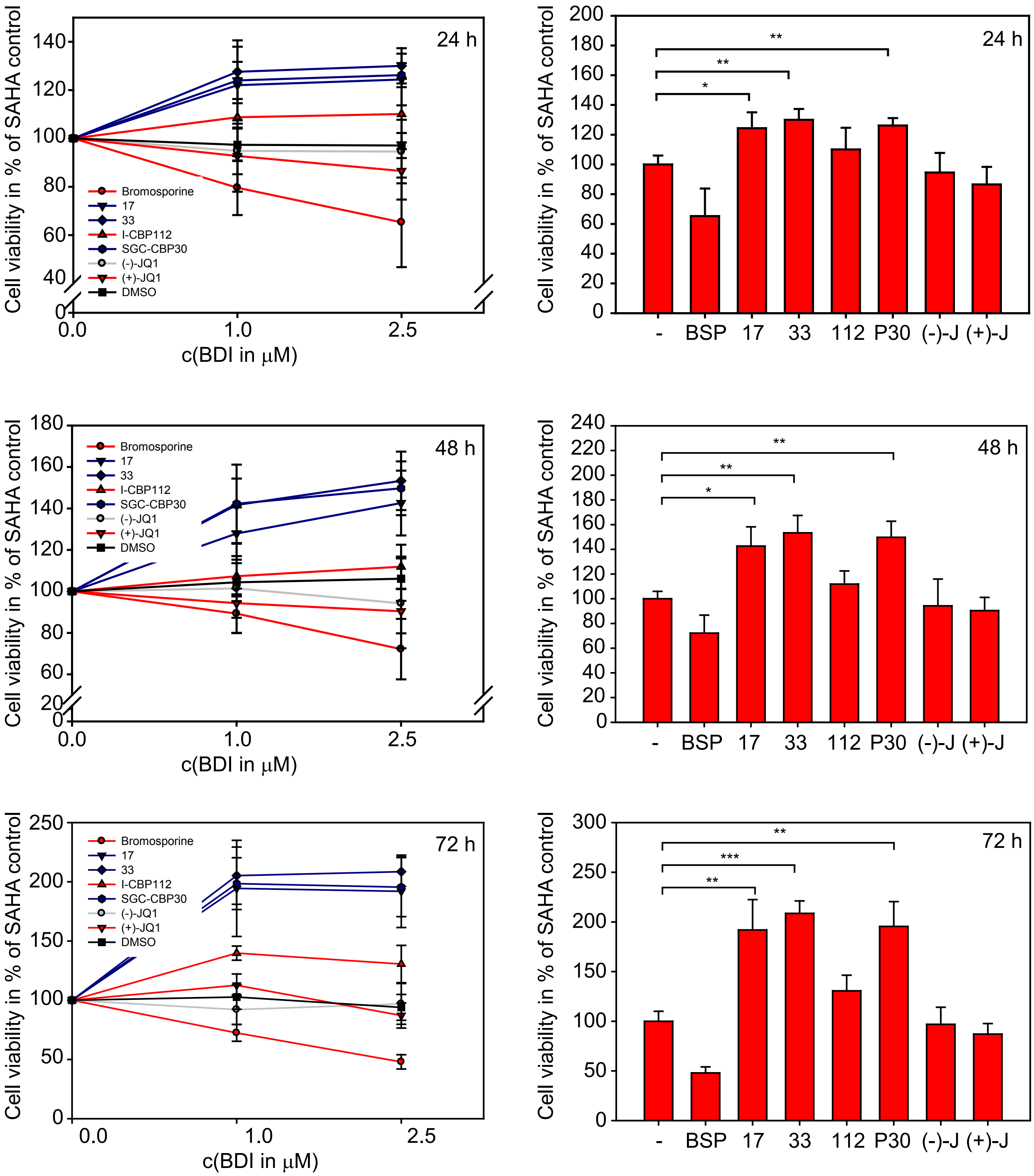

24 h
48 h
72 h
Cell viability in % of SAHA control
c(BDI in μM)
0.0
1.0
2.5
Bromosporine
17
33
I-CBP112
SGC-CBP30
(-)-JQ1
(+)-JQ1
DMSO
-
BSP
17
33
112
P30
(-)-J
(+)-J
*
**
***

Figure S4 (related to Figure 2)

Cell viabilities of U2OS cells after co-treatment with SAHA in combination with different BDIs and after different time points.

BDIs were applied 2 h before cells were treated with the pan-HDAC inhibitor, and then cell viability was determined after the indicated times by means of MTT assay. Cell viability was normalized to cells treated with SAHA only (indexed in the left panel). Experiments were performed in at least three biological replicates, each in triplicates. Values of the BDIs which are able to rescue the toxic effects of SAHA treated cells are shown in blue in the left panels, whereas values for SAHA or BDIs which do not change the cytotoxicity or increase it, are shown in red, the inactive compound (-)-JQ1 is shown in grey and DMSO treated cells are shown in black. Level of statistical significance is indicated (* $P \leq 0.05$, ** $P \leq 0.01$, *** $P \leq 0.001$), error bars represent the standard deviation (SD). BSP, bromosporine; 17, compound 17; 33, compound 33; 112, I-CBP112; P30, SGC-CBP30; (-)-J, (-)-JQ1; (+)-J, (+)-JQ1.

Fig. S5

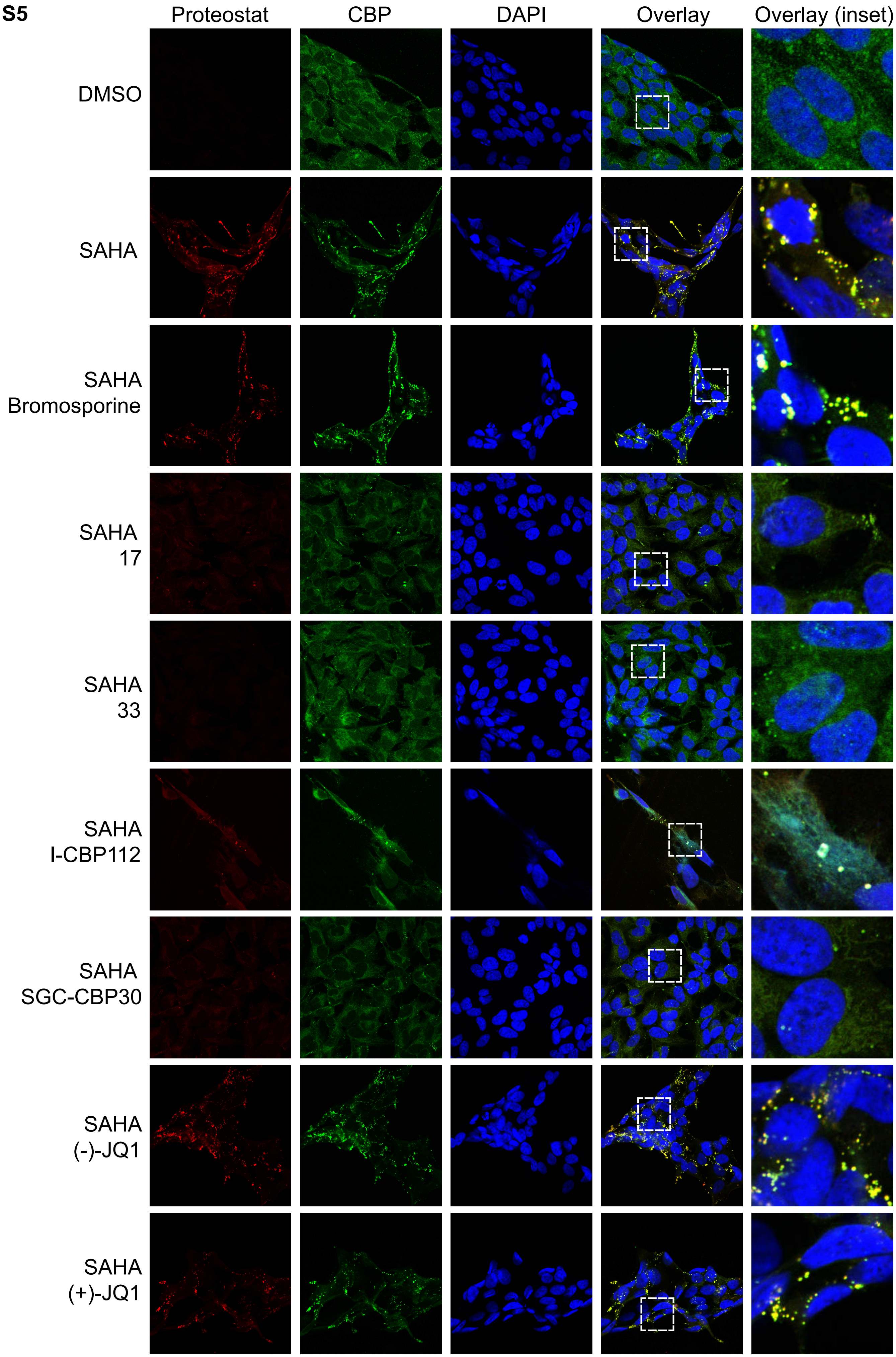

Proteostat
CBP
DAPI
Overlay
Overlay (inset)
DMSO
SAHA
SAHA Bromosporine
SAHA 17
SAHA 33
SAHA I-CBP112
SAHA SGC-CBP30
SAHA (-)-JQ1
SAHA (+)-JQ1

Figure S5 (related to Figure 4)
SAHA induced aggregates are reduced in neuroblastoma cells upon treatment with CBP/p300 specific BDIs.
SH-SY5Y cells were treated with SAHA (5 μM) or DMSO (-) and in parallel with 2.5 μM of the indicated BDIs. Cells were fixed, permeabilized and stained with an antibody against endogenous CBP (green), Proteostat (red) and DAPI (blue) was used as a nuclear counterstain. Panels on the right represent the enlarged area shown by a dashed square on the left side, and aggregates which co-localize with endogenous CBP appear as yellow in the images.

Fig. S6

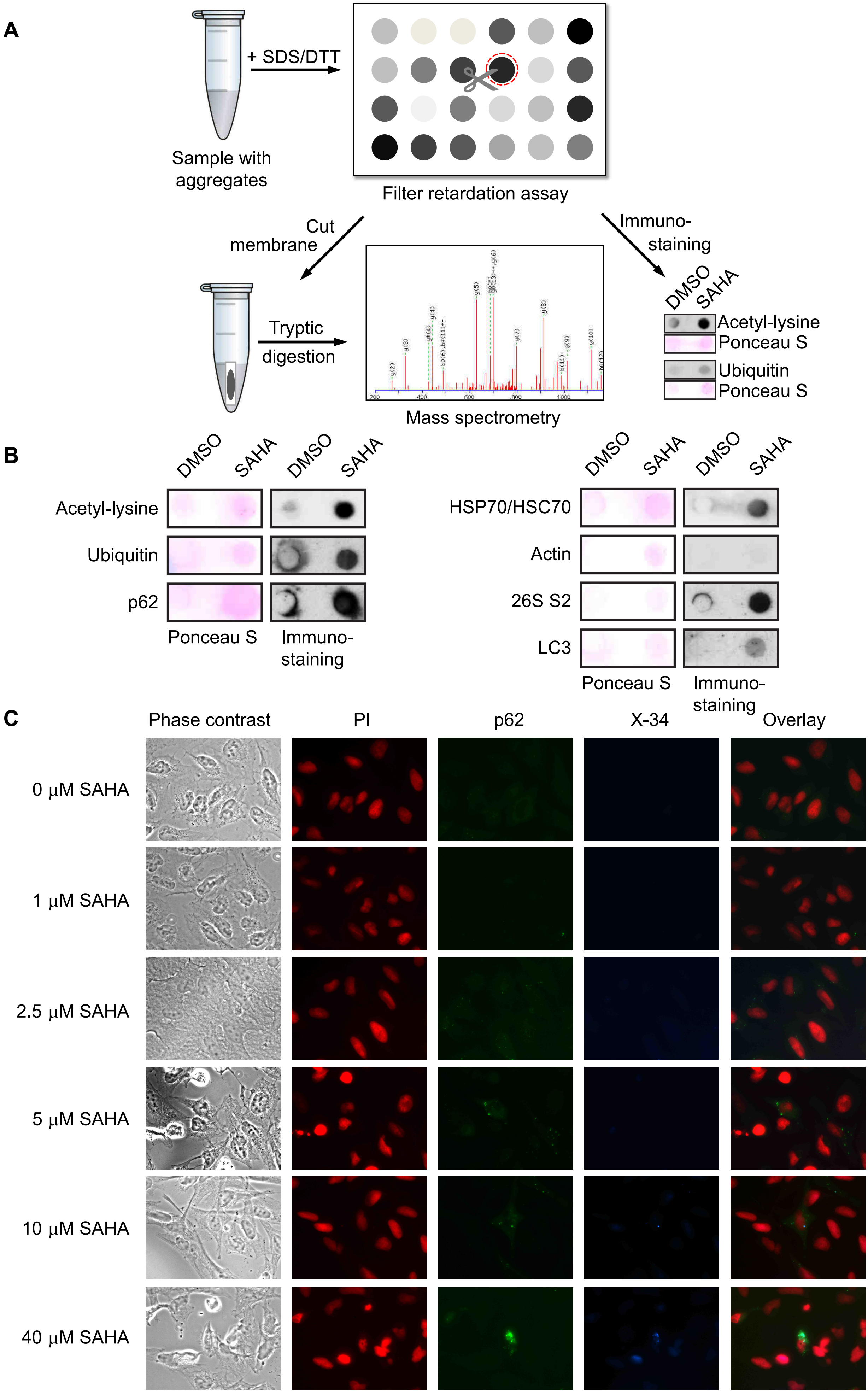
A
+ SDS/DTT
Sample with aggregates
Filter retardation assay
Cut membrane
Immuno-staining
Tryptic digestion
Mass spectrometry
DMSO
SAHA
Acetyl-lysine
Ponceau S
Ubiquitin
Ponceau S
B
DMSO
SAHA
DMSO
SAHA
Acetyl-lysine
Ubiquitin
p62
Ponceau S
Immuno-staining
HSP70/HSC70
Actin
26S S2
LC3
Ponceau S
Immuno-staining
C
Phase contrast
PI
p62
X-34
Overlay
0 μM SAHA
1 μM SAHA
2.5 μM SAHA
5 μM SAHA
10 μM SAHA
40 μM SAHA

Figure S6 (related to Figure 6)

Scheme of the filter retardation assay to enrich aggregated proteins and co-localisation of p62 with X-34 positive aggregates.

(A) U2OS cells containing aggregated proteins were subjected to lysis buffer with detergent and DTT. Samples were filtered on a cellulose acetate membrane and aggregates with a size of more than 200 nm retained. The membrane was then washed and subjected to mass spectrometry in order to identify the retained proteins. In parallel, aggregates were analysed on the membrane by immunostaining, and the total protein amounts were visualized by Ponceau S staining.

(B) U2OS cells were either treated with DMSO or SAHA (5 µM) for 24 h, then harvested, membranes and DNA removed and the remaining aggregate fraction treated with detergent. The material captured in the filter retardation assay was visualized by Ponceau S staining or immunostaining with the indicated antibodies.

(C) Co-localization of p62 with X-34 positive aggregates. p62 was detected upon treatment of U2OS cells with increasing concentrations of SAHA (green), in parallel cells were stained with the dye X-34 (blue), specifically recognizing amyloid-like aggregates. PI staining was employed to visualize the nuclei (red).

Fig. S7

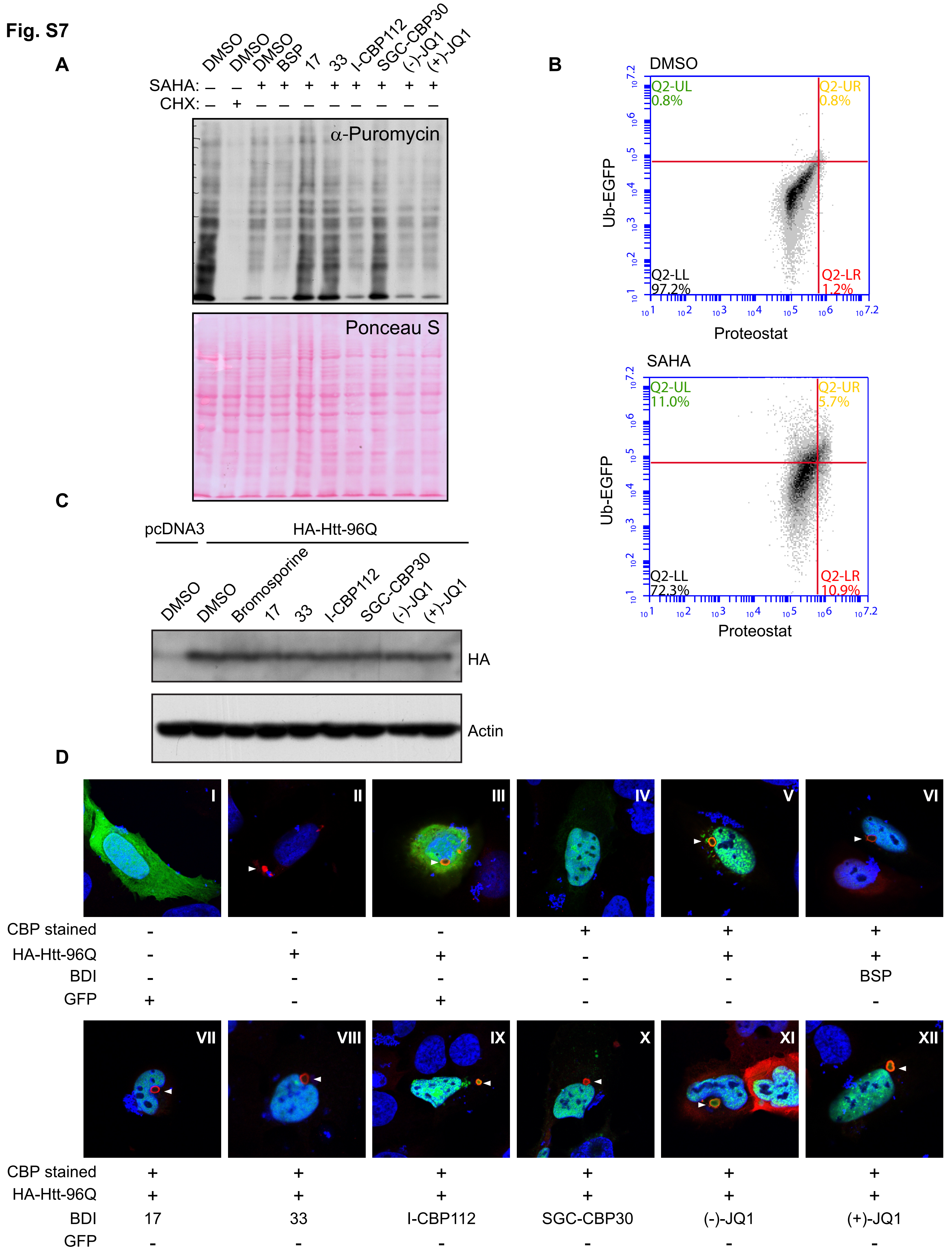
A
DMSO
DMSO
DMSO
BSP
17
33
I-CBP112
SGC-CBP30
(-)-JQ1
(+)-JQ1
SAHA:
CHX:
α-Puromycin
Ponceau S
B
DMSO
Q2-UL 0.8%
Q2-UR 0.8%
Q2-LL 97.2%
Q2-LR 1.2%
Ub-EGFP
Proteostat
SAHA
Q2-UL 11.0%
Q2-UR 5.7%
Q2-LL 72.3%
Q2-LR 10.9%
Ub-EGFP
Proteostat
C
pcDNA3
HA-Htt-96Q
DMSO
DMSO
Bromosporine
17
33
I-CBP112
SGC-CBP30
(-)-JQ1
(+)-JQ1
HA
Actin
D
I
II
III
IV
V
VI
CBP stained
HA-Htt-96Q
BDI
GFP
BSP
VII
VIII
IX
X
XI
XII
CBP stained
HA-Htt-96Q
BDI
GFP
17
33
I-CBP112
SGC-CBP30
(-)-JQ1
(+)-JQ1

Figure S7 (related to Figure 6 and Figure 7)

Effects of BDIs on protein biosynthesis and degradation and on the pathologically elongated form of huntingtin.

(A) Cells were treated with SAHA (5 μM) and the indicated BDIs and then the effect on mRNA translation was visualized by incorporation of puromycin in the nascent polypeptide chain and subsequent immunoblotting with a monoclonal antibody against puromycin in polypeptide chains. For comparison, Ponceau S serves as a loading control. A representative anti-puromycin immunoblot is shown and the corresponding Ponceau S staining.

(B) FACS analysis of HEK293T cells stably expressing Ub-EGFP. Green fluorescence (FL1 channel) of the Ub-EGFP is plotted on the y-axis, red fluorescence of Proteostat staining (FL3 channel) on the x-axis. Quadrants were chosen according to the DMSO control which is set to the lower left quadrant. Upper right quadrants are used in Figures 6D and 6E.

(C) U2OS cell expressing the HA-Htt-96Q exon 1 were treated with the different BDIs. Immunoblotting shows that the level of Htt is not altered. β-actin serves as loading control.

(D) U2OS cells overexpressing HA-Htt-96Q exon1 and CBP-GFP or GFP, with or without different BDIs. Cell were incubated with an HA-antibody against HA-Htt-96Q (shown in red), whereas CBP-GFP or GFP is shown in green and nuclei were counterstained with DAPI (blue). Overlaid channels are displayed, where co-localization appears in yellow or orange. In (I), cells were transfected with GFP only, in (II) with HA-Htt-96Q only, in (III) with both constructs. Cells were transfected with CBP-GFP in (IV) and with both HA-Htt-96Q and CBP-GFP in (V), co-localized Htt-96Q and CBP-GFP (orange) are indicated by an arrow. In (I) to (V), cells were treated with DMSO, whereas ascending from (VI) to (XII), cells were treated with the different BDIs in the following order: Bromosporine (BSP), 17, 33, I-CBP112, SGC-CBP30, (-)-JQ1 and (+)-JQ1. Loss of co-localization between HA-Htt-96Q and CBP-GFP in the panels VII (BDI 17), VIII (BDI 33) and X (BDI SGC-CBP30) is shown in red and indicated by an arrow.

Table S1, related to Experimental Procedures, Bromodomain Inhibitors

Overview of the bromodomain inhibitors (BDIs) used in this study. Listed are names, chemical structures, specificities and respective references.

BDI	Structure	Specificity
Bromosporine	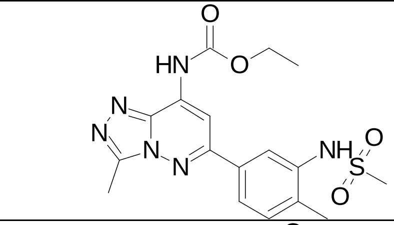	Bromosporine - inhibitor of several bromodomain proteins
17	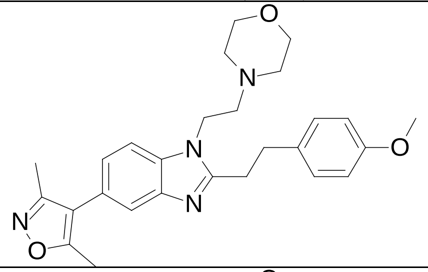	Potent and selective inhibitor of CBP/p300 bromodomain.<br>Kd < 100 nM<br>(Hay, et al., 2014)
33	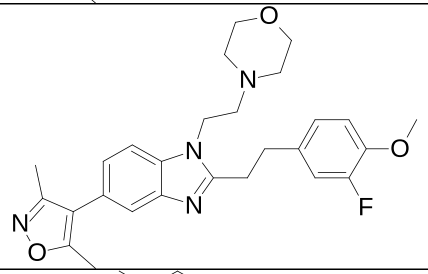	Analogue of 17, more potent, Kd = 50 nM and more selective over BRD4 (1μM)<br>(Hay, et al., 2014)
I-CBP112	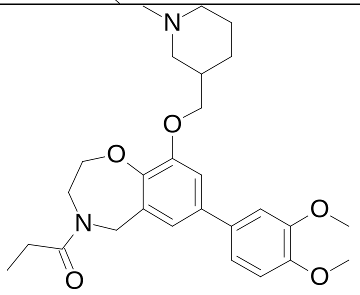	Selective bromodomain inhibitor with dissociation constant<br>Kd = 151 ± 6 nM and 167 ± 8 nM for CBP and p300, respectively<br>(Picaud, et al., 2015)
SGC-CBP30	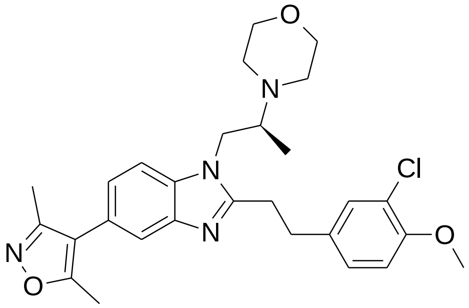	Chemical probe for CPB/p300,<br>Kd = 21/30 nM, 40 fold selectivity over BRD4<br>(Hay, et al., 2014)

Table S1 (continued), related to Experimental Procedures, Bromodomain Inhibitors

Overview of the bromodomain inhibitors (BDIs) used in this study. Listed are names, chemical structures, specificities and respective references.

BDI	Structure	Specificity
(-)-JQ1	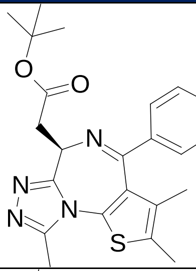	Negative control for (+)-JQ (Filippakopoulos, et al., 2010)
(+)-JQ1	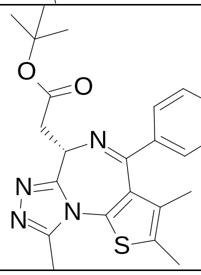	Chemical probe for BET bromodomains, Kd < 100 nM (Filippakopoulos, et al., 2010)

Filippakopoulos, P., Qi, J., Picaud, S., Shen, Y., Smith, W.B., Fedorov, O., Morse, E.M., Keates, T., Hickman, T.T., Felletar, I., et al. (2010). Selective inhibition of BET bromodomains. Nature 468, 1067-1073.

Hay, D.A., Fedorov, O., Martin, S., Singleton, D.C., Tallant, C., Wells, C., Picaud, S., Philpott, M., Monteiro, O.P., Rogers, C.M., et al. (2014). Discovery and optimization of small-molecule ligands for the CBP/p300 bromodomains. Journal of the American Chemical Society 136, 9308-9319.

Picaud, S., Fedorov, O., Thanasopoulou, A., Leonards, K., Jones, K., Meier, J., Olzscha, H., Monteiro, O., Martin, S., Philpott, M., et al. (2015). Generation of a Selective Small Molecule Inhibitor of the CBP/p300 Bromodomain for Leukemia Therapy. Cancer Res 75, 5106-5119.

Table S2, related to Figure 6

Proteins detected in mass spectrometry upon filter retardation. Gene symbols, potential acetylation and percentages of lysine and arginine residues are displayed as well as molecular weight and PI.

Accession no.	Gene Symbol	PI	Mass	Acetylation databases Phosida, UniProt, MaxQB, CPLM and PhosphoSitePlus)	Lysine residues in %	Arginine residues in %
A6NHL2	TUBAL3	5.68	50675	Yes	3.6	6.1
A6NJY1	SLC9B1P1	7.66	31093	No	5	2.1
A6NMY6	ANXA2P2	6.49	38806	Yes	9.4	5.9
A6NNZ2	TBB8L	4.75	50168	No	2.7	5
O00567	NOP56	9.24	66408	Yes	10.9	5.2
O14979	HNRPDL	9.59	46580	Yes	6.7	7.9
O15049	N4BP3	8.33	60889	No	4.4	10.3
O43175	PHGDH	6.29	57356	Yes	4.9	4.1
O60282	KIF5C	5.86	109997	Yes	9.9	5.7
O94925	GLS	7.85	74269	Yes	6.1	4.9
O95573	ACSL3	8.65	81338	Yes	8.2	3.8
P00387	CYB5R3	7.18	34441	Yes	5.3	6
P04181	OAT	6.57	48846	Yes	6.4	4.8
P04350	TUBB4A	4.78	50010	Yes	3.2	5
P04792	HSPB1	5.98	22826	Yes	3.4	7.8
P05198	EIF2S1	5.02	36374	Yes	6.7	7.9
P07814	EPRS	7.02	172080	Yes	9.8	3.9
P07900	HSP90AA1	4.94	85006	Yes	10.9	4.1
P07902	GALT	6.49	43621	No	3.2	6.9
P08107	HSPA1A	5.48	70294	Yes	7.8	4.8
P08134	RHOC	6.2	22334	No	7.8	7.8
P08559	PDHA1	8.35	43952	Yes	5.9	7.2
P08865	RPSA	4.79	32947	Yes	3.7	5.1
P09382	LGALS1	5.34	15048	Yes	5.9	3.7
P0CG38	POTEI	5.83	122858	Yes	8	4.8
P11142	HSPA8	5.37	71082	Yes	8.4	4.3
P11177	PDHB	6.2	39550	Yes	5.3	5
P11586	MTHFD1	6.89	102180	Yes	6.6	4.6
P13639	EEF2	6.41	96246	Yes	7.6	5.1
P13804	ETFA	8.62	35400	Yes	7.8	3.3
P14618	PKM	7.96	58470	Yes	7	5.8
P14625	HSP90B1	4.76	92696	Yes	9.7	4.7
P15880	RPS2	10.25	31590	Yes	8.2	8.2
P17066	HSPA6	5.81	71440	Yes	6.1	7
P18621	RPL17	10.18	21611	Yes	13.6	8.2
P21333	FLNA	5.7	283301	Yes	6	3.5
P21817	RYR1	5.18	570517	Yes	4.5	5.8
P22087	FBL	10.18	33877	Yes	5.9	9

Table S2 (continued), related to Figure 6

Proteins detected in mass spectrometry upon filter retardation. Gene symbols, potential acetylation and percentages of lysine and arginine residues are displayed as well as molecular weight and PI.

Accession no.	Gene Symbol	PI	Mass	Acetylation databases Phosida, UniProt, MaxQB, CPLM and PhosphoSitePlus)	Lysine residues in %	Arginine residues in %
P22492	HIST1H1T	11.71	22006	Yes	19.3	6.8
P23246	SFPQ	9.45	76216	Yes	4.7	8.5
P23396	RPS3	9.68	26842	Yes	8.2	7.4
P23526	AHCY	5.92	48255	Yes	6.7	3.9
P25705	ATP5A1	9.16	59828	Yes	5.4	6.9
P31415	CASQ1	4.03	45132	Yes	6.1	2.3
P31943	HNRNPH1	5.89	49484	Yes	3.6	6.9
P32119	PRDX2	5.66	22049	Yes	7.1	5.1
P36578	RPL4	11.07	47953	Yes	13.3	9.6
P40939	HADHA	9.16	83688	Yes	9.3	4.3
P42704	LRPPRC	5.81	159003	Yes	7.3	4.8
P46776	RPL27A	11	16665	Yes	12.8	8.8
P46777	RPL5	9.73	34569	Yes	11.8	8.1
P49327	FASN	6.01	275877	Yes	3.4	5.7
P49411	TUFM	7.26	49852	Yes	6.6	6
P49736	MCM2	5.34	102516	Yes	4.8	7.6
P50914	RPL14	10.94	23531	Yes	17.2	6
P50990	CCT8	5.42	60153	Yes	7.5	3.3
P52701	MSH6	6.5	154514	Yes	7.4	6.3
P54136	RARS	6.26	76129	Yes	8.3	5.3
P57721	PCBP3	8.22	39725	Yes	5.1	3.8
P61160	ACTR2	6.3	45017	Yes	6.3	6.3
P61978	HNRNPK	5.39	51230	Yes	4.8	7.8
P62241	RPS8	10.32	24475	Yes	14.9	11.1
P62244	RPS15A	10.14	14944	Yes	8.5	8.5
P62249	RPS16	10.21	16549	Yes	11	9.6
P62701	RPS4X	10.16	29807	Yes	10.3	8.4
P62753	RPS6	10.85	28834	Yes	14.5	12.9
P62826	RAN	7.01	24579	Yes	8.3	4.2
P62829	RPL23	10.51	14970	Yes	10.7	7.9
P62913	RPL11	9.64	20468	Yes	9	9.6
P62937	PPIA	7.68	18229	Yes	8.5	3.6
P63244	GNB2L1	7.6	35511	Yes	5.4	4.4
P68363	TUBA1B	4.94	50804	Yes	4.2	4.9
P68371	TUBB4B	4.79	50255	Yes	3.4	5.2
P78527	PRKDC	6.75	473749	Yes	6.7	5.5
Q00839	HNRNPU	5.76	91269	Yes	8.5	11.9

Table S2 (continued), related to Figure 6

Proteins detected in mass spectrometry upon filter retardation. Gene symbols, potential acetylation and percentages of lysine and arginine residues are displayed as well as molecular weight and PI.

Accession no.	Gene Symbol	PI	Mass	Acetylation databases Phosida, UniProt, MaxQB, CPLM and PhosphoSitePlus)	Lysine residues in %	Arginine residues in %
Q02543	RPL18A	10.73	21034	Yes	9.7	3.9
Q05639	EEF1A2	9.11	50780	Yes	9.9	3.9
Q06210	GFPT1	6.66	79555	Yes	6.4	6.3
Q06830	PRDX1	8.27	22324	Yes	9.5	3
Q08211	DHX9	6.41	142181	Yes	5.1	5.8
Q12931	TRAP1	8.3	80345	Yes	6.3	7.5
Q13268	DHRS2	9.21	27764	Yes	4.6	6.1
Q13439	GOLGA4	5.33	261892	No	12.7	4
Q13509	TUBB3	4.83	50856	Yes	3.6	4.9
Q13885	TUBB2A	4.78	50274	Yes	3.4	4.9
Q14103	HNRNPD	7.62	38581	Yes	9	3.9
Q14204	DYNC1H 1	6.01	534809	Yes	6.3	6.2
Q14315	FLNC	5.65	293407	Yes	5.3	4.3
Q14568	HSP90AA 2	4.57	39454	Yes	10.8	2
Q15149	PLEC	5.74	533462	Yes	5.3	9.7
Q15233	NONO	9.01	54311	Yes	5.7	9.8
Q15365	PCBP1	6.66	37987	Yes	3.9	4.5
Q16695	HIST3H3	11.13	15613	Yes	9.6	13.2
Q3ZCM7	TUBB8	4.79	50257	No	3.2	5.2
Q562R1	ACTBL2	5.39	42318	Yes	4.8	5.3
Q58FF6	HSP90AB 4P	4.65	58855	No	10.1	2.4
Q58FF7	HSP90AB 3P	4.71	68624	No	9.5	3
Q58FF8	HSP90AB 2P	4.79	44492	Yes	12.3	2.6
Q58FG1	HSP90AA 4P	5.07	47796	No	9.1	4.3
Q5JNZ5	RPS26P1 1	10.55	13336	No	10.4	12.2
Q5VTE0	EEF1A1P 5	9.15	50495	Yes	10.4	3.7
Q5VX52	SPATA1	8.54	50504	No	10.5	5
Q6NVV1	RPL13AP 3	10.76	12184	No	20.6	9.8
Q70CQ4	USP31	9.35	148328	No	5.8	6.7
Q71DI3	HIST2H3 A	11.27	15436	Yes	9.6	13.2

Table S2 (continued), related to Figure 6

Proteins detected in mass spectrometry upon filter retardation. Gene symbols, potential acetylation and percentages of lysine and arginine residues are displayed as well as molecular weight and PI.

Accession no.	Gene Symbol	PI	Mass	Acetylation databases Phosida, UniProt, MaxQB, CPLM and PhosphoSitePlus)	Lysine residues in %	Arginine residues in %
Q71DI3	HIST2H3A	11.27	15436	Yes	9.6	13.2
Q7KZ85	SUPT6H	4.81	200203	Yes	6.8	6.3
Q8IYB3	SRRM1	11.84	102331	Yes	11.3	15.9
Q8N257	HIST3H2BB	10.31	13900	Yes	15.9	6.3
Q8WZ42	TTN	6.02	3842904	Yes	8.6	4.8
Q92538	GBF1	5.48	208367	Yes	5	5
Q92841	DDX17	8.53	80906	Yes	4.3	8.1
Q96AE4	FUBP1	7.18	67690	Yes	4.3	4.5
Q96KN1	FAM84B	5.34	34681	No	3.5	7.1
Q99714	HSD17B10	7.66	27134	Yes	4.2	4.2
Q9BUF5	TUBB6	4.77	50281	Yes	3.1	4.9
Q9BYX7	POTEKP	5.91	42331	No	5.9	4.5
Q9NQC3	RTN4	4.43	130250	Yes	7	2.1
Q9NR30	DDX21	9.32	87804	Yes	11.6	5.5
Q9NY65	TUBA8	4.94	50746	Yes	4.2	4.7
Q9UQ35	SRRM2	12.05	300179	Yes	4.7	17
Q9Y3R0	GRIP1	6.03	123202	Yes	5.7	5.2
**Mean**					**7.5**	**6.1**

Supplemental experimental procedures

Antibodies

Anti-Ub (P4D1) (Santa Cruz, sc-8017), anti-acetyl-histone H3 (Millipore, 06-599), anti-LC3B (Cell Signaling, 2775), anti-p53 (Santa Cruz, sc-126), anti-acetylated tubulin (Sigma, T7451), anti-proteasome 26S S2 (Abcam, ab197054), anti-p62/SQSTM1 (Santa Cruz, sc-28359), anti-HSP90α/β (F-8) (Santa Cruz, sc-13119), anti-acetylated-lysine (Cell Signaling, 9441), anti-HSP70/HSC70 (W27) (Santa Cruz, sc-24), anti-α-tubulin (Santa Cruz, sc-8035), anti-p300 (C-20) (Santa Cruz, sc-585), anti-CBP (C-1) (Santa Cruz, sc-7300), anti-GFP (Roche, 11814460001), anti-puromycin (clone 12D10) (Millipore, MABE343), anti-HA.11 (Clone 16B12) (Covance, MMS-101P), anti-Flag (Sigma, F3165), anti-mouse-Alexa Fluor®488 (Invitrogen, R37114), anti-rabbit-Alexa Fluor®488 (Invitrogen, A-21206), anti-mouse-Alexa Fluor®594 (Invitrogen, A-21203), anti-rabbit-Alexa® Fluor 594 (Invitrogen, A-21207), anti-mouse-HRP (Millipore, 12-349), anti-rabbit-HRP (Millipore, 12-348).

Cell viability and measurement of apoptosis

The overall cell viability was determined by means of MTT assay. Cells treated with inhibitors and/or transfected in a 96-well plate in 100 μl medium were incubated with MTT (3-(4,5-dimethylthiazol-2-yl)-2,5-diphenyltetrazolium bromide) tetrazolium solution (Sigma, M2128) with a final concentration of 0.5 mg/ml. Cells were incubated for exactly 120 min, and the reaction of the conversion of the MTT to purple formazan crystals was stopped by adding 100 μl of solubilizing solution (20 % SDS, 50 % dimethylformamide (DMF)). Cells and crystals were solubilized in this solution overnight and the quantity of formazan directly proportional to the number of viable cells was measured by recording changes in absorbance at 570 nm using a plate reading spectrophotometer (Tecan), as modified by (Mosmann, 1983). SubG1 phase obtained after cell cycle analysis in flow cytometry (see flow cytometry) correlated with the number of dying cells, whereby it could not be distinguished between apoptosis, necrosis or other types of cell death. To differentiate between the different events, cleaved caspase-3 or cleaved PARP served as apoptotic markers (see also section immunoblotting).

RNA interference

U2OS cells were transfected with CBP esiRNA or p300 esiRNA (Sigma, EHU155151-20UG and EHU075681-20UG) or control GFP siRNA, mixed in OptiMEM and Oligofectamine (Invitrogen, 12252011) as described in the manufacturer's instructions. The mixture was added to 1 x 10^5 cells in 1 ml medium (2 ml total) for 6 h, seeded into a 6-well plate and incubated for 4 days. 24 h before the lysis for immunoblotting or fixation for microscopy or FACS, cells were treated with SAHA (5 μM) and the respective BDIs (2.5 μM).

Cell lysis and immunoblotting

Cells were split and 1 day later they were seeded in 10 cm dishes, for each sample 1.5 million of cells. The cells were then treated 24 h later with the respective compounds and/or were transfected or the protein was induced by doxycycline treatment. The cells were harvested and washed twice with ice-cold PBS and lysed with ice-cold modified RIPA buffer (50 mM Tris pH 7.4, 150 mM NaCI, 1 mM EDTA, 0.25 % sodium deoxycholate, 1.0 % NP-40, protease inhibitors 10 μg/ml Aprotinin (Sigma), 10 μg/ml Leupeptin (Sigma), 10 μg/ml Pepstatin (Roche), 50 μg/ml AEBSF (Sigma), 1 μM MG132 (Calbiochem), 10 μM SAHA, 20 μM NEM (Sigma)) at 4ºC for 20 min. Protein concentration was determined according to Bradford. 40 μg of cell lysate was diluted in 2x Laemmli buffer and an SDS-PAGE was performed, followed by immunoblot. Equal loading was monitored *via* Ponceau S staining. The membranes were blocked for 1 h in 5 % dry milk in TBS and then decorated with the primary antibodies over-night. On the next day, the membranes were washed 3 x with TBS and then decorated with a secondary antibody conjugated to HRP for 2 h. After washing 2 x with TBS-T and once with TBS, the membranes were incubated with ECL and the signal detected with films.

Filter retardation assay

In order to isolate aggregated species of different unknown proteins under native conditions, at least 24 million differently treated (DMSO and SAHA) cells were lysed. They were washed twice in PBS and were lysed in 1400 μl of a mild lysis buffer containing 0.1 % NP40, 100 mM NaCl, 20 mM Tris, 10 mM $MgCl_2$, pH 7.4, protease inhibitors (10 μg/ml Aprotinin (Sigma), 10 μg/ml Leupeptin (Sigma), 10 μg/ml Pepstatin (Roche), 50 μg/ml AEBSF (Sigma), 1 μM MG132 (Calbiochem, 474790)), 10 μM SAHA, 20 μM NEM (Sigma, E3876) and 80 units benzonase® nuclease (Millipore, 70746) for 1 h. The lysate was centrifuged with only 2000 g for 5 min, in order to remove the cell debris, but to keep the aggregates soluble (Scherzinger, et al., 1997). The supernatant with the

aggregates was kept on ice and the protein concentration was determined according to Bradford. Detergent was titrated to optimize the filter retardation process, but not to destroy the aggregates, and for this reason the final concentration was brought to 1.3 % SDS and 33 mM DTT. Similar protein amounts were then filtered through a cellulose acetate membrane with a pore size of 0.2 μm (Sterlitech) in a dot blot apparatus. Membranes were washed 3 times with 400 μl PBS and air dried. They were then stained with Ponceau S to visualize the retained protein amount. The membranes were then either treated as described in immunoblotting to detect individual proteins amongst the retained proteins with antibodies, or 10 dots from the membrane with the retained proteins on it were cut and pooled and further processed to analyse the proteins *via* mass spectrometry.

Preparation for mass spectrometry (MS) analysis

The digestion of peptides from a membrane was modified after (Fernandez and Mische, 2001), since cellulose acetate was used in this case. Briefly, membrane dots with retained proteins after performing the filter retardation assay were cut, 10 of them were air dried, cut into 1 mm large pieces, pooled and put in a 1.5 ml pre-lubricated microcentrifuge tube (Fisher Scientific). Usually, empty membranes were run as negative control to determine any protein adherent to the empty membrane and exclude them from the results. 100 μl digestion buffer (50 mM NH_4HCO_3 in ddH_2O, pH 8.0) was added and the samples were sonicated for 5 min and vortexed for a minute. The samples were then incubated at room temperature for 30 min with rigorous shaking at 1400 rpm, and then again sonicated for 5 min. and vortexed for a minute. 5 μl DTT was then added to a final concentration of 10 mM, and samples were incubated at room temperature for 1 h. Then 20 μl chloracetamide was added to a final concentration of 55 mM and incubated for 1 h. 20 μl DTT was added to a final concentration of 10 mM and the samples were again incubated for 1 h at room temperature with shaking. The samples were then filled up with 775 μl digestion buffer, and 25 μl of a 0.2 mg/ml stock solution of trypsin (Promega, V5111) in digestion solution was added, and the samples were digested in solution overnight at 37 ºC. They were then for 5 min sonicated, and vortexed for 1 min. This was repeated twice, the samples were then spun at 16000 g and the supernatant given in a new pre-lubricated tube. To the remaining cellulose acetate fragments, 100 μl digestion buffer was added, and the samples were sonicated for 5 min. and vortexed for 1 min., this was repeated once, then spun and the supernatant combined with the previous one. Peptides were then purified by the Sep-Pak C18 light (Waters) purification columns according to the manufacturer's recommendation. Samples were washed with washing solution (98 % MilliQ-H_2O, 2 % CH_3CN, 0.1 % formic acid), and eluted with elution solution (35 % MilliQ-H_2O, 65 % CH_3CN, 0.1 % formic acid).

Analysis of MS data

Analysis of the proteins was carried out as described previously (New, et al., 2013). In brief, samples were subjected to nLC-MS/MS analysis, and peptides and proteins identified by Mascot (v2.3.01 CBRG Cluster) *via* automated database searching of all MS/MS spectra against the UniProt SwissProt swissprot/uniprot_sprot.v2012.07.13 homo sapiens database (20,306 sequences). The data were analysed with the following search parameters: Type of search: MS/MS Ion Search; Enzyme: Trypsin; Fixed modifications: Carbamidomethyl (C); Variable modifications: Oxidation (M), Deamidated (NQ), Acetyl (K), GlyGly (K); Mass values: Monoisotopic; Protein mass: Unrestricted; Peptide mass tolerance: ± 1.8 Da (# 13C = 1); Fragment mass tolerance: ± 0.5 Da; Max missed cleavages: 3; Instrument type: ESI-TRAP. After removing known contaminants from the list of detected proteins, only those proteins, for which at least two significant peptide matches were identified, were accepted. The UniProt accession numbers were searched against the STRING database version 9.0 (Jensen, et al., 2009) for protein-protein interactions. Only interactions between the proteins belonging to the dataset were selected. STRING defines a metric called confidence score to define interaction confidence; all interactions for the dataset were chosen which had a confidence score ≥ 0.4. The clustering was performed *via* the MCL option which accepts a parameter called "inflation" and was set to 2. Further analysis and the determination of GO enrichments were carried out with Cytoscape 2.8.3 (Shannon, et al., 2003) and plugin BiNGO 2.44 (Maere, et al., 2005). The following parameters were applied for the BiNGO search: Hypergeometric test; Benjamini and Hochberg false discovery rate correction; significance level: 0.05; GO biological process; organism: Homo sapiens. The following databases were searched against acetylation (K) and acetylation sites: MaxQB, UniProt, Phosida, Compendium of Protein Lysine Modifications (CPLM) and PhosphoSitePlus.

Analysis of protein synthesis by puromycin labelling

In order to measure translation by non-radioactive means, puromycin labelling during protein synthesis was applied. The experiments were modified after (Schmidt, et al., 2009). Cells were grown in 10 cm dishes, treated

accordingly to the experiment, trypsinized and harvested at 37 ºC, and the cell number were adjusted to 1 million in a microtube with 1 ml pre-warmed medium. In control samples, cycloheximide was brought to a final concentration of 100 μM and cells were incubated at 37 ºC for 30 min, and the tubes were gently mixed every 10 min. Puromycin was added to a final concentration of 10 μg/ml and the cells were incubated for further 10 min at 37 ºC with gently shaking every 5 min. The cells were then spun for 2 min at 500 g in a pre-warmed (37 ºC) centrifuge and once quickly washed with pre-warmed PBS. They were then immediately frozen to – 80 ºC, and after 15 min they were lysed as described in RIPA buffer. With the lysates, immunoblotting was performed, and an anti-puromycin antibody (clone 12D10, Millipore) was used to detect puromycinylated protein species. The signal was quantitated with ImageJ and the Fiji plugin (Schneider, et al., 2012) and normalized to the total protein amount, visualized by ponceau S staining.

Fluorescence imaging

Fluorescence imaging of the adherent or semi-adherent cells was performed using an Olympus BX60 inverted microscope or a Zeiss LSM 780 confocal laser scanning microscope equipped with a with the 32 channel GaAsP detector. Images were acquired using the Leica HCX PL APO 63X oil immersion objective with numerical aperture of 1.4 and a resolution of 1024 x 1024. Virtual zoom for most confocal image was set to 1.00 or 2.5 and scales are indicated by a white bar and image acquisition and analysis was performed using Leica TCS Analysis software. Images were recorded in czi image format, processed with the blue ZEN Zeiss software and converted into Tiff files. X-34, DAPI and Thioflavin S stained samples were recorded in the blue fluorescent channel or, if the confocal microscope was used, with an excitation wavelength of a diode laser (405 nm, CW/pulsed) 30 mW, Proteostat stained samples were excitated with red fluorescent light or a HeNe-laser (543 nm) 1 mW, and structures stained with an antibody coupled to Alexa 594 were excitated with red fluorescent light or a HeNe-laser (594 nm) 2 mW. GFP or proteins which were detected by an Alexa 488 labeled antibody were excited with an Ar-laser (488 nm) 35 mW. The filters were set to the respective emission wavelength of the different dyes, which is 500 nm for X-34 and Thioflavin S determined by a λ-scan in the Zeiss LSM 780 microscope, and 620 nm for Proteostat.

Fluorescence Recovery After Photobleaching (FRAP)

U2OS cells were grown in 8-well μ-slide chambers (ibidi) and were treated with the respective pan-HDAC inhibitor and different BDIs, or transfected with HA-Htt-96Q. After two days in case of the pan-HDAC treatment or one day in case of Htt transfection, cells were stained with the dye X-34 for 45 minutes, which can penetrate through cell membranes and selectively stain amyloid-like aggregates. Cells were then washed for 15 minutes with pre-warmed medium. The FRAP and imaging system consisted of a Zeiss LSM 780 AxioObserver microscope equipped with a high-numerical-aperture PlanApochromat 63x/1.40 Oil DIC M27 oil immersion objective (Zeiss). Cells in the chambers were placed in an incubator capable of maintaining temperature (37 ºC), humidity and CO_2 (5 %) atmosphere. FRAP imaging was carried out with a 405 nm laser and with a PMT detector set to detect fluorescence between 416 nm and 585 nm. A region of interest within the aggregate was empirically selected for bleaching. A time lapse series was taken to record X-34 fluorescence recovery using 0.5 % of the power used for bleaching. Initial scanning prior to bleaching was carried out to record the baseline for correcting the bleaching caused by the detection. 150 frames were recorded; with an image size of 33.74 μm. Zoom was 4.0 and the frame time was 0.97 s. The data were fitted by the ZEN 2012 microscope control software according to a one parameter exponential model to determine the average half-time for recovery for 5-10 cells per treatment in at least 5 independent experiments.

Analysis of aggregates with the IN Cell analyser

To quantify microscopic changes of aggregate number and size, 1500 to 5000 cells were seeded on a 96-well plate and treated accordingly. Cells were fixed, permeabilized and stained as described for immunofluorescence staining, with the difference that one staining was always performed with 5 μg/ml FITC (Sigma, F7250) or 1 μg/ml propidium iodide without prior RNAse A digestion. Nuclear staining was either performed with DAPI or propidium iodide with prior RNAse A treatment. Aggregates were stained either with X-34 or Proteostat as described in the section immunofluorescence staining. Cells were fixed again after all staining procedures for 30 min with 4% paraformaldehyde and then covered with 200 μl PBS. Intracellular structures were measured with an IN Cell analyser 1000 instrument (GE Healthcare) according to the manufacturer's instructions. Briefly, cells were analysed with 3 different wavelengths and filters in order to record blue, green and red fluorescence with a 20 x objective. 12 fields in each well were acquired in the centre of the well, so that in total per experiment and condition at least 1400 cells were analysed. Exposure times for X-34 were 1000 ms, for Proteostat 2000 ms, for the FITC counterstain and DAPI/PI staining 500 ms.

The raw images were then automatically analysed by the Multi Target Analysis Module for the IN Cell Analyzer 1000 (GE Healthcare), with the following parameters: Wave 1: Object nuclei, top hat, minimum area 200 μm^2, sensitivity 100; Wave 2: Object cells, multiscale top hat, minimum area 500 μm^2, sensitivity 25; Wave 3: Object organelles, multiscale top hat, granule size minimum 0.5 μm, granule size maximum 2 μm for HDAC inhibitor induced aggregates, 10 μm for Htt aggregates, process parameters 3 pixels, sensitivity 80, detect inclusions in cells; Reference in wave3: pseudo.

Flow cytometry

Aggregation analysis

Cells were harvested 24 h or 48 h after different treatments. Cells were washed once with pre-warmed PBS, trypsinized (Lonza), centrifuged at 400 g for 3 min. and washed again with PBS. They were fixed for 30 min. with 4 % paraformaldehyde in PBS, washed once in PBS and permeabilized for 30 min at 4 ºC in 0.5 % Triton. Cells were then washed 2 times in PBS and stained for 30 min with Proteostat in a 1: 7000 dilution in the provided buffer (EnzoLifesciences). Without further washing, cells were then analysed in a BD Accuri C6 FACS machine. At least 20000 events were analysed per condition and final gating. Cells were analysed in the FL3 channel, after gating from the FSC/SSC plot in order to exclude cell debris. In case the reporter protein Ub-EGFP was analysed in parallel, an additional gate were set to ensure the analysis in the FL1 channel. Compensation were then set correcting the FL1 channel for 7 % for the FL3 channel, and to correct the FL3 channel, compensation for the FL1 channel was set to 10 %. Log fluorescence intensities were plotted against each other and quadrants were set in order to determine the different degrees of UPS inhibition and aggregation. The mean FL1 and FL3 intensities in total or according to the set quadrants were used. The raw cytometry data were analysed using CFlowPlus (BD Bioscience). Flow cytometry data of aggregation in FL3 channel were analysed by comparison of mean fluorescence *via* calculation of a term referred to as the Aggregation Propensity Factor (APF), as defined below. APF = 100 x ($MFI_{treated}$ – $MRI_{control}$)/$MFI_{treated}$), wherein $MFI_{treated}$ and $MFI_{control}$ are the mean fluorescence intensity values from control and treated samples. The UPS inhibition factor (UIF) was calculated accordingly.

Cell cycle analysis

After treatment, cells were harvested using trypsin, washed in phosphate buffered saline in PBS (Oxoid) and fixed in 70 % ethanol in PBS. For analysis, the samples were incubated for 30 min with 100 μg/ml RNAse A (Sigma, R6513) and 1 μg/ml propidium iodide (Sigma, P4864) at 4 ºC. Samples were run on a BD Accuri C6 Flow Cytometer (BD Bioscience) and the analysis was carried out using CFlowPlus (BD Biosciences) software. Virtual gain was set to the G1 peak of DMSO treated cells, and other treatments were set accordingly to this peak, in order to have an alignment for the set the different markers for the cell cycle phases.

Statistical Analysis

Student's t test was used to test for statistical significance of the differences between the different group parameters. p values of less than 0.05 were considered statistically significant and error bars represent standard deviation (SD).

Supplemental References

Fernandez, J., and Mische, S.M. (2001). Enzymatic digestion of proteins on PVDF membranes. Current protocols in protein science / editorial board, John E. Coligan ... [et al.] Chapter 11, Unit 11 12.

Jensen, L.J., Kuhn, M., Stark, M., Chaffron, S., Creevey, C., Muller, J., Doerks, T., Julien, P., Roth, A., Simonovic, M., et al. (2009). STRING 8--a global view on proteins and their functional interactions in 630 organisms. Nucleic acids research 37, D412-416.

Maere, S., Heymans, K., and Kuiper, M. (2005). BiNGO: a Cytoscape plugin to assess overrepresentation of gene ontology categories in biological networks. Bioinformatics 21, 3448-3449.

Mosmann, T. (1983). Rapid colorimetric assay for cellular growth and survival: application to proliferation and cytotoxicity assays. J Immunol Methods 65, 55-63.

New, M., Olzscha, H., Liu, G., Khan, O., Stimson, L., McGouran, J., Kerr, D., Coutts, A., Kessler, B., Middleton, M., et al. (2013). A regulatory circuit that involves HR23B and HDAC6 governs the biological response to HDAC inhibitors. Cell Death Differ 20, 1306-1316.

Scherzinger, E., Lurz, R., Turmaine, M., Mangiarini, L., Hollenbach, B., Hasenbank, R., Bates, G.P., Davies, S.W., Lehrach, H., and Wanker, E.E. (1997). Huntingtin-encoded polyglutamine expansions form amyloid-like protein aggregates in vitro and in vivo. Cell 90, 549-558.
Schmidt, E.K., Clavarino, G., Ceppi, M., and Pierre, P. (2009). SUnSET, a nonradioactive method to monitor protein synthesis. Nature methods 6, 275-277.
Schneider, C.A., Rasband, W.S., and Eliceiri, K.W. (2012). NIH Image to ImageJ: 25 years of image analysis. Nature methods 9, 671-675.
Shannon, P., Markiel, A., Ozier, O., Baliga, N.S., Wang, J.T., Ramage, D., Amin, N., Schwikowski, B., and Ideker, T. (2003). Cytoscape: a software environment for integrated models of biomolecular interaction networks. Genome research 13, 2498-2504.
