# Supplementary material for: CBP/p300 Bromodomains Regulate Amyloid-like Protein Aggregation upon Aberrant Lysine Acetylation
Source: Cell Chem Biol. 2017 Jan 19;24(1):9–23. doi: 10.1016/j.chembiol.2016.11.009 (PMC5266481; doi:10.1016/j.chembiol.2016.11.009)
Supplement: Document S2. Article plus Supplemental Information [file mmc2.pdf]

Article

Cell Chemical Biology

CBP/p300 Bromodomains Regulate Amyloid-like Protein Aggregation upon Aberrant Lysine Acetylation

Graphical Abstract

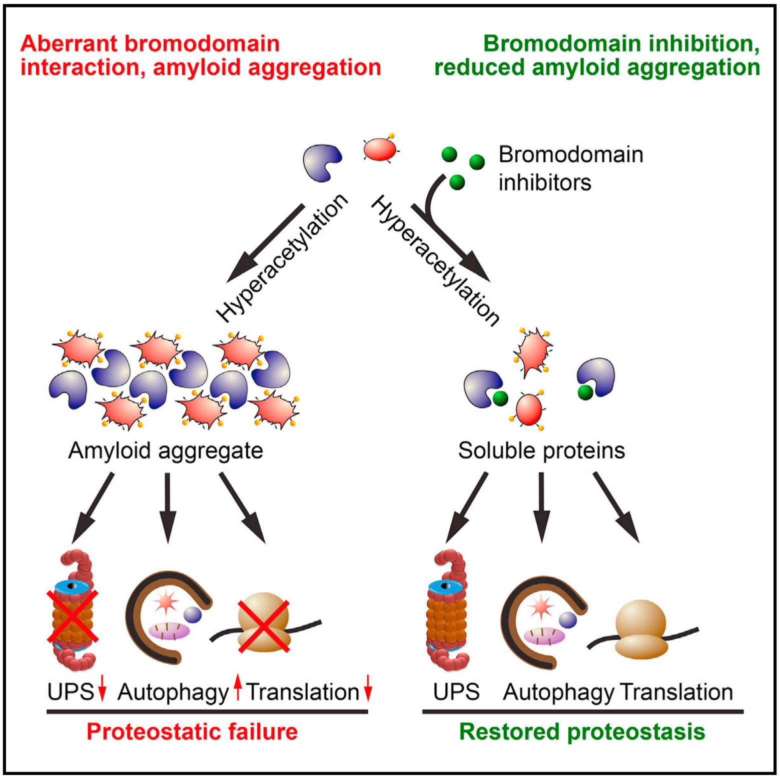

Highlights

- Aberrant acetylation causes amyloid-like aggregation and disturbs proteostasis
- p300/CBP bromodomain proteins are involved in the protein aggregation
- p300/CBP bromodomain inhibitors reverse the cytotoxicity and restore proteostasis
- Bromodomain inhibitors also decrease pathological huntingtin protein aggregation

Authors

Heidi Olzscha, Oleg Fedorov, Benedikt M. Kessler, Stefan Knapp, Nicholas B. La Thangue

Correspondence

nick.lathangue@oncology.ox.ac.uk

In Brief

Olzscha et al. demonstrate that aberrantly acetylated proteins form amyloid-like aggregates in human cells. Inhibition of the p300/CBP bromodomains with small-molecule inhibitors reverses the aggregation-induced cytotoxicity. Aggregates caused by a pathologically elongated huntingtin behave in a similar way.

Olzscha et al., 2017, Cell Chemical Biology *24*, 9–23
January 19, 2017 © 2017 The Authors. Published by Elsevier Ltd.
http://dx.doi.org/10.1016/j.chembiol.2016.11.009

Please cite this article as: Olzscha et al., CBP/p300 Bromodomains Regulate Amyloid-like Protein Aggregation upon Aberrant Lysine Acetylation, Cell Chemical Biology (2017), http://dx.doi.org/10.1016/j.chembiol.2016.11.009

Cell Chemical Biology
Article

CBP/p300 Bromodomains Regulate Amyloid-like Protein Aggregation upon Aberrant Lysine Acetylation

Heidi Olzscha,[1] Oleg Fedorov,[2] Benedikt M. Kessler,[3] Stefan Knapp,[2,4] and Nicholas B. La Thangue[1,5,*]
[1]Department of Oncology
[2]Structural Genomics Consortium
University of Oxford, Old Road Campus Research Building, Old Road Campus, Roosevelt Drive, Oxford OX3 7DQ, UK
[3]Nuffield Department of Medicine, Target Discovery Institute, University of Oxford, Roosevelt Drive, Oxford OX3 7FZ, UK
[4]Present address: Institute of Pharmaceutical Chemistry, Goethe University, Max-von-Laue-Strasse 9, 60439 Frankfurt am Main, Germany
[5]Lead Contact
*Correspondence: nick.lathangue@oncology.ox.ac.uk
http://dx.doi.org/10.1016/j.chembiol.2016.11.009

SUMMARY

Lysine acetylation is becoming increasingly recognized as a general biological principle in cellular homeostasis, and is subject to abnormal control in different human pathologies. Here, we describe a global effect on amyloid-like protein aggregation in human cells that results from aberrant lysine acetylation. Bromodomain reader proteins are involved in the aggregation process and, using chemical biology and gene silencing, we establish that p300/CBP bromodomains are necessary for aggregation to occur. Moreover, protein aggregation disturbs proteostasis by impairing the ubiquitin proteasome system (UPS) and protein translation, resulting in decreased cell viability. p300/CBP bromodomain inhibitors impede aggregation, which coincides with enhanced UPS function and increased cell viability. Aggregation of a pathologically relevant form of huntingtin protein is similarly affected by p300/CBP inhibition. Our results have implications for understanding the molecular basis of protein aggregation, and highlight the possibility of treating amyloid-like pathologies and related protein folding diseases with bromodomain inhibitor-based strategies.

INTRODUCTION

Protein acetylation on lysine side chains is a widespread post-translational modification involved in diverse cellular processes and biological pathways (Choudhary et al., 2014). Histone acetyltransferases (HATs) such as CBP/p300 "write" the acetylation mark, contrasting with histone deacetylases (HDACs) which "erase" the mark (Kouzarides, 1999), while bromodomain-containing proteins including CBP/p300 "read" the mark (Chan and La Thangue, 2001; Filippakopoulos and Knapp, 2014; Zeng and Zhou, 2002). It is generally believed that the equilibrium between modified and unmodified lysine acetylation states has to be tightly regulated to maintain the normal biological role of acetylation in cellular homeostasis. Thus, acetylation is involved in a variety of biological processes, including transcription, protein translation, and degradation as well as cell-cycle control and apoptosis (New et al., 2012). Acetylation can affect the structure of proteins; for example, in the N-terminal region of histone proteins it increases the α-helical content (Wang et al., 2000) and intrinsically unstructured regions (Hansen et al., 2006), perhaps leading to misfolding and aggregation. The cell death caused by aberrant levels of protein acetylation resulting, for example, from HDAC inhibitor (HDI) treatment, is consistent with the biological importance of lysine acetylation (Choudhary et al., 2009; New et al., 2012).

Deregulated acetylation has been suggested to be involved in the pathology of several types of disease, including cancer, inflammation, and metabolic and neurodegenerative diseases (Khan and La Thangue, 2012; Marks et al., 2001; Saha and Pahan, 2006; Zhao et al., 2010). The precise contribution made by acetylation to the disease pathology is, however, much debated. Since lysine acetylation of histones is involved with chromatin control, abnormal gene expression is one level that might be affected (Grunstein, 1997; Marks et al., 2001). Nevertheless, as diverse proteins involved with multiple biological processes are acetylated (Choudhary et al., 2009), acetylation is likely to affect a variety of pathologically relevant mechanisms in addition to chromatin control. One idea that we have considered, given the widespread effect on proteins, is whether lysine acetylation affects protein homeostasis (often referred to as proteostasis), namely the balance between protein synthesis, maturation, and degradation (Balch et al., 2008; Hartl et al., 2011). We have therefore examined whether protein aggregation and, potentially, misfolding, occur in cellular conditions of abnormal lysine acetylation. To reflect the writer-reader interplay established for lysine acetylation (Filippakopoulos and Knapp, 2014), we also addressed whether any influence of lysine acetylation on proteostasis involves bromodomain proteins.

Here, we describe amyloid-like protein aggregates that occur under conditions of aberrant lysine acetylation, dependent on p300/CBP bromodomain proteins for their formation. The presence of amyloid-like aggregates coincides with increased cytotoxicity, and cell viability can be restored upon co-treating cells with small-molecule p300/CBP bromodomain inhibitors (BDIs), which reflect reduced levels of protein aggregates. The p300/CBP bromodomain proteins, together with proteins involved in proteostasis, are present in the aggregates, and p300/CBP

This is an open access article under the CC BY license (http://creativecommons.org/licenses/by/4.0/).

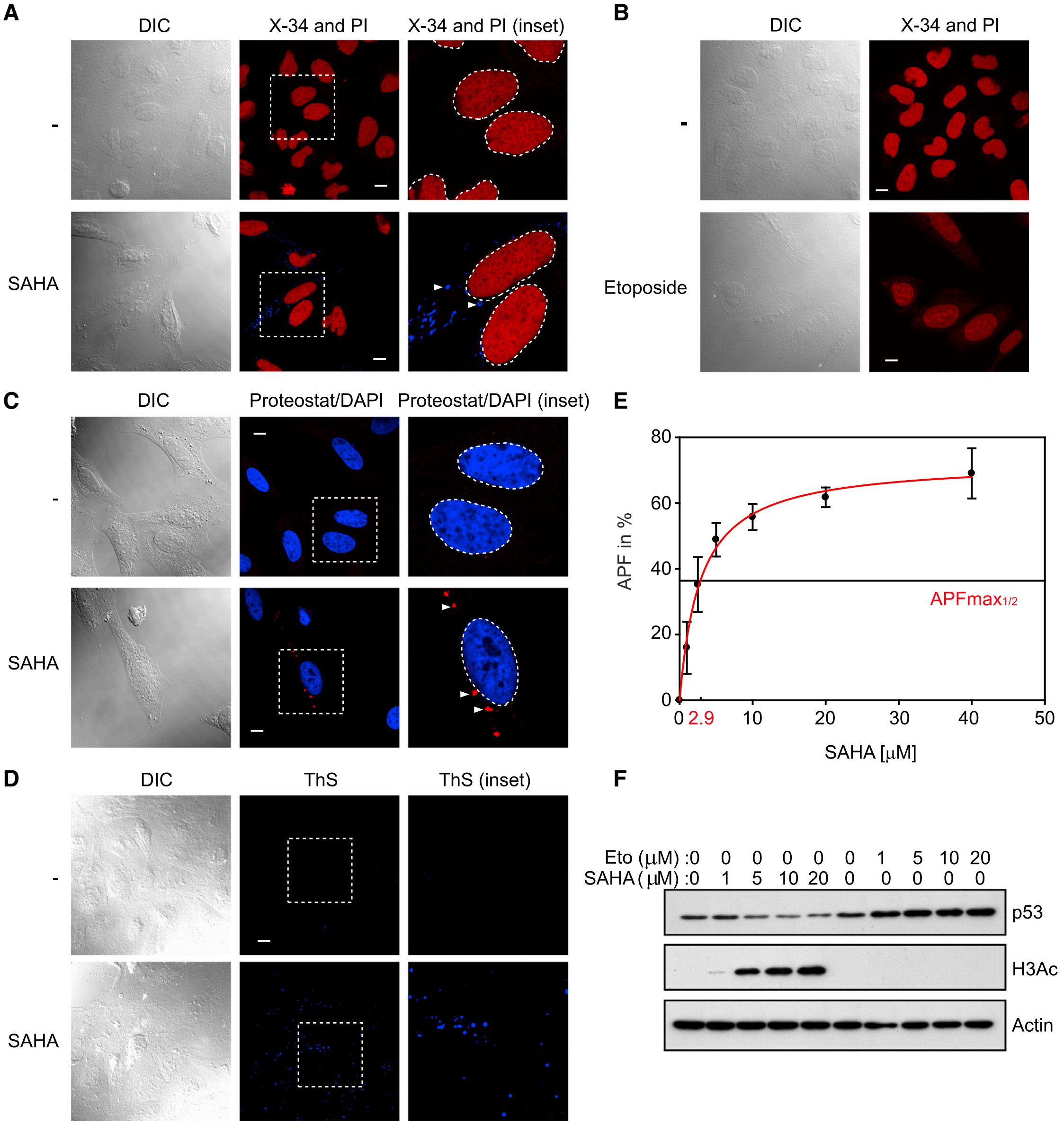

Figure 1. Amyloid-like Protein Aggregates in HDAC Inhibitor-Treated Cells

(A) U2OS cells were treated with either DMSO (control, –) or SAHA (5 μM) for 24 hr and stained with the dye X-34 (blue), which specifically recognizes amyloid-like structures. The cells were then fixed, permeabilized, and counterstained with propidium iodide (PI) to visualize the nuclei (red). Nuclei are indicated with white dashed circles. White arrowheads indicate the position of X-34-positive aggregates. Scale bar represents 10 μm.

(B) U2OS cells were treated with either DMSO (–) or etoposide (5 μM) for 24 hr and stained with X-34 (blue). The cells were then fixed, permeabilized, and counterstained with PI to visualize the nuclei (red). Scale bar represents 10 μm.

(C) U2OS cells were treated with either DMSO as negative control (–) or SAHA (5 μM) for 24 hr, fixed, and permeabilized. The cells were then stained with Proteostat, recognizing aggregated (not necessarily amyloid-like) structures (red), indicated by white arrowheads. Cells were counterstained with DAPI (blue) to visualize their nuclei, indicated here by white circles. Scale bar represents 10 μm.

(D) U2OS cells were treated with either DMSO (–) as negative control or SAHA (5 μM) for 24 hr, fixed, and permeabilized. The cells were then stained with thioflavin S solution, which can specifically stain amyloid structures in in situ specimens (blue). Scale bar represents 10 μm.

(E) U2OS cells were treated with SAHA inducing the formation of Proteostat-positive aggregates, as shown for instance in (C). Cells were measured by flow cytometry in the FL3 channel and the aggregation propensity factor (APF) was calculated. From at least five independent biological replicates APFs were averaged and $APF_{max½}$ was calculated, indicating that at half-maximal concentration aggregation occurs. Error bars denote SD.

(F) Immunoblot corresponding to (A) and (B) measuring acetylated histone H3Ac, demonstrating that the pan-HDAC inhibitor was active and hyperacetylation of proteins appeared. Increasing levels of p53 give a measure for the successful application of etoposide (Eto). β-Actin was used as a loading control.

DIC, differential interference contrast. See also Figures S1 and S2.

CellPress

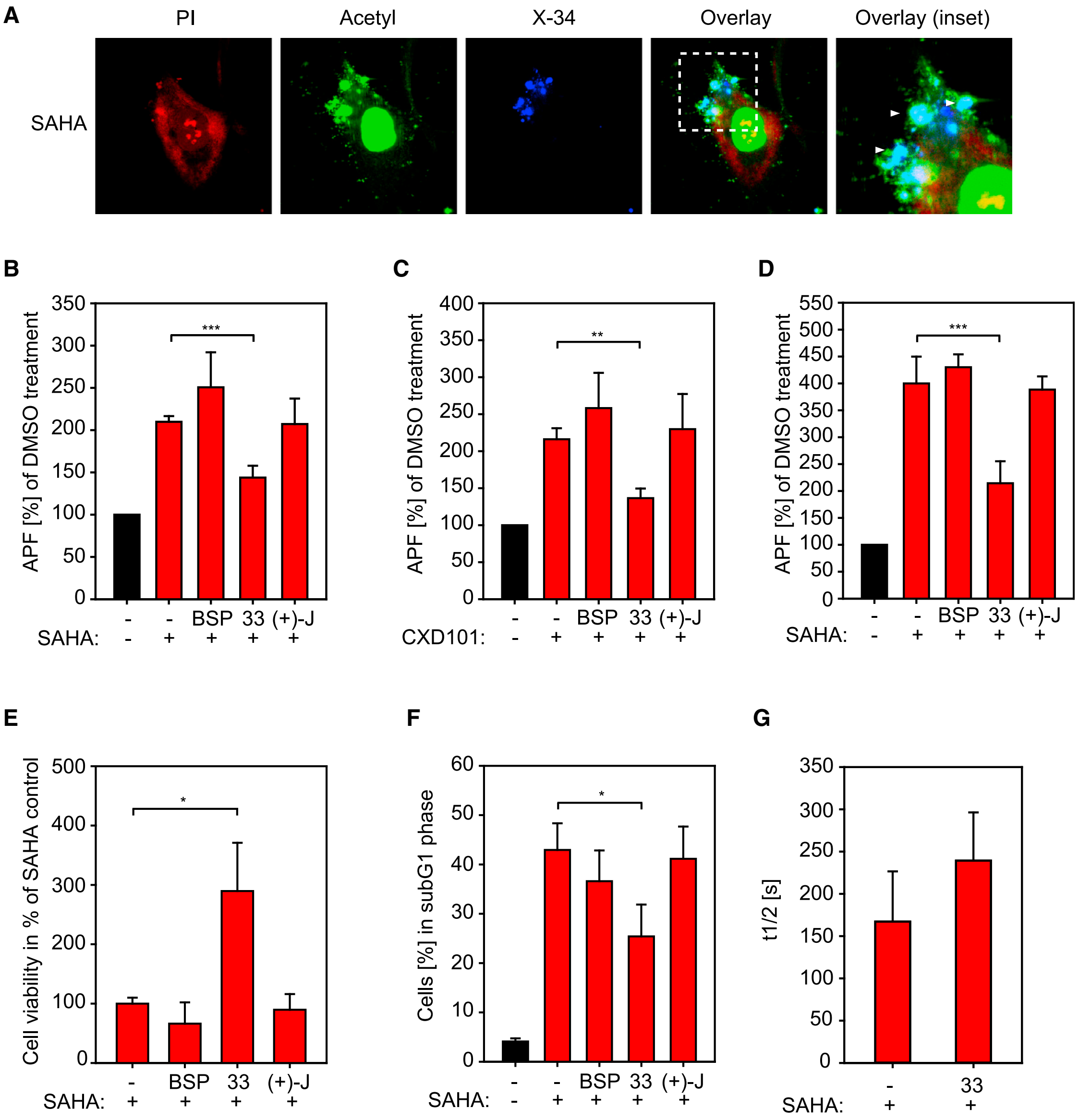

Figure 2. Acetylated Proteins Are Present in the Aggregates, and CBP/p300-Specific BDIs Reduce Aggregation and Cytotoxicity

(A) U2OS cells were treated with the pan-HDAC inhibitor SAHA for 24 hr or DMSO (−). Living cells were stained with X-34, then fixed, permeabilized, and treated with an antibody against acetyl-lysine. Propidium iodide (PI) was used as a nuclear counterstain. Arrows indicate X-34 positive aggregates co-localizing with lysine acetylation (see also Figure S2E).

(B) U2OS cells were treated with SAHA (5 μM) and BDIs (2.5 μM) as indicated and 24 hr later fixed, permeabilized, and stained with Proteostat. At least 20,000 cells were measured by fluorescence-activated cell sorting (FACS) in the FL-3 channel and the mean fluorescence recorded, which was normalized to the DMSO-only (−) treated control (black); SAHA-treated samples are depicted in red. The data were derived from four independent biological replicates, each performed in three technical replicates, and the aggregation propensity factor (APF) was calculated. Level of statistical significance is indicated (***$p \leq 0.001$). Error bars denote SD (see also Figures S3A–S3C). BSP, bromosporine; 33, compound 33; (+)-J, (+)-JQ1.

(C) U2OS cells were treated with CXD101 (5 μM) and BDIs (2.5 μM) as indicated and 24 hr later fixed, permeabilized, and stained with Proteostat. At least 20,000 cells were measured by FACS in the FL-3 channel and the mean fluorescence recorded, which was normalized to the DMSO-only (−) treated control (black); CXD101-treated samples are depicted in red. The data were derived from six independent biological replicates, and the APF was calculated. Level of statistical significance is indicated (**$p \leq 0.01$). Error bars denote SD. BSP, bromosporine; 33, compound 33; (+)-J, (+)-JQ1.

(D) SH-SY5Y cells were treated with SAHA (5 μM) and BDIs (2.5 μM) as indicated and 24 hr later fixed, permeabilized, and stained with Proteostat. At least 20,000 cells were measured by FACS in the FL-3 channel and the mean fluorescence recorded, which was normalized to the DMSO-only (−) treated control (black); SAHA-treated samples are depicted in red. The data were derived from six independent biological replicates, and the APF was calculated. Level of statistical significance is indicated (***$p \leq 0.001$). Error bars denote SD. BSP, bromosporine; 33, compound 33; (+)-J, (+)-JQ1.

(E) U2OS cells were treated with SAHA (5 μM) and co-treated with different BDIs at 2.5 μM. Cell viability was determined by means of the MTT assay 72 hr after co-treatment. The MTT level of SAHA alone was set to 100% and the data represent five independent biological replicates, each with at least three technical

(legend continued on next page)

proteins are necessary for the aggregates to occur. The presence of amyloid-like aggregates impinges on proteostasis, as both protein degradation and protein translation are affected, which similarly can be relieved upon treating cells with p300/CBP-specific BDIs. Furthermore, the amyloid-like protein aggregates formed by a pathologically relevant polyglutamine-expanded Huntington's disease protein are affected by p300/CBP BDIs. Our results suggest a crucial role for protein acetylation and bromodomains as a global regulator of cellular proteostasis, and highlight a novel therapeutic approach based on bromodomain inhibition for treating pathologies dependent upon protein misfolding and aggregation.

RESULTS

Amyloid-like Aggregates in HDAC Inhibitor-Treated Cells

We decided to facilitate high levels of lysine acetylation by treating cells with the pan-HDAC inhibitor SAHA. U2OS cells treated with SAHA at increasing concentration and stained with the amyloid-specific dye X-34 (Styren et al., 2000) exhibited inclusion bodies that were amyloid-like aggregates by virtue of their staining with X-34 (Figure 1A); X-34 was chosen because it offers increased sensitivity and stains viable cells (Link et al., 2001). Aggregates were apparent after 24 hr of treatment, and at increasing doses of SAHA they were even more pronounced with X-34 (Figure S1A). To test whether the aggregates could also be visualized by other dyes that detect amyloid-like structures, we compared X-34 with thioflavin S (Games et al., 1995). Amyloid-like aggregates were also observed upon thioflavin S staining of cells treated with SAHA (Figure 1D). Proteostat, which stains protein aggregates, but which are not necessarily amyloid-like (Shen et al., 2011), similarly stained the aggregates (Figures 1C, 1E, S1B, and S1C). Bortezomib treatment was used as a control as it is established to cause protein aggregation reflecting inhibition of the UPS (Hyun et al., 2003) (Figure S1A). Treating cells with a variety of mechanistically unrelated agents, including etoposide, doxorubicin, and 5-fluorouracil (5-FU), did not reveal any aggregates by X-34 staining (Figures 1B, S1A, and S1B; doxorubicin and 5-FU not shown). We confirmed that SAHA was active under these conditions, through increased acetylation of histone H3 after SAHA, but not etoposide treatment (Figure 1F). Conversely, dose-dependent upregulation of p53 occurred after etoposide treatment, contrasting with SAHA treatment which did not alter p53 levels (Figure 1F). The aggregates were observed with non-hydroxamic acid HDAC inhibitors, such as CXD101, and occurred in other cell lines, including the human DLBCL cell lines RIVA and HBL-1 and the neuroblastoma SH-SY5Y cell line (Figures 2, 4, S1, S2, and S5). In summary, dyes that detect protein aggregates in cells, including amyloid-like structures, identified aggregates that depend upon aberrant lysine acetylation.

Protein Aggregation Decreases upon Treatment with p300/CBP Bromodomain Inhibitors

Given the amyloid-like nature of the aggregates in HDAC inhibitor-treated cells, we were interested to determine whether proteins present in the aggregates were acetylated and, should this be the case, what their identity might be. In cells treated with SAHA the same aggregates co-immunostained with anti-acetyl-lysine antibodies (Figures 2A and S2E). Other cell treatments, including etoposide and bortezomib, did not cause any anti-acetyl-lysine immunostaining (Figure S2E).

We wanted then to address whether the aggregates were a primary event of deregulated lysine acetylation. We reasoned that if they were, then the aggregates should be affected by agents that block lysine acetylation-dependent protein interactions, and considered bromodomains and their inhibitors (BDIs). Thus, we tested whether co-treating cells with HDAC inhibitors and small-molecule BDIs affected the level of protein aggregation, using a collection of BDIs which included the CBP/p300 inhibitors I-CBP112 (Picaud et al., 2015), SGC-CBP30 (Hammitzsch et al., 2015) and its analogs, compounds 17 and 33 (Hay et al., 2014), the BET inhibitor (+)-JQ1 (Filippakopoulos et al., 2010), and the broad-range BDI bromosporine (Theodoulou et al., 2016) (Table S1). Thereafter, cells were stained with Proteostat, and the amount and intensity of staining quantitated by flow cytometry. An increasing dose of SAHA led to a concomitant increase in the number of aggregates, until the level of aggregates plateaued and saturation was achieved (Figures 1E, S1C, and S2), from which APF (aggregation propensity factor) and AC_{50} (50% aggregation concentration) was determined (Figures 1E and S2A). Treatment of cells with each BDI alone did not result in the appearance of aggregates (data not shown). Remarkably, however, in SAHA- or CXD101-treated cells, co-treatment with some of the BDIs decreased the amount and number of aggregates (Figures 2, 4, S3, and S5). Specifically, BDI 17, 33, and SGC-CBP30 specific for the p300/CBP bromodomains (Table S1) reduced the amount of aggregation, whereas BDIs targeting other bromodomain families such as BET proteins had no discernible effect (Figures 2, 4, S3, and S5). We also investigated whether the BDIs had an influence on the stability and building rate of SAHA-induced aggregates using a fluorescence recovery after photobleaching (FRAP) assay. The results derived from cells treated with SAHA with or without the most potent p300/CBP BDI compound 33 (Figure 2) suggested that the half recovery time ($t_{1/2}$) of the material into aggregates increased compared with control treatment (Figure 2G),

replicates. Level of statistical significance is indicated (*p $\leq$ 0.05). Error bars denote SD (see also Figure S4). BSP, bromosporine; 33, compound 33; (+)-J, (+)-JQ1.

(F) U2OS cells were treated either with DMSO (−) or SAHA (5 μM), and different BDIs (2.5 μM). After harvesting and fixation, cells were stained with PI and the different cell-cycle phases were measured by FACS. The percentage of the cells in the sub-G1 phase is shown. Cells treated with DMSO only are depicted in black and SAHA-treated cells in red. Level of statistical significance is indicated (*p $\leq$ 0.05). Error bars denote SD. BSP, bromosporine; 33, compound 33; (+)-J, (+)-JQ1.

(G) U2OS cells treated with pan-HDAC inhibitor and with DMSO (−) or 33 were stained with X-34 and subjected to FRAP analysis. Shown is the $t_{1/2}$ recovery of the bleached aggregates. At least five independent experiments are represented; error bars denote SD. 33, compound 33.

See also Figures S2E, S3, and S4.

Please cite this article as: Olzscha et al., CBP/p300 Bromodomains Regulate Amyloid-like Protein Aggregation upon Aberrant Lysine Acetylation, Cell Chemical Biology (2017), http://dx.doi.org/10.1016/j.chembiol.2016.11.009

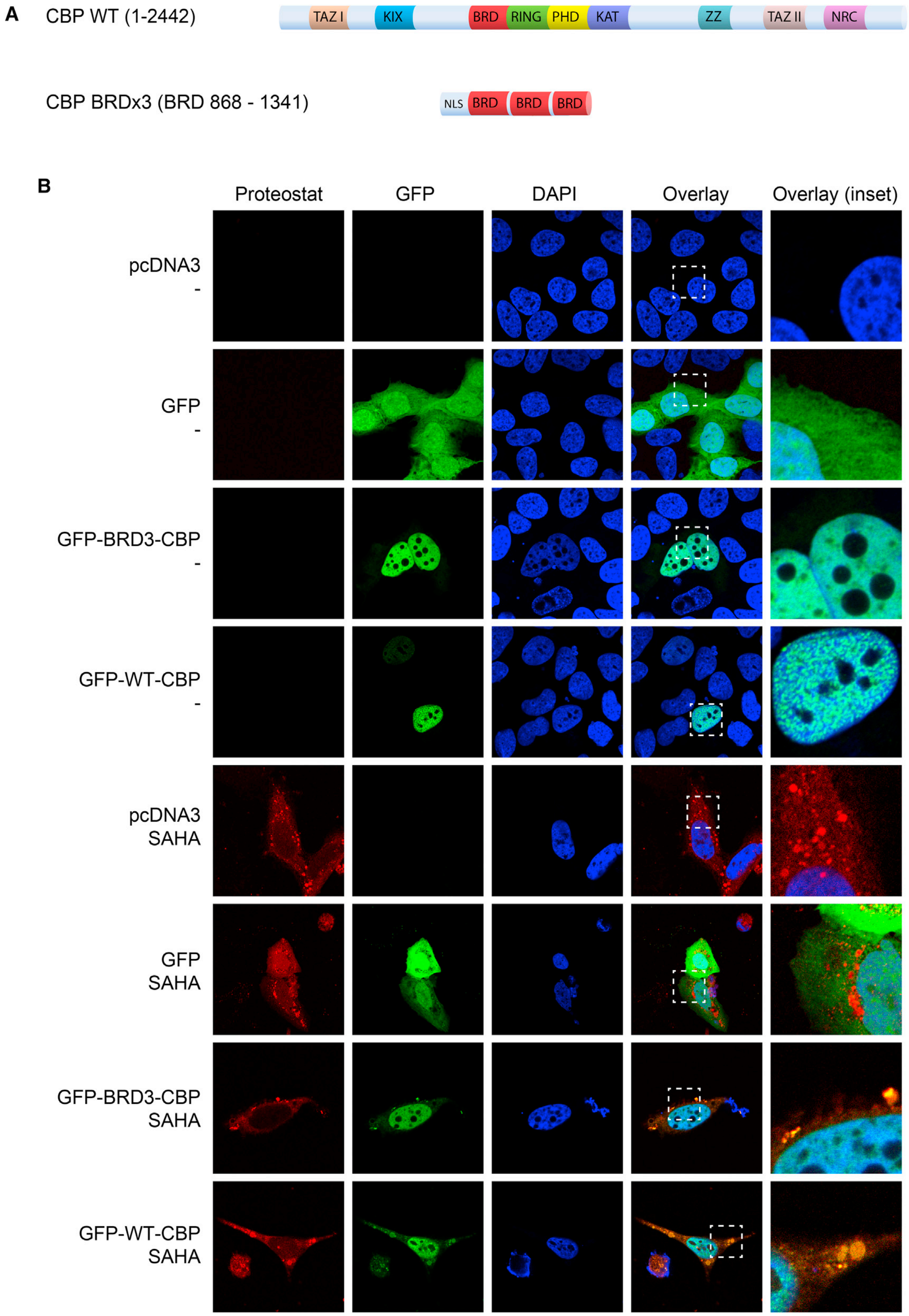

Figure 3. Bromodomains of p300/CBP Are Directly Involved in the Formation of Aggregates upon HDI Treatment

(A) Schematic overview of the CBP full-length protein (1–2,442) and the CBP 3× bromodomain construct (868–1,341 for BRD). In the upper panel, wild-type (WT) CBP (1–2,442) is shown with all its domains; in the lower panel CBP BRD3 (868–1,341 for BRD) is shown, which consists only of three tandem bromodomains with an N-terminal NLS sequence. Both constructs are tagged with GFP.

(legend continued on next page)

implying that formation of the aggregates could be delayed when the p300/CBP bromodomain was inhibited.

We then assessed the effect of bromodomain inhibition on cell viability in cells treated with SAHA. The p300/CBP BDIs that reduced aggregate formation, namely BDI 17, 33, and SGC-CBP30, were similarly able to rescue cells from the cytotoxic effect of SAHA treatment and there was little effect of the other BDIs (Figures 2E and S4). Notably, bromosporine had the opposite effect, since it acted additively with SAHA to reduce cell viability even further (Figures 2E and S4). Cell-cycle analysis indicated that the normal cell-cycle profile was partially restored when SAHA-treated cells were co-treated with the p300/CBP BDIs, but not with the other compounds (Figure 2F). Thus, aggregate formation and cytotoxicity are diminished upon treatment with p300/CBP-specific BDIs.

p300/CBP Bromodomains Co-localize with Aggregates and Regulate Aggregate Formation

The effects of BDI treatment suggested that p300/CBP proteins are mechanistically involved in the acetylation-dependent aggregation process. We therefore tested whether expressing wild-type (WT) CBP, or a derivative containing the bromodomain, 3BRD, expressed at levels similar to that of WT CBP, could be incorporated into the aggregates (Figures 3A and 3B). In untreated cells, both WT CBP and 3BRD proteins localized to the nucleus, with a small proportion of the protein retained in the cytoplasm (Figure 3B). However, the amyloid-like aggregates that appeared in the cytoplasm of cells treated with SAHA co-localized with both WT CBP and 3BRD (Figure 3B); the unfused GFP protein was distributed evenly throughout the cell without any propensity to aggregate under SAHA treatment conditions (Figure 3B).

Following on, we assessed whether endogenous p300/CBP was present and found both p300 and CBP to similarly co-localize with the aggregates upon SAHA treatment in different cell lines (Figures 4 and S5). Most significantly, co-treatment with p300/CBP-specific BDIs reduced the amount of amyloid-like aggregates and co-localization with p300/CBP, whereas the other inhibitors had little effect (Figures 4 and S5). The active BDIs caused a partial recovery of p300/CBP to nuclei (Figures 4 and S5), and similar effects were observed with SAHA and CXD101 treatment and in different cell lines (Figures 4 and S5). Moreover, the effect of p300/CBP BDIs was unlikely to be mediated through general effects on acetylation, because analysis of the acetylation level of a number of proteins, such as histone H3, failed to show any significant effect (Figure 4D). Furthermore, we silenced p300 and CBP using small interfering RNA (siRNA), which caused a significant reduction in aggregation upon treatment of the cells with SAHA compared with the control siRNA treatment (Figures 5A and 5B). We quantitated the effect by flow cytometry, which indicated that the APF in p300 or CBP silenced cells was significantly decreased (Figures 5C and 5D). Thus, amyloid-like aggregation in SAHA-treated cells occurs in a fashion dependent upon p300/CBP bromodomains.

Amyloid-like Aggregates Harbor Components of the Proteostatic Machinery

Although the results suggest that p300/CBP bromodomains are involved in aggregate formation, we were interested to characterize the aggregate proteome. Thus, we devised a biochemical filter-based procedure to purify aggregates from SAHA-treated cells, and subjected the purified material to a candidate-based investigation followed by unbiased mass spectrometry (Figure S6A). As expected, the aggregates contained proteins known to undergo lysine acetylation, such as α-tubulin and histone H3 (Figures 6A and S6B). Proteins involved in diverse aspects of proteostasis were present, such as the chaperones heat shock protein 70/heat shock cognate protein 70 (HSP70/HSC70), the autophagy regulator p62 (Bjorkoy et al., 2005), and the proteasomal component 26S S2 subunit, in addition to ubiquitinated proteins (Figures 6A and S6B). Some of these proteins co-localized with the aggregates, for example p62, where its co-localization increased with SAHA concentration (Figure S6C).

The candidate-based approach was supplemented with an unbiased proteomics analysis of the composition of the aggregates by mass spectrometry, whereby a broader group of proteins, many still connected with proteostasis, was identified (Figure 6B). A major functional protein cluster centered around chaperone function, including HSP70 and HSP90 (Figure 6B), and another cluster was connected with protein translation, with ribosome subunits and other components of the translation machinery, such as eukaryotic translation elongation factor 1α (Figure 6B). Analysis of the acetyl-lysine content found that many of the proteins had a high content of lysine residues and had been previously shown to be acetylated (Table S2), including ribosomal proteins, proteins associated with translation such as EEF1A1, EEF1A2, and TUFM (Tu translation elongation factor, mitochondrial), molecular chaperones in the HSP70 and HSP90 family, and histones (Table S2). The aggregate proteome showed on average an increased percentage of lysine residues per protein, namely 7.5% compared with 5.5% or 5.8% in the total cellular proteome (Jones et al., 1992; Tourasse and Li, 2000). It is possible that the high acetyl-lysine content explains their propensity to form aggregates.

Lysine Acetylation Affects Translation and UPS Function

The fact that many proteins involved in protein translation were found in the aggregates led us to hypothesize that protein acetylation and aggregate formation affects protein translation. We therefore tested whether protein translation was affected in SAHA-treated cells and the effect of co-treatment with the p300/CBP BDIs. SAHA-treated cells exhibited a strong decrease in protein translation (Figures 6C and S7A), measured by the incorporation of puromycin into nascent polypeptide chains (Schmidt et al., 2009). Compared with the control treatment, there was an approximate 50% reduction in the occurrence of nascent polypeptide chains when cells were treated with SAHA (Figures 6C and S7A). The p300/CBP BDIs were also

(B) U2OS cells were transfected with an empty vector (pcDNA3), GFP, full-length WT GFP-CBP (1–2,442), or GFP-BRD3 (868–1,341), treated 1 day later with DMSO (–) or SAHA (5 μM), and 24 hr later fixed, permeabilized, and stained with Proteostat. Representative examples of images are shown. Panels on the right represent the enlarged area shown by a dashed square on the panels to the left, and aggregates which co-localize with GFP-tagged full-length WT CBP (1–2,442) or 3BRD-GFP (868–1,341) appear as yellow/orange in the images. DAPI (blue) was used as a nuclear counterstain.

Please cite this article as: Olzscha et al., CBP/p300 Bromodomains Regulate Amyloid-like Protein Aggregation upon Aberrant Lysine Acetylation, Cell Chemical Biology (2017), http://dx.doi.org/10.1016/j.chembiol.2016.11.009

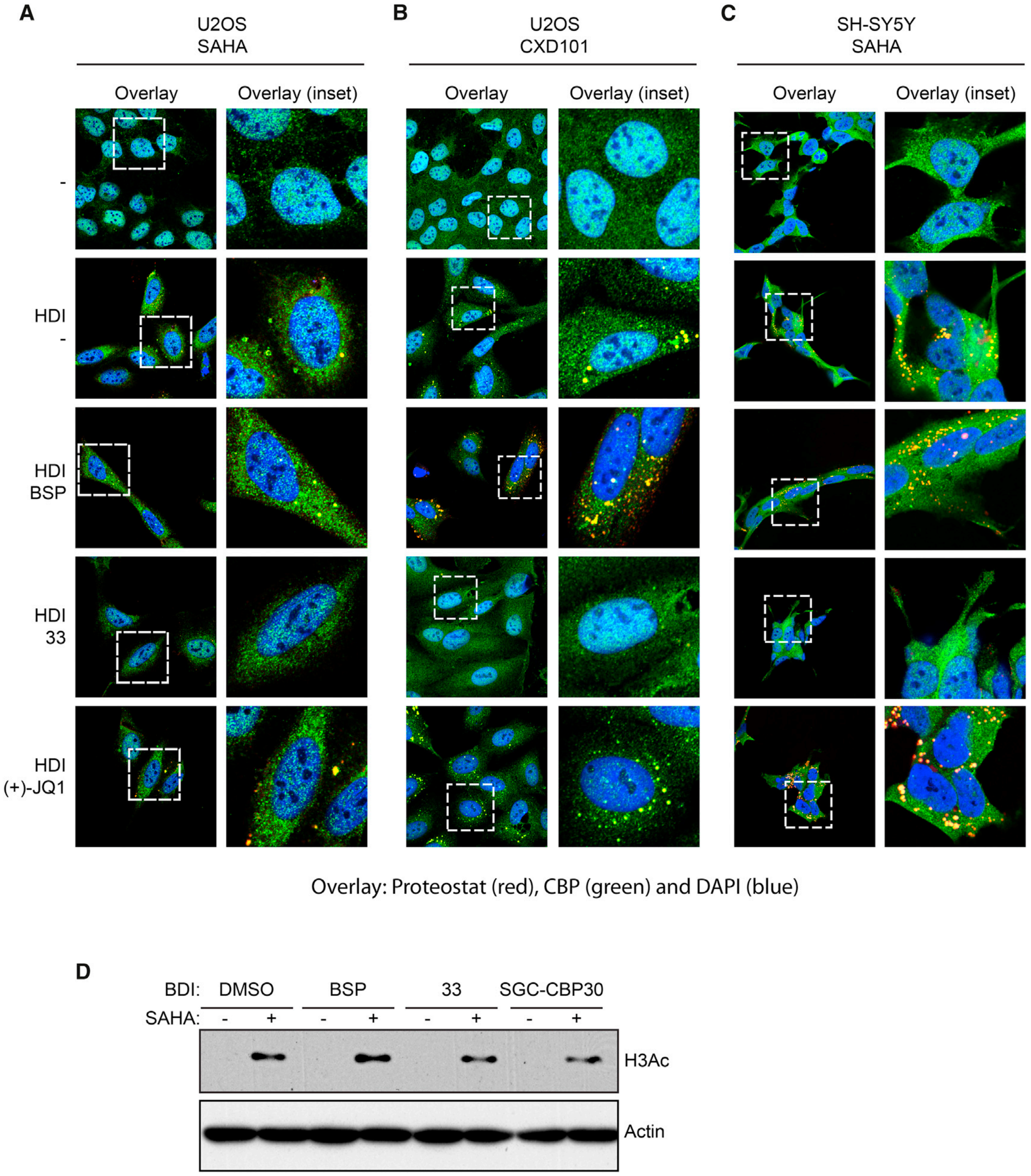

Figure 4. p300/CBP-Specific BDIs Reduce the Formation of Aggregates upon Hyperacetylation

(A) U2OS cells were treated with SAHA (5 μM) or DMSO (−) with or without the indicated BDIs (2.5 μM). After 24 hr the cells were fixed, permeabilized, and stained with Proteostat (red), anti-CBP (green), and DAPI (blue) as a nuclear counterstain, and analyzed by confocal microscopy. Panels on the right of each pair represent the enlarged area shown by a dashed square on the left panels. Aggregates stained with Proteostat that co-localize with CBP appear as yellow/orange in the images. BSP, bromosporine; 33, compound 33.

(B) U2OS cells were treated with CXD101 (5 μM) or DMSO (−) with or without the respective BDIs (2.5 μM). After 24 hr the cells were fixed, permeabilized, and stained with Proteostat (red), anti-CBP (green), and DAPI (blue) as a nuclear counterstain, and analyzed by confocal microscopy. Panels on the right of each pair represent the enlarged area shown by a dashed square on the left panels. Aggregates stained with Proteostat that co-localize with CBP appear as yellow/orange in the images. BSP, bromosporine; 33, compound 33.

(C) SH-SY5Y cells were treated with SAHA (5 μM) or DMSO (−) with or without the respective BDIs (2.5 μM). After 24 hr the cells were fixed, permeabilized, and stained with Proteostat (red), anti-CBP (green), and DAPI (blue) as a nuclear counterstain, and analyzed by confocal microscopy. Panels on the right of each pair represent the enlarged area shown by a dashed square on the left panels. Aggregates stained with Proteostat that co-localize with CBP appear as yellow/orange in the images (see also Figure S5). BSP, bromosporine; 33, compound 33.

(D) Corresponding immunoblot measuring acetylated histone H3Ac, which demonstrates that the pan-HDAC inhibitor was active, the appearance of hyper-acetylation of proteins, and the influence of two CBP/p300-specific BDIs. U2OS cells were treated with SAHA (5 μM) and the respective BDIs (2.5 μM) and then harvested after 24 hr. β-Actin was used as a loading control.

See also Figures S3 and S5.

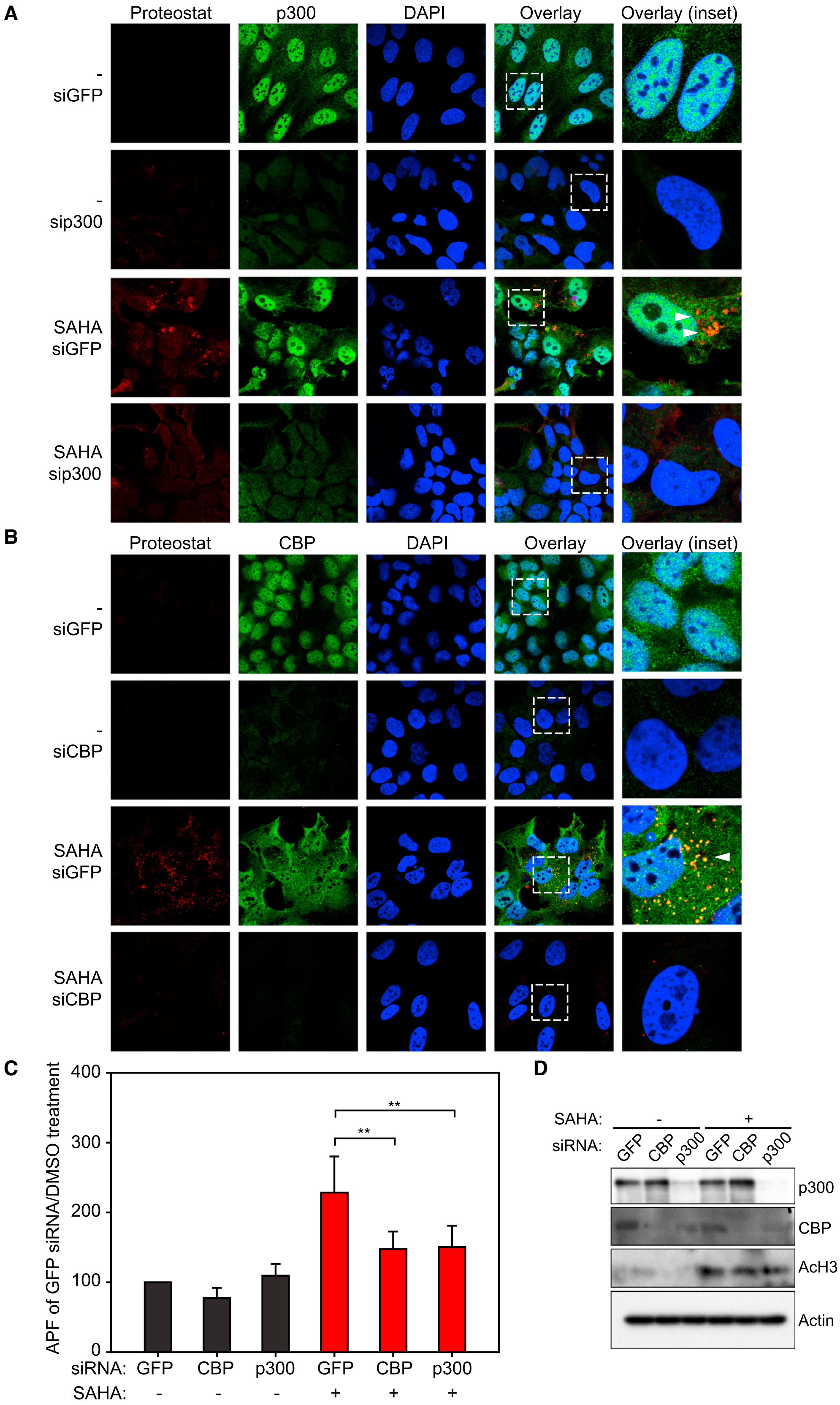

(legend on next page)

able to restore protein translation up to 80%, contrasting with the minimal effect of the other BDIs (Figures 6C and S7A). It appears, therefore, that formation of protein aggregates upon pan-HDAC inhibitor treatment and the role of p300/CBP are linked to protein translation.

We also assessed whether the UPS as part of the cellular proteostasis was altered in SAHA-treated cells and thereafter, the role of bromodomain proteins. We measured the activity of the UPS by means of the expression level of a reporter protein Ub-EGFP, which accumulates upon UPS impairment (Figures 6D and S7B). Significantly, SAHA treatment caused an increase in the Ub-EGFP signal, indicating that UPS activity was defective (Figures 6D and S7B). We then investigated whether there was a statistical relationship between UPS activity and formation of amyloid-like aggregates upon HDAC inhibitor treatment, with and without co-treatment with BDIs. Staining with Proteostat increased after treatment of the stable Ub-EGFP cells with SAHA (Figures 6D and S7B). The Ub-EGFP cells were also co-treated with BDIs, then stained with Proteostat and analyzed by flow cytometry, together with the level of Ub-EGFP. The data are presented graphically as UPS impairment (upper left and upper right quadrant) and formation of aggregates (lower right and upper right quadrant) in the presence and absence of co-treatment with BDI (Figure S7B). There was a linear relationship between the level of amyloid-like aggregates and UPS impairment (Figure 6E), suggesting a functional relationship between the two parameters. Furthermore, the p300/CBP BDIs were most effective at relieving UPS impairment, whereas the other BDIs had insignificant effects (Figure 6E). The coincidental relationship between the effect of p300/CBP BDIs on aggregate formation and UPS impairment argues strongly that p300/CBP bromodomains are linked to the UPS through protein aggregation.

Huntingtin Aggregates and Bromodomain Inhibitors

The pathologically elongated form of huntingtin (Htt) contains a trinucleotide expansion resulting in a polyglutamine extension that has a propensity to form amyloid aggregates (DiFiglia et al., 1997). CBP has been suggested to be involved in forming Htt aggregates (Nucifora et al., 2001; Steffan et al., 2000). Given the results presented here highlighting the role of p300/CBP proteins in the formation of amyloid-like protein aggregates, we tested whether the p300/CBP BDIs could affect Htt aggregates. In U2OS cells expressing either Htt with a 20Q (hemagglutinin [HA]-Htt-20Q) or 96Q stretch (HA-Htt-96Q), Htt-20Q was evenly distributed in cells with no signs of aggregation, whereas cells expressing Htt-96Q exhibited aggregates (Figures 7A and 7B). Htt-96Q occurred as large aggregates in both the nucleus and cytoplasm and stained with Proteostat, whereas HA-Htt-20Q did not (Figure 7A). HA-Htt-96Q could be also stained with X-34, indicating an amyloid-like structure (Figure 7B). Cells expressing either Htt-20Q or Htt-96Q were treated with each BDI, and the number of aggregates measured in the IN Cell Analyzer. Significantly, p300/CBP BDIs caused a modest but reproducible level of inhibition of Htt-96Q aggregation (Figure 7C); immunoblotting of Htt-96Q showed little difference in the protein level (Figure S7C), suggesting that the effect was due to the direct effect of p300/CBP BDIs on the aggregation process. Since CBP interacts with Htt (Nucifora et al., 2001; Steffan et al., 2000), we also addressed whether treatment with the p300/CBP BDIs could influence the interaction. Both endogenous CBP as well as ectopic WT CBP co-localized with the Htt-96Q aggregates (Figures 7D and S7D). Significantly, treatment with the p300/CBP BDIs reduced the level of co-localization (Figures 7D and S7D). Thus, the results suggest that p300/CBP bromodomains are involved in formation of the Htt-96Q aggregates.

DISCUSSION

Amyloid fibrils are insoluble fibrous protein aggregates that contribute to a variety of diseases, including Alzheimer's disease, Parkinson's disease, and Huntington's disease (Chiti and Dobson, 2006; Dobson, 2003). It has been suggested that amyloid-like aggregates provide a protective role that the cell imposes to overcome the toxic effects of protein misfolding (Arrasate et al., 2004), but in doing so the aggregates disturb proteostasis and other cellular functions, leading to cell death (Olzscha et al., 2011). Because amyloid-like aggregates occur in a wide variety of tissues and diseases, and in addition share common structural features, there has been a great deal of interest in developing therapeutic agents that inhibit their formation and limit their toxicity (Powers et al., 2009).

An important conclusion from this study is that hyperacetylation of proteins has a global effect on amyloid-like protein aggregation and causes a general proteostatic failure (Figure 7E). Protein hyperacetylation occurs naturally in cells, for example, by cell stressors such as reactive oxygen species (Mackeh et al., 2014) and upon starvation (Geeraert et al., 2010). Our

Figure 5. Depletion of CBP/p300 Reduces the Formation of Aggregates upon HDI Treatment

(A) U2OS cells were transiently transfected with either a control siGFP or sip300 for 4 days. Twenty-four hours before fixation, cells were treated with SAHA. Cells were stained with Proteostat (red channel) and anti-p300 (green channel), and DAPI was used as a nuclear counterstain (blue). Arrowheads indicate Proteostat-positive aggregates. Co-localization of Proteostat-positive aggregates with p300 appears orange/yellow in the overlay. Panels on the right of each pair represent the enlarged area shown by a dashed square on the panels to the left.

(B) U2OS cells were transiently transfected with either a control siGFP or siCBP for 4 days. Twenty-four hours before fixation, cells were treated with SAHA. Cells were stained with Proteostat (red channel) and anti-CBP (green channel), and DAPI was used as a nuclear counterstain (blue). Arrowheads indicate Proteostat-positive aggregates. Co-localization of Proteostat-positive aggregates with CBP appears orange/yellow in the overlay. Panels on the right of each pair represent the enlarged area shown by a dashed square on the panels to the left.

(C) U2OS cells were treated with siRNAs for 4 days (control siGFP, sip300, or siCBP) and subsequently with SAHA (5 μM), and 24 hr later fixed, permeabilized, and stained with Proteostat. At least 20,000 cells were measured by FACS and the mean fluorescence recorded, which was normalized to the siGFP/DMSO-only (−) treated control. The data were derived from six independent biological replicates and the aggregation propensity factor (APF) was calculated. SAHA-treated cells are depicted in red and DMSO-treated cells in black. Level of statistical significance is indicated (**$p \leq 0.01$). Error bars denote SD.

(D) Immunoblot showing the protein level of CBP and p300 in siRNA-treated cells. Cells were treated for 4 days and 24 h before lysis, DMSO (−) or SAHA was added. β-Actin was used as a loading control, and an antibody against acetylation of the N terminus of histone 3 served as a control for the effect of SAHA.

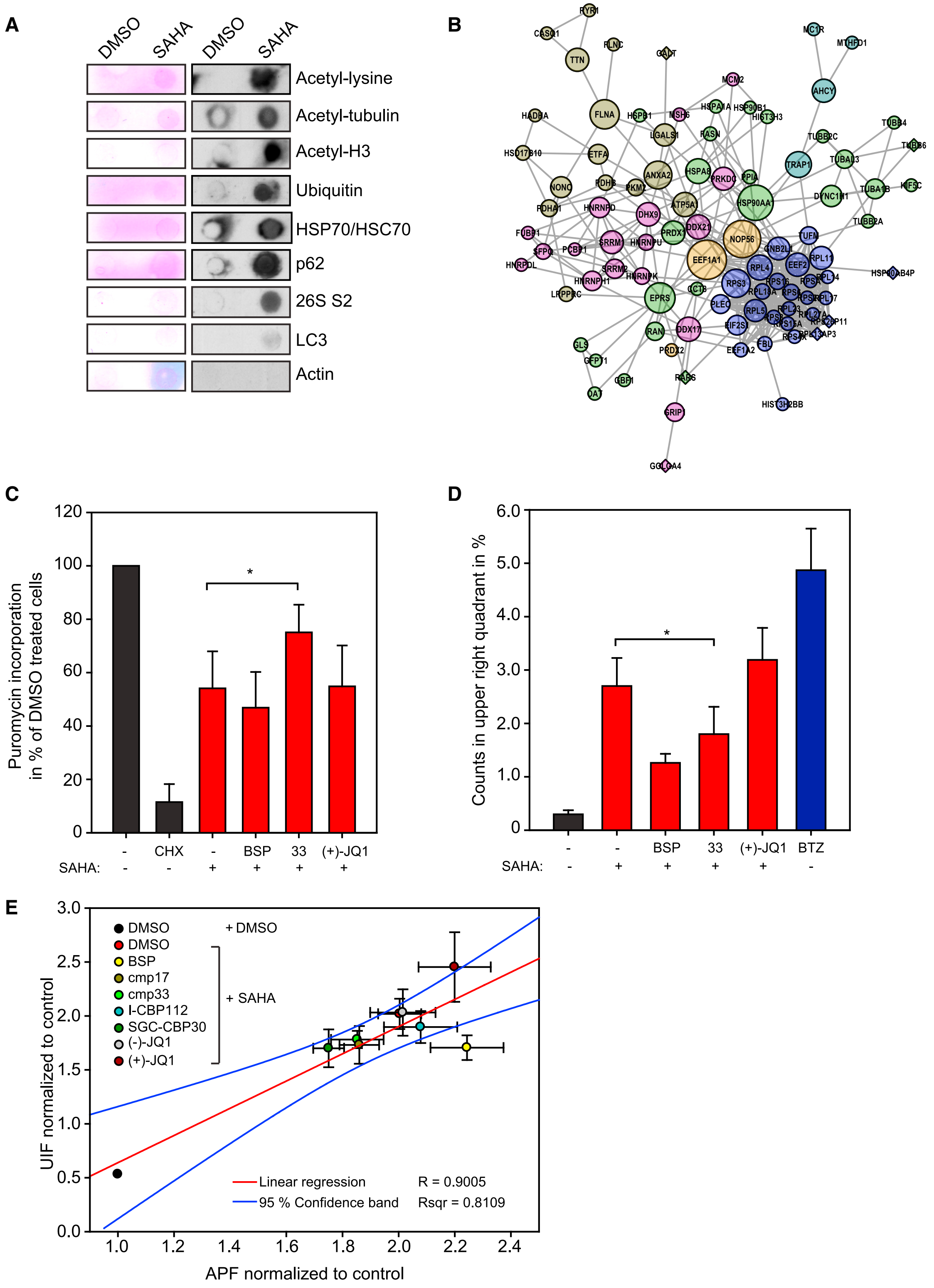

Figure 6. Composition of the HDAC Inhibitor-Induced Aggregates and Effect of CBP/p300-Specific BDIs

(A) U2OS cells were treated with either DMSO or SAHA (5 μM) for 24 hr and then harvested; membranes and DNA were removed and the remaining aggregate fraction treated with detergent. The material captured in the filter retardation assay was visualized by Ponceau S staining or immunostaining with the indicated antibodies (see also Figures S6A and S6B).

(legend continued on next page)

ability to define the aggregates as amyloid-like reflected their staining with different dyes specific for amyloid-like structures and protein aggregates (Games et al., 1995; Shen et al., 2011; Styren et al., 2000). The aggregates contained a concentration of acetylated proteins, and through a chemical biology-led approach using small-molecule BDIs with pre-defined specificities (Filippakopoulos et al., 2010; Hay et al., 2014; Picaud et al., 2015), we established the p300/CBP bromodomain protein family to be mechanistically and functionally involved in the aggregation process. Most notably, small-molecule inhibitors with specificity for the p300/CBP bromodomain family reduced the appearance of the aggregates, suggesting that the integrity and function of p300/CBP bromodomains is rate limiting in forming the aggregates. Furthermore, the presence of the aggregates coincided with impairment of the UPS and protein translation. The results therefore suggest that the proper balance between acetylation and deacetylation of lysine residues is required to maintain protein homeostasis in the cell.

Our understanding of the role of protein aggregation and amyloid formation has highlighted more than 50 human diseases where protein misfolding and conversion into amyloid-like aggregates plays a role in the pathology (Chiti and Dobson, 2006). Surprisingly, there appears to be little similarity in the overall sequence of the proteins identified in amyloid-like aggregates, and it has been suggested that the amyloid structure might be adoptable by virtually any polypeptide able to acquire a generic structure rich in β sheet (Dobson, 2001). The characterization of the amyloid-like aggregates described here indicates that they contain a high level of acetylated lysine proteins, as well as a variety of components connected with diverse aspects of protein quality control, and further that CBP/p300 bromodomains are directly involved in the aggregation process. The high content of lysine residues of many proteins in the aggregates may explain why they are susceptible to protein hyperacetylation upon treatment with HDAC inhibitors and more prone to subsequent aberrant interactions with other proteins.

An analysis of Htt allowed us to assess the impact of lysine acetylation on the aggregates formed by a pathologically relevant protein (DiFiglia et al., 1997). Expansion of a CAG trinucleotide repeat in pathologically mutant forms of Htt leads to the presence of extended polyglutamine tracts, which have been suggested to sequester CBP and related proteins, possibly through the polyglutamine region in CBP, and alter their normal biological roles (McCampbell et al., 2000; Nucifora et al., 2001; Steffan et al., 2000). Because of the relationship that we established between p300/CBP proteins and the amyloid-like aggregates, we reasoned that p300/CBP BDIs might affect the aggregates formed by an expanded Htt. Significantly, the CBP/p300-specific BDIs had a modest but reproducible effect on the Htt aggregates, suggesting that p300/CBP bromodomains contribute to aggregate formation of the elongated form of Htt and, therefore, the pathology of the disease. In turn, these results suggest that BDI-based drugs might provide a therapeutic strategy for treating Huntington's disease and related pathologies (Kirilyuk et al., 2012; Min et al., 2015; Nucifora et al., 2001; Steffan et al., 2000).

Our results established a correlation between amyloid-like aggregates caused by aberrant lysine acetylation and deficits in the UPS and translational activity, suggesting that the protein aggregation causes a global perturbation in proteostasis. The fact that p300/CBP proteins are rate limiting for amyloid-like aggregate formation provides further support for a causative relationship between lysine acetylation and readers of the acetylation mark. Furthermore, our results also bear on the mechanisms through which the cytotoxic effects of HDAC inhibitors are mediated by implying that global effects on protein aggregation make an important contribution. Moreover, protein aggregation may occur in normal cells and thereby contribute to the side effects of HDIs noted in clinical studies (New et al., 2012).

In conclusion, our study describes lysine acetylation, in addition to its role as a mediator of specific regulatory protein interactions through its "reader-writer" interplay, as a process that influences protein aggregation, thereby affecting proteostasis and cell viability. Our results thus highlight a new function for

(B) U2OS cells were treated with either DMSO or SAHA (5 μM) for 24 hr and then harvested; membranes and DNA were removed and the remaining aggregate fraction treated with detergent. The aggregated proteins were trypsinized and subjected to tandem mass spectrometry analysis. Proteins were grouped according to their biological role, which is symbolized by the colors. The size of the symbols represent the betweenness-centrality of the proteins, and the circles represent proteins known to be acetylated, either determined to be so in this study or by mining Phosida, UniProt, MaxQB, CPLM, and PhosphoSitePlus (see also Figure S6A).

(C) Cells were treated with SAHA (5 μM) and the indicated BDIs. The effect on mRNA translation was visualized by incorporation of puromycin in the nascent polypeptide chain after immunoblotting with a monoclonal antibody against puromycin in polypeptide chains. For comparison, Ponceau S served as a loading control. The intensities of the anti-puromycin signal in the different lanes were quantified and normalized against the intensities of the Ponceau S staining. The average of five independent experiments is shown, normalized to puromycin incorporation in percentage of DMSO (−)-treated cells. Level of statistical significance is indicated (*$p \leq 0.05$). Error bars denote SD. Quantification of the independent biological replicates as shown in Figure S7A. BSP, bromosporine; 33, compound 33; (+)-J, (+)-JQ1.

(D) HEK293T cells stably expressing Ub-EGFP were treated with 5 μM of the HDAC inhibitor and with the indicated BDIs. Cells were then analyzed by flow cytometry; green fluorescence (FL1 channel) of the Ub-EGFP is plotted on the y axis and red fluorescence of Proteostat staining (FL3 channel) on the x axis, as shown in Figure S7B. Quadrants were chosen according to the DMSO (−) control, which is set to the lower left quadrant, and the FL3 mean fluorescence in the upper right quadrant of the gated cells was quantified. Shown are three biological replicates, each in technical triplicates. Level of statistical significance is indicated (*$p \leq 0.05$). Error bars denote SD. BSP, bromosporine; 33, compound 33; (+)-J, (+)-JQ1.

(E) HEK293T cells stably expressing Ub-EGFP were treated with SAHA (5 μM) and the indicated BDIs (2.5 μM). Cells were then fixed, permeabilized, and stained with Proteostat. FACS was carried out and the cells were measured in the FL1 channel to detect accumulated Ub-EGFP and in the FL3 channel to detect Proteostat-positive aggregates. After gating and compensation, the mean fluorescence intensities in the upper right quadrants were plotted against each other. The data were derived from at least three independent biological replicates, each in technical triplicates. Linear regression resulted in $R^2 = 0.8109$. BSP, bromosporine.

See also Figures S6 and S7.

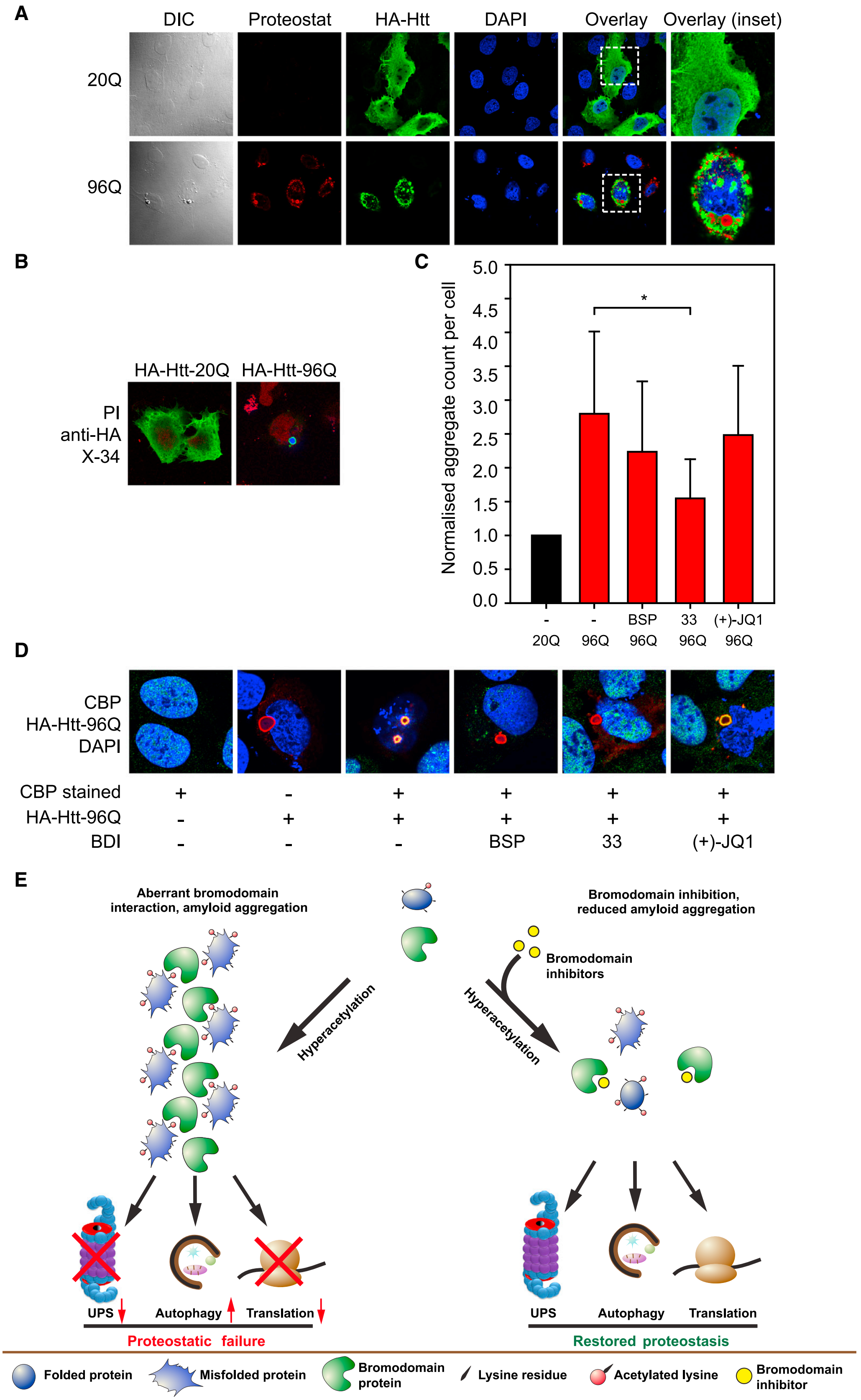

(legend on next page)

lysine acetylation as a global regulator of cellular proteostasis, and further suggest a role in diseases where aberrant protein folding contributes to the pathology.

SIGNIFICANCE

Acetylation is one of the most common post-translational modifications of proteins. We demonstrate that hyperacetylation of proteins leads to amyloid-like aggregation involving proteins that function in proteostasis. p300/CBP bromodomain proteins are involved in the aggregation process, and p300/CBP BDIs partially abrogate protein aggregation. Significantly, protein aggregation coincides with defective proteostasis, which is relieved by p300/CBP BDIs. Importantly, the aggregation of Htt with an elongated polyglutamine stretch is influenced by p300/CBP BDIs. These results highlight a global role for acetylation and bromodomain readers in protein aggregation and proteostasis, and suggest that p300/CBP BDIs have potential in the treatment of protein-misfolding diseases.

EXPERIMENTAL PROCEDURES

Further details of the procedures used in this study are described in Supplemental Experimental Procedures.

Plasmids

A cell line stably expressing Ub(G76V)-EGFP in HEK293T cells was used for the experiments and was kindly provided by F.-Ulrich Hartl. pEGFP-N1 was used as a control plasmid for EGFP expression only. HA-tagged Htt constructs with 20Q and 96Q stretches were kindly provided by F.-Ulrich Hartl. GFP-tagged CBP constructs or GFP-tagged bromodomain constructs were created via the MultiSite Gateway System (Life Technologies, 12537100). The construct containing the 3× bromodomain from CBP contains an NLS sequence at the N terminus (Philpott et al., 2014).

Bromodomain Inhibitors

The different BDIs are listed in Table S1 and have been described previously (Filippakopoulos et al., 2010; Hay et al., 2014; Picaud et al., 2015).

Cell Culture and Transient Transfection

Human cells were cultured at 37°C in a humidified 5% CO_2 incubator in DMEM (Sigma) (HEK293T and U2OS) containing 10% fetal bovine serum (FBS) (Labtech), 100 IU/mL penicillin G, and 100 mg/mL streptomycin sulfate (Gibco, Life Sciences, 15140122). HEK293T cells stably expressing Ub-EGFP were maintained in DMEM (Sigma, D6249) containing 10% FBS (Labtech), 100 IU/mL penicillin G, 100 mg/mL streptomycin sulfate (Gibco, Life Sciences), and G418 (Santa Cruz, sc-29065). RIVA and HBL-1 cells were maintained in RPMI (Sigma, R8758) supplemented with the same. SH-SY5Y cells were maintained in Ham's F12/Eagle's minimal essential medium (EBSS) (1:1) (Sigma, M4655 and N6658), 2 mM glutamine (Gibco, Life Sciences), 1% non-essential amino acids (Gibco, Life Sciences), 15% FBS (Labtech), 100 IU/mL penicillin G, and 100 mg/mL streptomycin sulfate (Gibco, Life Sciences). Transient transfection was performed with GeneJuice (Novagen, 70967-4) according to the manufacturer's protocol.

Immunofluorescence Staining

Cells were seeded onto coverslips and after different treatments washed once in PBS and then fixed in 4% paraformaldehyde for 15–45 min. Cells were then permeabilized in PBS containing 0.5% Triton X-100 for 5–30 min, depending on the following applications. Cells were washed twice with PBS and incubated for 20 min in 1% BSA in PBS as blocking reagent. Cells were then incubated overnight with the primary antibodies at 4°C in 1:50 to 1:100 dilutions dependent on the antibody. They were washed three times with PBS for 5 min and incubated for 1–2 hr with the corresponding secondary fluorescence antibodies Alexa Fluor 488 (Invitrogen) or Alexa Fluor 594 (Invitrogen) in a 1:400 dilution at room temperature. Cells were then incubated for 10 min in a 1 μg/mL DAPI solution (ThermoScientific). Cells were washed three times for 10 min in PBS, then washed for 5 s three times in distilled water and covered with fluorescence mounting medium (Dako). For visualization of aggregates in the human cell lines, cells were stained with X-34, a fluorescent derivative of Congo red, which has the advantage that it can pass cell membranes and should specifically detect amyloid-like aggregates (Styren et al., 2000). X-34 was synthesized at the core facility of the Max Planck Institute of Biochemistry and its Department of Cellular Biochemistry (F.-Ulrich Hartl and Karsten Klage). X-34 was dissolved in 1 M Tris solution (pH 8.8), and a drop of 5 M NaOH was added to stabilize the product in the dissolved state and prevent crystallization. From this 1-mM stock solution, a 50-μM working solution in DMEM without fetal calf serum was produced and living cells were incubated for 45 min with the X-34 solution. Cells were then washed twice for 10 min with pre-warmed PBS, then fixed and permeabilized as described above. Propidium iodide at a concentration of 1 μg/mL was used to counterstain X-34-treated cells.

Cells were fixed and permeabilized as described above, and Proteostat (Enzo Life Sciences) was added in a 1:2,000 dilution for 30 min. Cells were then counterstained with DAPI and washed three times with PBS for 10 min after they were mounted as described by Shen et al. (2011). Thioflavin S staining of cells was performed according to the improved protocol (Sun et al., 2002), except that cells were fixed and permeabilized as described above with $KMnO_4$ and NaOH steps being omitted.

Figure 7. Effect of BDIs on Pathologically Elongated Huntingtin

(A) U2OS cells expressing either the exon 1 of huntingtin (Htt) with 20Q or 96Q were fixed, permeabilized, and stained with an HA antibody (shown in green). Proteostat was applied to visualize aggregated structures (red), and nuclei were counterstained with DAPI (blue). Panels on the right represent the enlarged area shown by a dashed square on panels to the left.

(B) U2OS cells expressing HA-Htt-96Q exon 1 form aggregates and are positively stained with X-34 (shown in blue). Nuclei were stained with propidium iodide (PI) (red) and the Htt constructs are shown in green.

(C) U2OS cells expressing HA-Htt-96Q or HA-Htt-20Q were treated with the respective BDI (2.5 μM), then fixed, permeabilized, and stained with Proteostat and DAPI as a nuclear counterstain. The number of Proteostat-positive aggregates was then quantified with an IN Cell Analyzer 1000. The data were derived from at least three independent experiments each in triplicates, and for each condition at least 1,500 cells were analyzed relative to 20Q, which was given an arbitrary value of 1. Level of statistical significance is indicated (*$p \leq 0.05$). Error bars denote SD. The sample expressing HA-Htt-20Q is depicted in black, and HA-Htt-96Q-expressing cells are depicted in red. BSP, bromosporine; 33, compound 33; (+)-J, (+)-JQ1.

(D) U2OS cells expressing HA-Htt-96Q were treated with either DMSO (–) or BDIs (2.5 μM), then fixed, permeabilized, and stained with an antibody against CBP (green), HA (red), and DAPI (blue) as a nuclear counterstain. Co-localization of HA-Htt-96Q aggregates with endogenous CBP is depicted by yellow/orange color. Shown are the overlays from the three different channels. BSP, bromosporine; 33, compound 33; (+)-J, (+)-JQ1.

(E) Schematic overview of protein aggregation and proteostatic failure upon hyperacetylation and rescue by bromodomain inhibitors. Proteins aggregate upon hyperacetylation and aberrant interactions occur with bromodomain-containing proteins such as CBP/p300. Essential protein quality control mechanisms are affected, such as degradation of proteins via the ubiquitin proteasome system and protein translation. BDIs, and especially those preferentially binding to CBP/p300, diminish the formation of aggregates and also restore to some extent protein degradation and translation.

See also Figure S7.

SUPPLEMENTAL INFORMATION

Supplemental Information includes Supplemental Experimental Procedures, seven figures, and two tables and can be found with this article online at http://dx.doi.org/10.1016/j.chembiol.2016.11.009.

AUTHOR CONTRIBUTIONS

H.O. designed and performed the experiments and wrote the manuscript. O.F. and S.K. provided the inhibitors and B.M.K. provided the mass spectrometry expertise. N.B.I.T. conceived the project, directed the research, and wrote the manuscript.

ACKNOWLEDGMENTS

We thank the MRC, CRUK (Program Grant C300/A13058), and the Eurocan Platform within the seventh Framework Program. H.O. was supported by an EMBO long-term fellowship and CRUK. We thank the SGC for providing bromodomain inhibitors and vectors. We thank F.-Ulrich Hartl for the Htt plasmids and the Ub-EGFP stable cell line, and F.-Ulrich Hartl and Karsten Klage for synthesis of the dye X-34. We acknowledge the help of Joanna McGouran for mass spectrometry and Remko Prevo for measurements with the IN Cell Analyzer.

Received: May 4, 2016
Revised: September 23, 2016
Accepted: November 15, 2016
Published: December 15, 2016

REFERENCES

Arrasate, M., Mitra, S., Schweitzer, E.S., Segal, M.R., and Finkbeiner, S. (2004). Inclusion body formation reduces levels of mutant huntingtin and the risk of neuronal death. Nature *431*, 805–810.

Balch, W.E., Morimoto, R.I., Dillin, A., and Kelly, J.W. (2008). Adapting proteostasis for disease intervention. Science *319*, 916–919.

Bjorkoy, G., Lamark, T., Brech, A., Outzen, H., Perander, M., Overvatn, A., Stenmark, H., and Johansen, T. (2005). p62/SQSTM1 forms protein aggregates degraded by autophagy and has a protective effect on huntingtin-induced cell death. J. Cell Biol. *171*, 603–614.

Chan, H.M., and La Thangue, N.B. (2001). p300/CBP proteins: HATs for transcriptional bridges and scaffolds. J. Cell Sci. *114*, 2363–2373.

Chiti, F., and Dobson, C.M. (2006). Protein misfolding, functional amyloid, and human disease. Annu. Rev. Biochem. *75*, 333–366.

Choudhary, C., Kumar, C., Gnad, F., Nielsen, M.L., Rehman, M., Walther, T.C., Olsen, J.V., and Mann, M. (2009). Lysine acetylation targets protein complexes and co-regulates major cellular functions. Science *325*, 834–840.

Choudhary, C., Weinert, B.T., Nishida, Y., Verdin, E., and Mann, M. (2014). The growing landscape of lysine acetylation links metabolism and cell signalling. Nat. Rev. Mol. Cell Biol. *15*, 536–550.

DiFiglia, M., Sapp, E., Chase, K.O., Davies, S.W., Bates, G.P., Vonsattel, J.P., and Aronin, N. (1997). Aggregation of huntingtin in neuronal intranuclear inclusions and dystrophic neurites in brain. Science *277*, 1990–1993.

Dobson, C.M. (2001). The structural basis of protein folding and its links with human disease. Philos. Trans. R. Soc. Lond. B Biol. Sci. *356*, 133–145.

Dobson, C.M. (2003). Protein folding and misfolding. Nature *426*, 884–890.

Filippakopoulos, P., and Knapp, S. (2014). Targeting bromodomains: epigenetic readers of lysine acetylation. Nat. Rev. Drug Discov. *13*, 337–356.

Filippakopoulos, P., Qi, J., Picaud, S., Shen, Y., Smith, W.B., Fedorov, O., Morse, E.M., Keates, T., Hickman, T.T., Felletar, I., et al. (2010). Selective inhibition of BET bromodomains. Nature *468*, 1067–1073.

Games, D., Adams, D., Alessandrini, R., Barbour, R., Berthelette, P., Blackwell, C., Carr, T., Clemens, J., Donaldson, T., Gillespie, F., et al. (1995). Alzheimer-type neuropathology in transgenic mice overexpressing V717F beta-amyloid precursor protein. Nature *373*, 523–527.

Geeraert, C., Ratier, A., Pfisterer, S.G., Perdiz, D., Cantaloube, I., Rouault, A., Pattingre, S., Proikas-Cezanne, T., Codogno, P., and Pous, C. (2010). Starvation-induced hyperacetylation of tubulin is required for the stimulation of autophagy by nutrient deprivation. J. Biol. Chem. *285*, 24184–24194.

Grunstein, M. (1997). Histone acetylation in chromatin structure and transcription. Nature *389*, 349–352.

Hammitzsch, A., Tallant, C., Fedorov, O., O'Mahony, A., Brennan, P.E., Hay, D.A., Martinez, F.O., Al-Mossawi, M.H., de Wit, J., Vecellio, M., et al. (2015). CBP30, a selective CBP/p300 bromodomain inhibitor, suppresses human Th17 responses. Proc. Natl. Acad. Sci. USA *112*, 10768–10773.

Hansen, J.C., Lu, X., Ross, E.D., and Woody, R.W. (2006). Intrinsic protein disorder, amino acid composition, and histone terminal domains. J. Biol. Chem. *281*, 1853–1856.

Hartl, F.U., Bracher, A., and Hayer-Hartl, M. (2011). Molecular chaperones in protein folding and proteostasis. Nature *475*, 324–332.

Hay, D.A., Fedorov, O., Martin, S., Singleton, D.C., Tallant, C., Wells, C., Picaud, S., Philpott, M., Monteiro, O.P., Rogers, C.M., et al. (2014). Discovery and optimization of small-molecule ligands for the CBP/p300 bromodomains. J. Am. Chem. Soc. *136*, 9308–9319.

Hyun, D.H., Lee, M., Halliwell, B., and Jenner, P. (2003). Proteasomal inhibition causes the formation of protein aggregates containing a wide range of proteins, including nitrated proteins. J. Neurochem. *86*, 363–373.

Jones, D.T., Taylor, W.R., and Thornton, J.M. (1992). A new approach to protein fold recognition. Nature *358*, 86–89.

Khan, O., and La Thangue, N.B. (2012). HDAC inhibitors in cancer biology: emerging mechanisms and clinical applications. Immunol. Cell Biol. *90*, 85–94.

Kirilyuk, A., Shimoji, M., Catania, J., Sahu, G., Pattabiraman, N., Giordano, A., Albanese, C., Mocchetti, I., Toretsky, J.A., Uversky, V.N., et al. (2012). An intrinsically disordered region of the acetyltransferase p300 with similarity to prion-like domains plays a role in aggregation. PLoS One *7*, e48243.

Kouzarides, T. (1999). Histone acetylases and deacetylases in cell proliferation. Curr. Opin. Genet. Dev. *9*, 40–48.

Link, C.D., Johnson, C.J., Fonte, V., Paupard, M., Hall, D.H., Styren, S., Mathis, C.A., and Klunk, W.E. (2001). Visualization of fibrillar amyloid deposits in living, transgenic *Caenorhabditis elegans* animals using the sensitive amyloid dye, X-34. Neurobiol. Aging *22*, 217–226.

Mackeh, R., Lorin, S., Ratier, A., Mejdoubi-Charef, N., Baillet, A., Bruneel, A., Hamai, A., Codogno, P., Pous, C., and Perdiz, D. (2014). Reactive oxygen species, AMP-activated protein kinase, and the transcription cofactor p300 regulate alpha-tubulin acetyltransferase-1 (alphaTAT-1/MEC-17)-dependent microtubule hyperacetylation during cell stress. J. Biol. Chem. *289*, 11816–11828.

Marks, P., Rifkind, R.A., Richon, V.M., Breslow, R., Miller, T., and Kelly, W.K. (2001). Histone deacetylases and cancer: causes and therapies. Nat. Rev. Cancer *1*, 194–202.

McCampbell, A., Taylor, J.P., Taye, A.A., Robitschek, J., Li, M., Walcott, J., Merry, D., Chai, Y., Paulson, H., Sobue, G., et al. (2000). CREB-binding protein sequestration by expanded polyglutamine. Hum. Mol. Genet. *9*, 2197–2202.

Min, S.W., Chen, X., Tracy, T.E., Li, Y., Zhou, Y., Wang, C., Shirakawa, K., Minami, S.S., Defensor, E., Mok, S.A., et al. (2015). Critical role of acetylation in tau-mediated neurodegeneration and cognitive deficits. Nat. Med. *21*, 1154–1162.

New, M., Olzscha, H., and La Thangue, N.B. (2012). HDAC inhibitor-based therapies: can we interpret the code? Mol. Oncol. *6*, 637–656.

Nucifora, F.C., Jr., Sasaki, M., Peters, M.F., Huang, H., Cooper, J.K., Yamada, M., Takahashi, H., Tsuji, S., Troncoso, J., Dawson, V.L., et al. (2001). Interference by huntingtin and atrophin-1 with cbp-mediated transcription leading to cellular toxicity. Science *291*, 2423–2428.

Olzscha, H., Schermann, S.M., Woerner, A.C., Pinkert, S., Hecht, M.H., Tartaglia, G.G., Vendruscolo, M., Hayer-Hartl, M., Hartl, F.U., and Vabulas, R.M. (2011). Amyloid-like aggregates sequester numerous metastable proteins with essential cellular functions. Cell *144*, 67–78.

Philpott, M., Rogers, C.M., Yapp, C., Wells, C., Lambert, J.P., Strain-Damerell, C., Burgess-Brown, N.A., Gingras, A.C., Knapp, S., and Muller, S. (2014).

Assessing cellular efficacy of bromodomain inhibitors using fluorescence recovery after photobleaching. Epigenetics Chromatin *7*, 14.

Picaud, S., Fedorov, O., Thanasopoulou, A., Leonards, K., Jones, K., Meier, J., Olzscha, H., Monteiro, O., Martin, S., Philpott, M., et al. (2015). Generation of a selective small molecule inhibitor of the cbp/p300 bromodomain for leukemia therapy. Cancer Res. *75*, 5106–5119.

Powers, E.T., Morimoto, R.I., Dillin, A., Kelly, J.W., and Balch, W.E. (2009). Biological and chemical approaches to diseases of proteostasis deficiency. Annu. Rev. Biochem. *78*, 959–991.

Saha, R.N., and Pahan, K. (2006). HATs and HDACs in neurodegeneration: a tale of disconcerted acetylation homeostasis. Cell Death Differ. *13*, 539–550.

Schmidt, E.K., Clavarino, G., Ceppi, M., and Pierre, P. (2009). SUnSET, a nonradioactive method to monitor protein synthesis. Nat. Methods *6*, 275–277.

Shen, D., Coleman, J., Chan, E., Nicholson, T.P., Dai, L., Sheppard, P.W., and Patton, W.F. (2011). Novel cell- and tissue-based assays for detecting misfolded and aggregated protein accumulation within aggresomes and inclusion bodies. Cell Biochem. Biophys. *60*, 173–185.

Steffan, J.S., Kazantsev, A., Spasic-Boskovic, O., Greenwald, M., Zhu, Y.Z., Gohler, H., Wanker, E.E., Bates, G.P., Housman, D.E., and Thompson, L.M. (2000). The Huntington's disease protein interacts with p53 and CREB-binding protein and represses transcription. Proc. Natl. Acad. Sci. USA *97*, 6763–6768.

Styren, S.D., Hamilton, R.L., Styren, G.C., and Klunk, W.E. (2000). X-34, a fluorescent derivative of Congo red: a novel histochemical stain for Alzheimer's disease pathology. J. Histochem. Cytochem. *48*, 1223–1232.

Sun, A., Nguyen, X.V., and Bing, G. (2002). Comparative analysis of an improved thioflavin-s stain, Gallyas silver stain, and immunohistochemistry for neurofibrillary tangle demonstration on the same sections. J. Histochem. Cytochem. *50*, 463–472.

Theodoulou, N.H., Tomkinson, N.C., Prinjha, R.K., and Humphreys, P.G. (2016). Progress in the development of non-BET bromodomain chemical probes. ChemMedChem *11*, 477–487.

Tourasse, N.J., and Li, W.H. (2000). Selective constraints, amino acid composition, and the rate of protein evolution. Mol. Biol. Evol. *17*, 656–664.

Wang, X., Moore, S.C., Laszckzak, M., and Ausio, J. (2000). Acetylation increases the alpha-helical content of the histone tails of the nucleosome. J. Biol. Chem. *275*, 35013–35020.

Zeng, L., and Zhou, M.M. (2002). Bromodomain: an acetyl-lysine binding domain. FEBS Lett. *513*, 124–128.

Zhao, S., Xu, W., Jiang, W., Yu, W., Lin, Y., Zhang, T., Yao, J., Zhou, L., Zeng, Y., Li, H., et al. (2010). Regulation of cellular metabolism by protein lysine acetylation. Science *327*, 1000–1004.

Cell Chemical Biology, Volume *24*

Supplemental Information

CBP/p300 Bromodomains Regulate Amyloid-like Protein Aggregation upon Aberrant Lysine Acetylation

Heidi Olzscha, Oleg Fedorov, Benedikt M. Kessler, Stefan Knapp, and Nicholas B. La Thangue

Supplemental Information
Supplemental Figures

Fig. S1

A

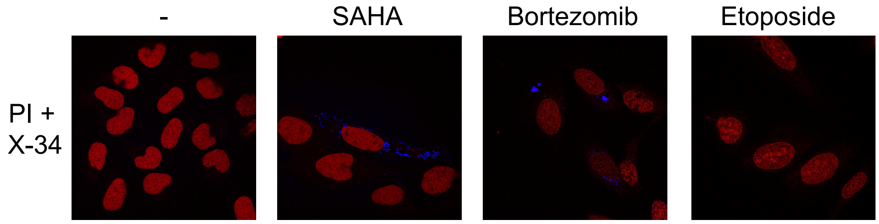

B

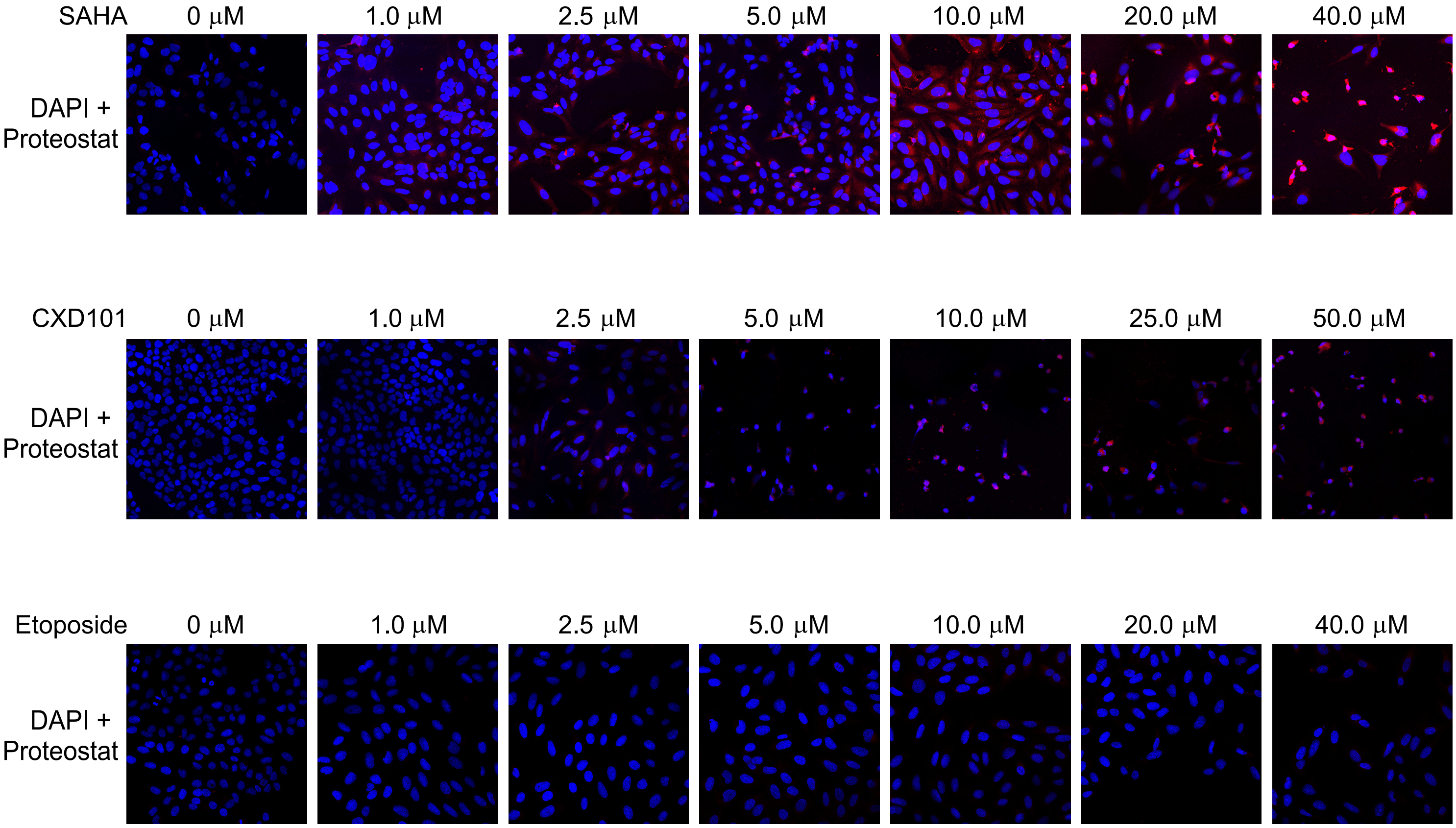

C

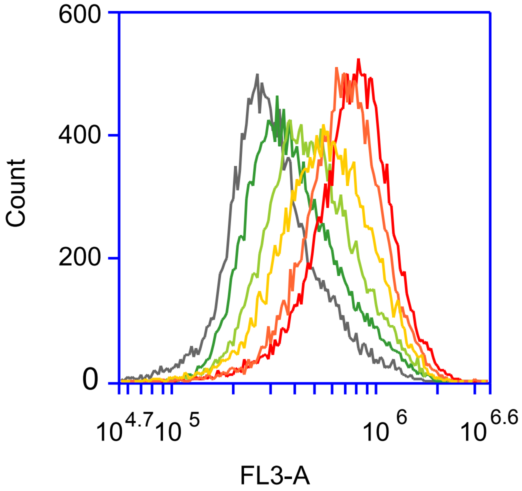

D

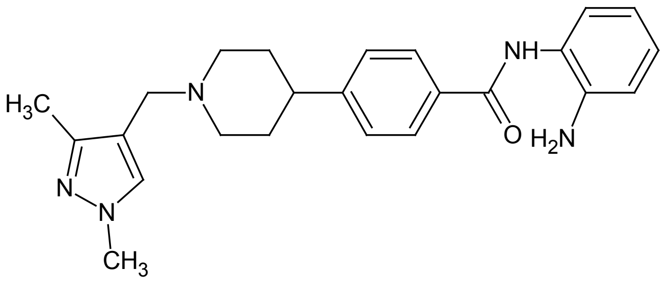

Figure S1 (related to Figure 1)

Detection of aggregates upon dose-escalation treatment with different HDIs.

(A) U2OS cells were treated with SAHA, bortezomib or etoposide for 24 h with concentrations to their respective IC50s. Cells were then incubated with the dye X-34 (blue) specifically recognizing amyloid-like structures in living systems. They were then fixed, permeabilized and propidium iodide (red) was used as a nuclear counterstain.

(B) Staining of U2OS cells upon treatment with increasing concentrations of the HDIs SAHA or CXD101 or the topoisomerase inhibitor etoposide with the dye Proteostat. Cells were treated with indicated concentrations of the compound for 24 h, fixed, permeabilized, and stained with the Proteostat dye in a 1:2000 dilution from the supplier's stock solution (red). They were counterstained with DAPI (blue) and analysed by confocal microscopy, in order to visualize the increasing staining upon SAHA, CXD101 or etoposide treatment.

(C) U2OS cells were treated with either DMSO (grey), or increasing concentrations of SAHA ranging from 1.0 µM (dark green), 2.5 µM (light green), 5.0 µM (yellow), 10.0 µM (orange) and 20.0 µM (red), for 24 h, fixed and permeabilized. Then the cells were stained with Proteostat and analyzed in the FL-3 channel by flow cytometry.

(D) Structure of CXD101.

Fig. S2

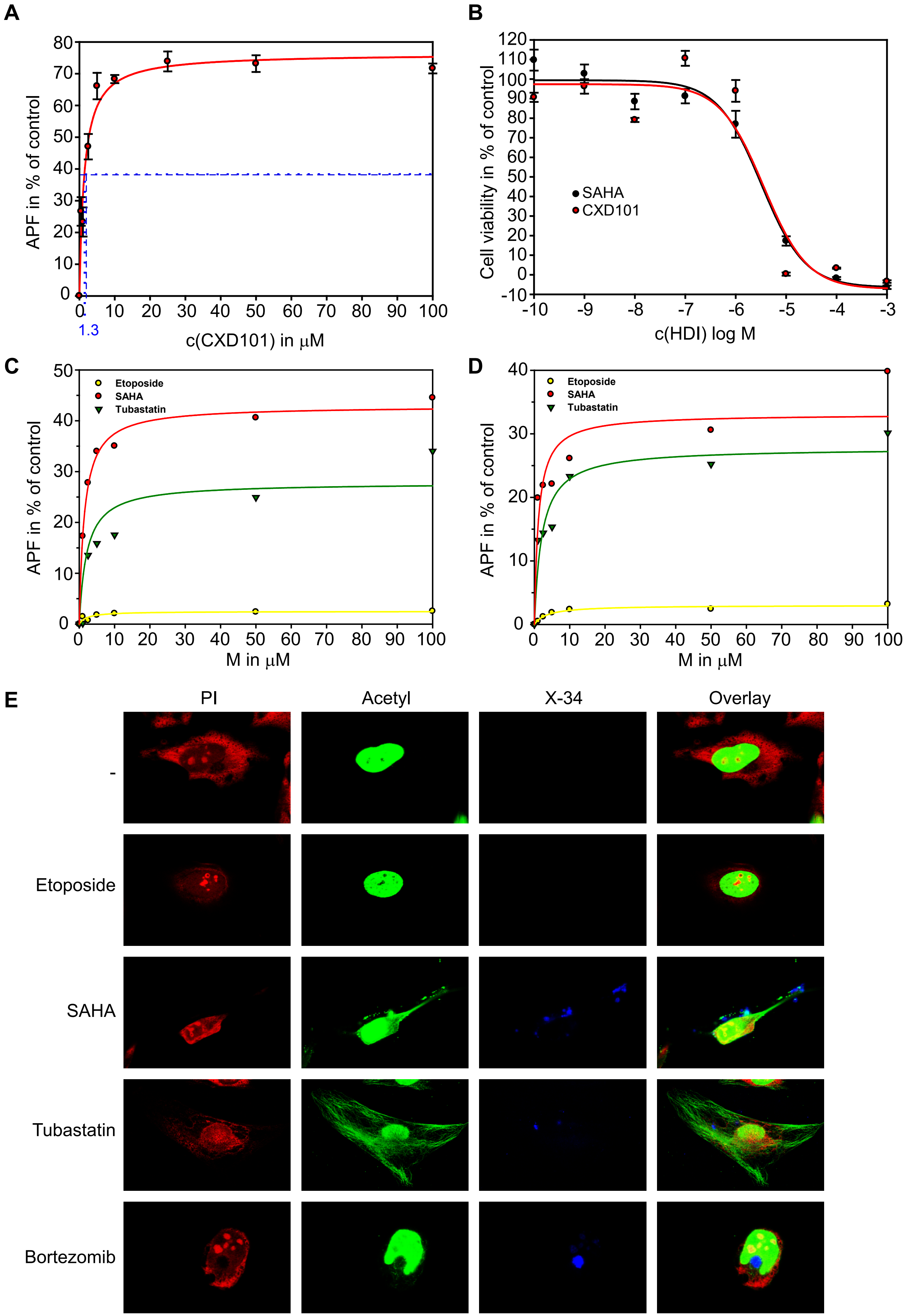

A
APF in % of control
80
70
60
50
40
30
20
10
0
0
10
20
30
40
50
60
70
80
90
100
1.3
c(CXD101) in μM
B
Cell viability in % of control
120
110
100
90
80
70
60
50
40
30
20
10
0
-10
-9
-8
-7
-6
-5
-4
-3
SAHA
CXD101
c(HDI) log M
C
APF in % of control
Etoposide
SAHA
Tubastatin
M in μM
D
APF in % of control
Etoposide
SAHA
Tubastatin
M in μM
E
PI
Acetyl
X-34
Overlay
-
Etoposide
SAHA
Tubastatin
Bortezomib

Figure S2 (related to Figure 1 and 2)

Co-localisation of acetylated proteins with X-34-positive aggregates upon SAHA treatment and quantitation of aggregate formation in different cell lines.

(A) U2OS cells were treated with CXD101, inducing the formation of Proteostat-positive aggregates, as shown for instance in Figure S1B. Cells were measured by flow cytometry in the FL3 channel and the aggregation propensity factor (APF) was calculated. APFs were averaged from three independent biological replicates and APFmax ½ was calculated, indicating the half maximal concentration, aggregation occurs.

(B) Cell viabilities of U2OS cells after treatment with different concentrations of the pan-HDIs SAHA or CXD101 to determine the IC50s and working range for the inhibitors.

Cell viability was determined after 24 h by means of MTT assay. Cell viability was normalized to cells treated DMSO only. Experiments were performed in three biological replicates, each in triplicates; error bars represent the standard deviation (SD). SAHA treated cells are shown in black, whereas values for CXD101 are shown in red.

(C) RIVA cells were treated with SAHA, tubastatin or etoposide inducing the formation of Proteostat-positive aggregates, as shown for instance in Figure S1B. Cells were measured *via* flow cytometry in the FL3 channel and the aggregation propensity factor (APF) was calculated. Shown is one representative experiment.

(D) HBL-1 cells were treated with SAHA, tubastatin or etoposide inducing the formation of Proteostat-positive aggregates, as shown for instance in Figure S1B. Cells were measured *via* flow cytometry in the FL3 channel and the aggregation propensity factor (APF) was calculated. Shown is one representative experiment.

(E) U2OS cells were treated with the indicated drugs and stained with X-34 (blue). After fixation and permeabilization, cells were stained with an antibody against acetylated lysines (green). PI staining was applied to visualize nuclei or nucleated bodies after treatment with the respective drugs (red). Confocal images showing in case of SAHA treatment co-localization of acetylated proteins with X-34 positive aggregates mainly in the cytoplasm.

Fig. S3

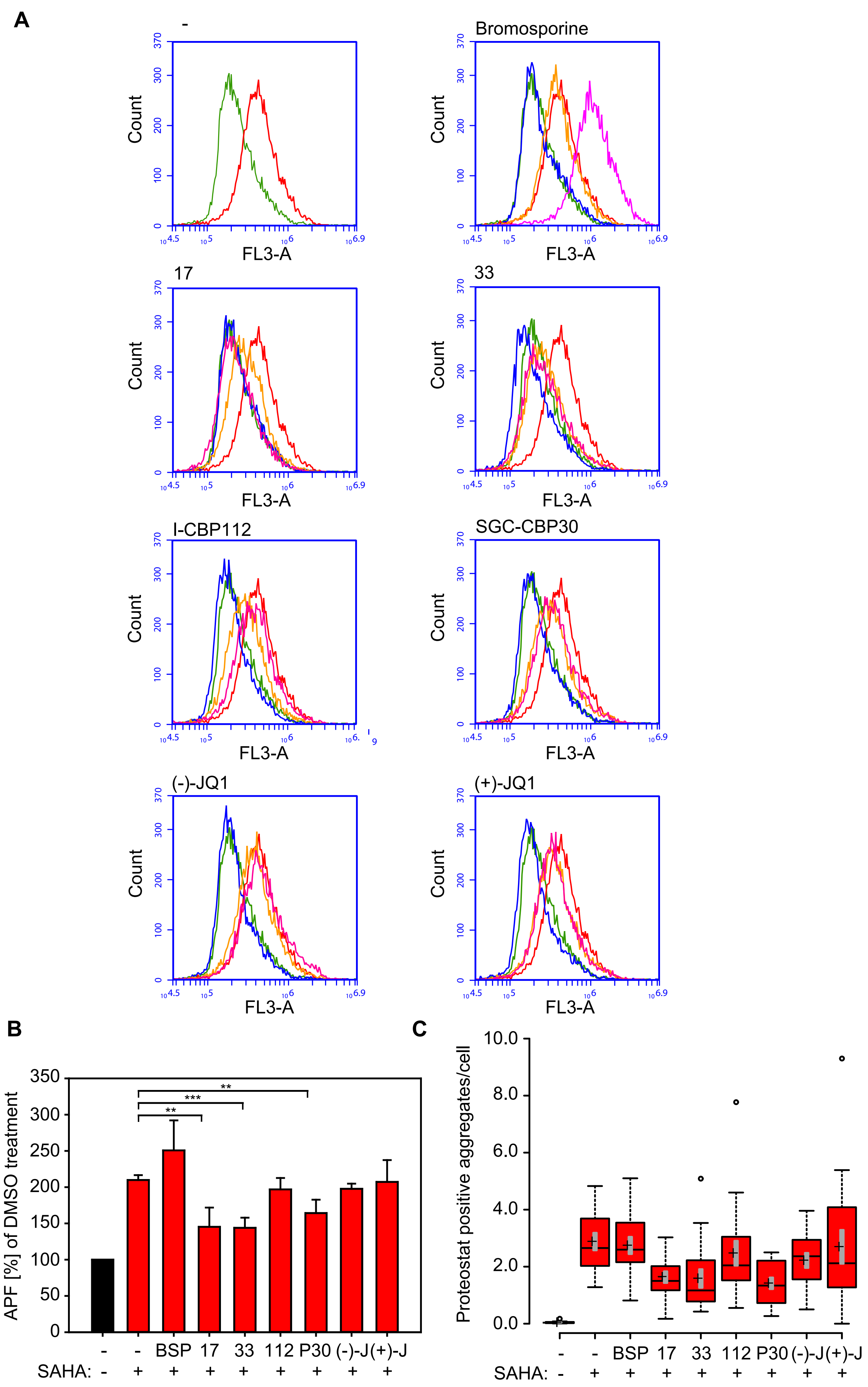

A
-
Bromosporine
17
33
I-CBP112
SGC-CBP30
(-)-JQ1
(+)-JQ1
Count
FL3-A
B
APF [%] of DMSO treatment
- - BSP 17 33 112 P30 (-)-J (+)-J
SAHA: - + + + + + + + +
C
Proteostat positive aggregates/cell
- - BSP 17 33 112 P30 (-)-J (+)-J
SAHA: - + + + + + + + +

Figure S3 (related to Figure 2)

Quantitative assessment of the reduction of aggregates upon BDI treatment

(A) FACS profiles of U2OS cells after co-treatment with SAHA and BDIs stained to detect aggregates. USOS cells were treated with the respective drug combinations, fixed, permeabilized and stained with Proteostat in order to quantitate changes in the aggregation propensity upon treatment. Cells were measured in the FL3 channel and cells displayed in the in FSC/SSC channels (data not shown) were gated to exclude debris, but not to exclude apoptotic cells. In green, cells are represented which were treated with DMSO only (-), red represents SAHA treated cells, blue shows cells which were treated with 2.5 μM of the respective BDI alone, yellow represents cells which are treated with SAHA and 1.0 μM of the respective BDI and in pink cells were treated with SAHA and 2.5 μM of the BDI.

(B) U2OS cells were treated with SAHA (5 μM) and BDIs (2.5 μM) as indicated and 24 h later fixed, permeabilized and stained with Proteostat. At least 20,000 cells were measured by FACS in the FL-3 channel and the mean fluorescence recorded which was normalized to the DMSO (-) only treated control (black), SAHA treated samples are depicted in (red). The data were derived from four independent biological replicates, each performed in three technical replicates and the aggregation propensity factor (APF) was calculated. Level of statistical significance is indicated (** $P \leq 0.001$, *** $P \leq 0.001$) and error bars represent the standard deviation (SD). BSP, bromosporine; 17, compound 17; 33, compound 33; 112, I-CBP112; P30, SGC-CBP30; (-)-J, (-)-JQ1; (+)-J, (+)-JQ1.

(C) U2OS cells were treated with SAHA (5 μM) and BDIs (2.5 μM), then fixed, permeabilized and stained with Proteostat and DAPI as a nuclear counterstain. The number of Proteostat-positive aggregates was then quantified with an IN Cell Analyzer 1000. The data were derived from three independent experiments, for each condition at least 1500 cells were analysed in technical replicates. DMSO-only treatment (-) are depicted in grey, SAHA treated cells in red. Sample means are indicated by crosses, confidence intervals (90%) by grey bars. BSP, bromosporine; 17, compound 17; 33, compound 33; 112, I-CBP112; P30, SGC-CBP30; (-)-J, (-)-JQ1; (+)-J, (+)-JQ1.

Fig. S4

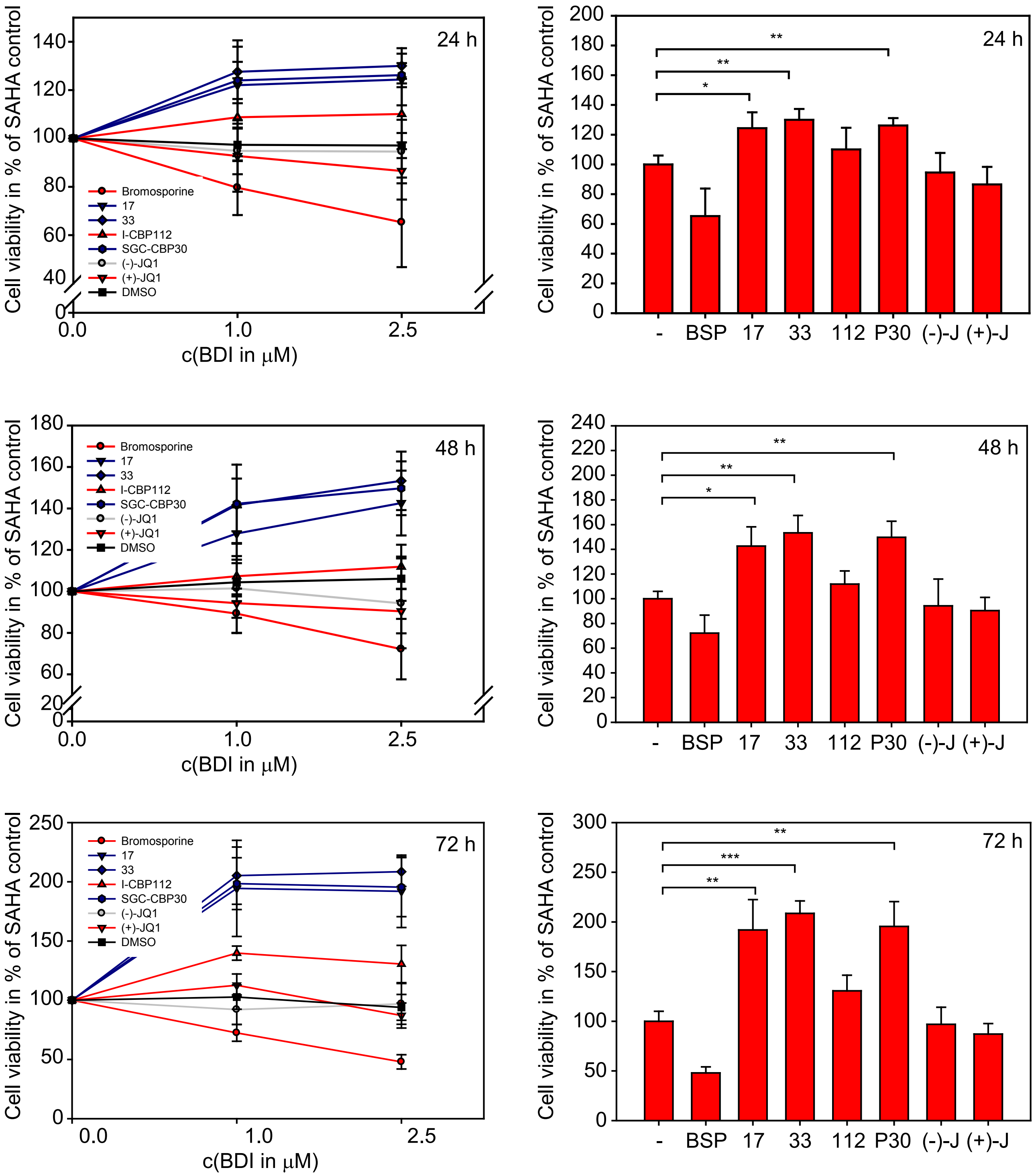

24 h
48 h
72 h
Cell viability in % of SAHA control
c(BDI in μM)
Bromosporine
17
33
I-CBP112
SGC-CBP30
(-)-JQ1
(+)-JQ1
DMSO
- BSP 17 33 112 P30 (-)-J (+)-J

Figure S4 (related to Figure 2)

Cell viabilities of U2OS cells after co-treatment with SAHA in combination with different BDIs and after different time points.

BDIs were applied 2 h before cells were treated with the pan-HDAC inhibitor, and then cell viability was determined after the indicated times by means of MTT assay. Cell viability was normalized to cells treated with SAHA only (indexed in the left panel). Experiments were performed in at least three biological replicates, each in triplicates. Values of the BDIs which are able to rescue the toxic effects of SAHA treated cells are shown in blue in the left panels, whereas values for SAHA or BDIs which do not change the cytotoxicity or increase it, are shown in red, the inactive compound (-)-JQ1 is shown in grey and DMSO treated cells are shown in black. Level of statistical significance is indicated (* $P \leq 0.05$, ** $P \leq 0.01$, *** $P \leq 0.001$), error bars represent the standard deviation (SD). BSP, bromosporine; 17, compound 17; 33, compound 33; 112, I-CBP112; P30, SGC-CBP30; (-)-J, (-)-JQ1; (+)-J, (+)-JQ1.

Fig. S5

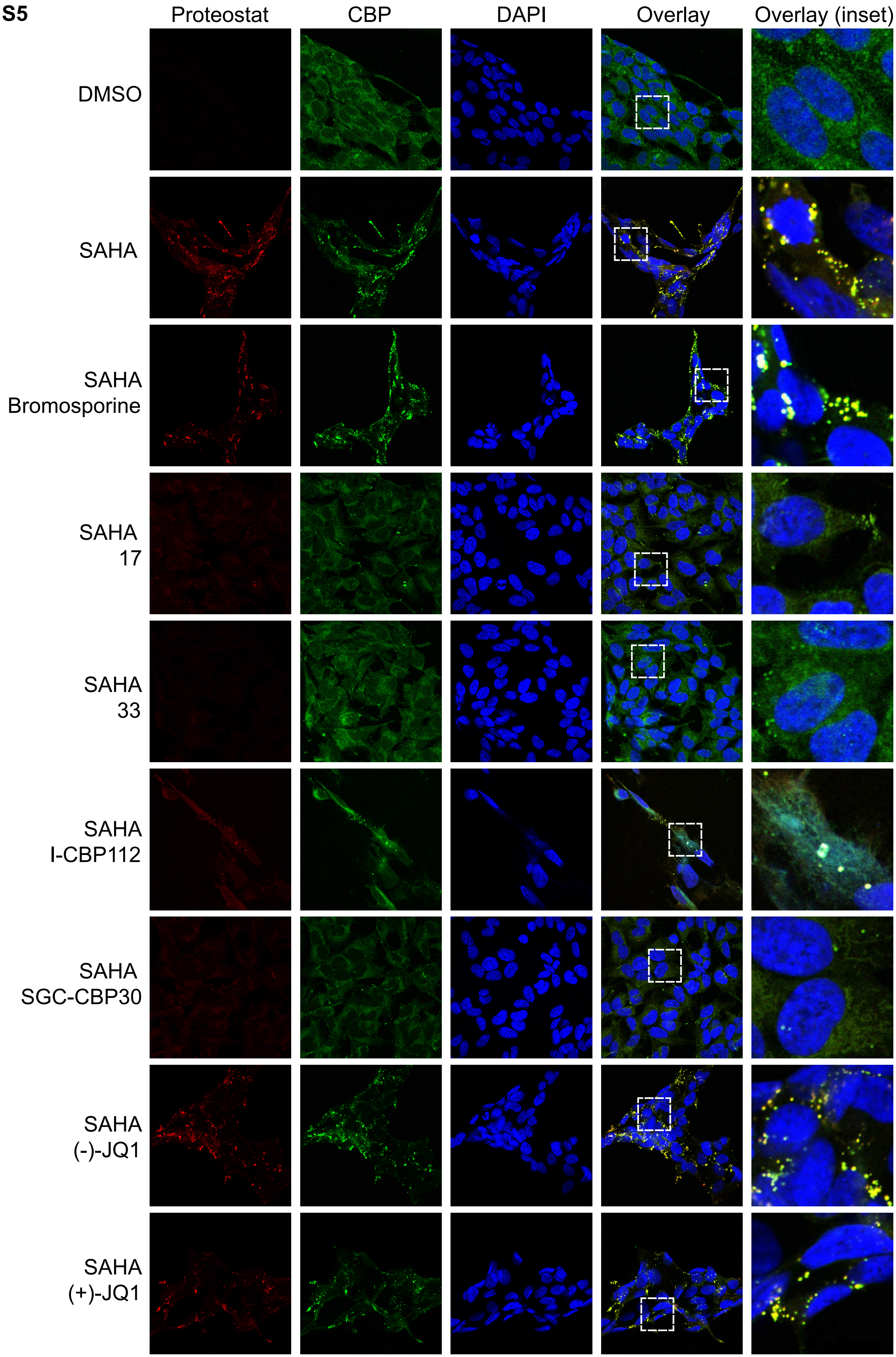
Proteostat
CBP
DAPI
Overlay
Overlay (inset)
DMSO
SAHA
SAHA Bromosporine
SAHA 17
SAHA 33
SAHA I-CBP112
SAHA SGC-CBP30
SAHA (-)-JQ1
SAHA (+)-JQ1

Figure S5 (related to Figure 4)

SAHA induced aggregates are reduced in neuroblastoma cells upon treatment with CBP/p300 specific BDIs. SH-SY5Y cells were treated with SAHA (5 μM) or DMSO (-) and in parallel with 2.5 μM of the indicated BDIs. Cells were fixed, permeabilized and stained with an antibody against endogenous CBP (green), Proteostat (red) and DAPI (blue) was used as a nuclear counterstain. Panels on the right represent the enlarged area shown by a dashed square on the left side, and aggregates which co-localize with endogenous CBP appear as yellow in the images.

Fig. S6

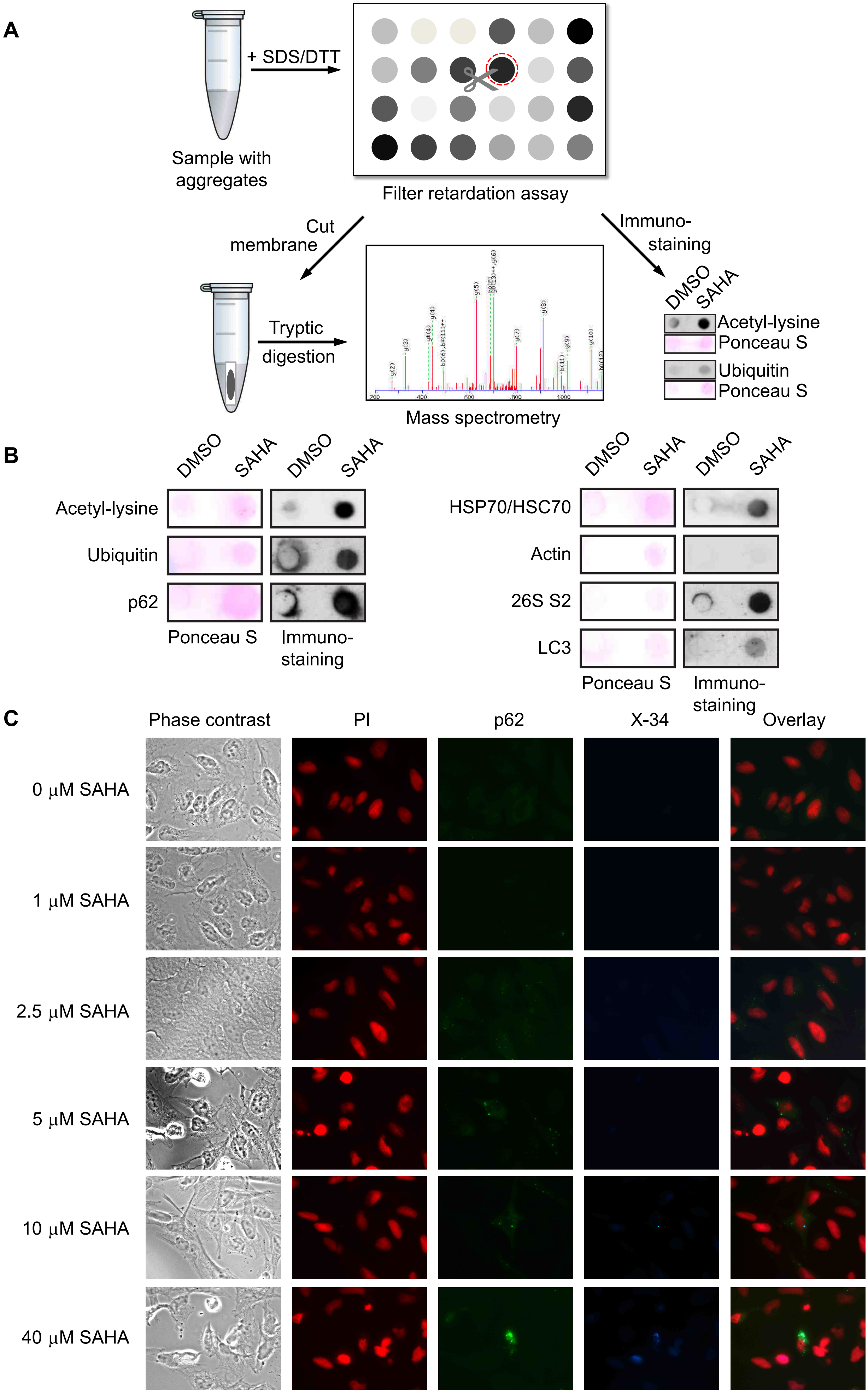
A
+ SDS/DTT
Sample with aggregates
Filter retardation assay
Cut membrane
Immuno-staining
Tryptic digestion
Mass spectrometry
DMSO
SAHA
Acetyl-lysine
Ponceau S
Ubiquitin
Ponceau S
B
DMSO
SAHA
DMSO
SAHA
Acetyl-lysine
Ubiquitin
p62
Ponceau S
Immuno-staining
HSP70/HSC70
Actin
26S S2
LC3
Ponceau S
Immuno-staining
C
Phase contrast
PI
p62
X-34
Overlay
0 μM SAHA
1 μM SAHA
2.5 μM SAHA
5 μM SAHA
10 μM SAHA
40 μM SAHA

Figure S6 (related to Figure 6)

Scheme of the filter retardation assay to enrich aggregated proteins and co-localisation of p62 with X-34 positive aggregates.

(A) U2OS cells containing aggregated proteins were subjected to lysis buffer with detergent and DTT. Samples were filtered on a cellulose acetate membrane and aggregates with a size of more than 200 nm retained. The membrane was then washed and subjected to mass spectrometry in order to identify the retained proteins. In parallel, aggregates were analysed on the membrane by immunostaining, and the total protein amounts were visualized by Ponceau S staining.

(B) U2OS cells were either treated with DMSO or SAHA (5 µM) for 24 h, then harvested, membranes and DNA removed and the remaining aggregate fraction treated with detergent. The material captured in the filter retardation assay was visualized by Ponceau S staining or immunostaining with the indicated antibodies.

(C) Co-localization of p62 with X-34 positive aggregates. p62 was detected upon treatment of U2OS cells with increasing concentrations of SAHA (green), in parallel cells were stained with the dye X-34 (blue), specifically recognizing amyloid-like aggregates. PI staining was employed to visualize the nuclei (red).

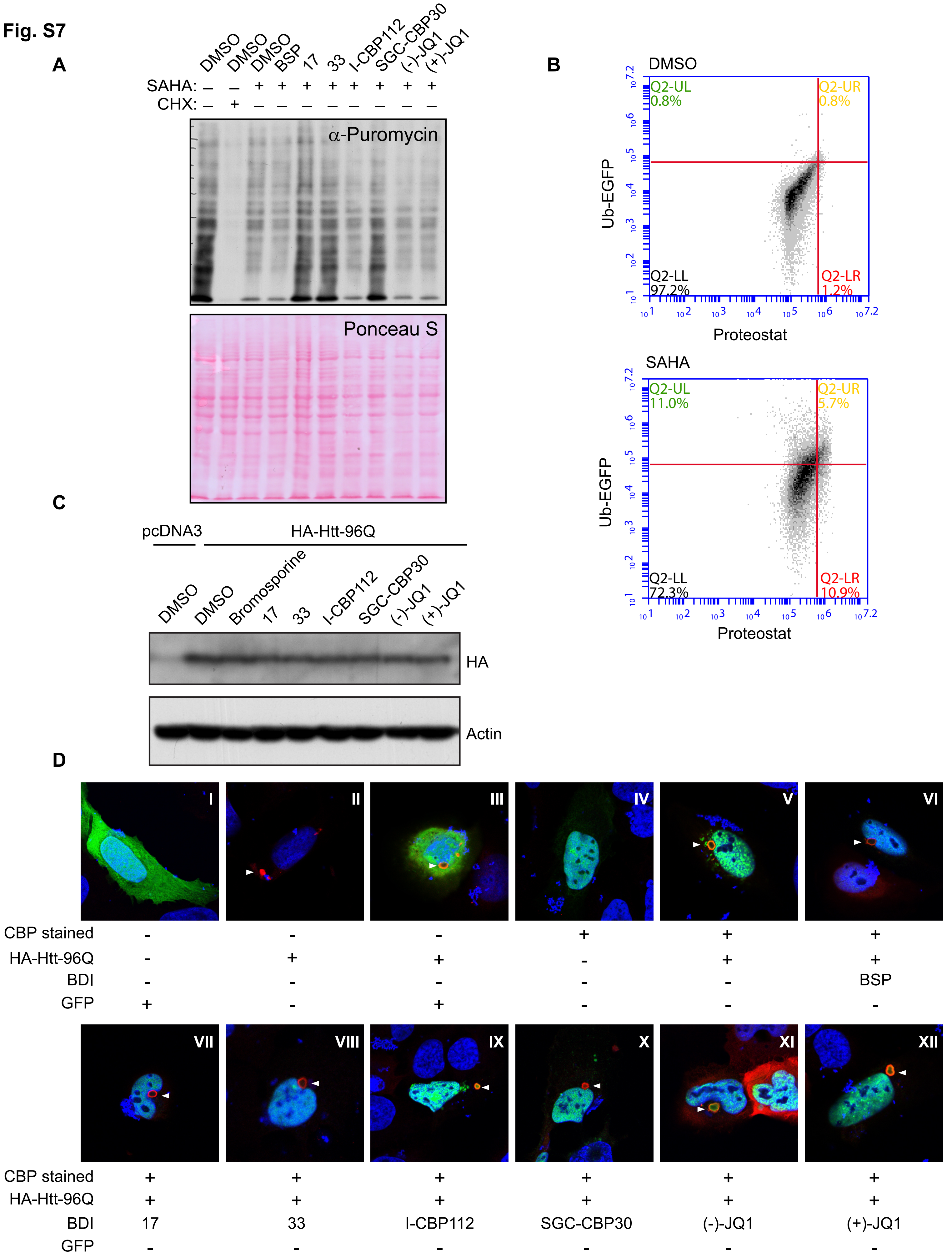
Fig. S7
A
DMSO DMSO DMSO BSP 17 33 I-CBP112 SGC-CBP30 (-)-JQ1 (+)-JQ1
SAHA: – – + + + + + + + +
CHX: – + – – – – – – – –
α-Puromycin
Ponceau S
B
DMSO
Q2-UL 0.8%
Q2-UR 0.8%
Q2-LL 97.2%
Q2-LR 1.2%
Ub-EGFP
Proteostat
SAHA
Q2-UL 11.0%
Q2-UR 5.7%
Q2-LL 72.3%
Q2-LR 10.9%
Ub-EGFP
Proteostat
C
pcDNA3
HA-Htt-96Q
DMSO DMSO Bromosporine 17 33 I-CBP112 SGC-CBP30 (-)-JQ1 (+)-JQ1
HA
Actin
D
I II III IV V VI
CBP stained - - - + + +
HA-Htt-96Q - + + - + +
BDI - - - - - BSP
GFP + - + - - -
VII VIII IX X XI XII
CBP stained + + + + + +
HA-Htt-96Q + + + + + +
BDI 17 33 I-CBP112 SGC-CBP30 (-)-JQ1 (+)-JQ1
GFP - - - - - -

Figure S7 (related to Figure 6 and Figure 7)
Effects of BDIs on protein biosynthesis and degradation and on the pathologically elongated form of huntingtin.

(A) Cells were treated with SAHA (5 μM) and the indicated BDIs and then the effect on mRNA translation was visualized by incorporation of puromycin in the nascent polypeptide chain and subsequent immunoblotting with a monoclonal antibody against puromycin in polypeptide chains. For comparison, Ponceau S serves as a loading control. A representative anti-puromycin immunoblot is shown and the corresponding Ponceau S staining.

(B) FACS analysis of HEK293T cells stably expressing Ub-EGFP. Green fluorescence (FL1 channel) of the Ub-EGFP is plotted on the y-axis, red fluorescence of Proteostat staining (FL3 channel) on the x-axis. Quadrants were chosen according to the DMSO control which is set to the lower left quadrant. Upper right quadrants are used in Figures 6D and 6E.

(C) U2OS cell expressing the HA-Htt-96Q exon 1 were treated with the different BDIs. Immunoblotting shows that the level of Htt is not altered. β-actin serves as loading control.

(D) U2OS cells overexpressing HA-Htt-96Q exon1 and CBP-GFP or GFP, with or without different BDIs. Cell were incubated with an HA-antibody against HA-Htt-96Q (shown in red), whereas CBP-GFP or GFP is shown in green and nuclei were counterstained with DAPI (blue). Overlaid channels are displayed, where co-localization appears in yellow or orange. In (I), cells were transfected with GFP only, in (II) with HA-Htt-96Q only, in (III) with both constructs. Cells were transfected with CBP-GFP in (IV) and with both HA-Htt-96Q and CBP-GFP in (V), co-localized Htt-96Q and CBP-GFP (orange) are indicated by an arrow. In (I) to (V), cells were treated with DMSO, whereas ascending from (VI) to (XII), cells were treated with the different BDIs in the following order: Bromosporine (BSP), 17, 33, I-CBP112, SGC-CBP30, (-)-JQ1 and (+)-JQ1. Loss of co-localization between HA-Htt-96Q and CBP-GFP in the panels VII (BDI 17), VIII (BDI 33) and X (BDI SGC-CBP30) is shown in red and indicated by an arrow.

Table S1, related to Experimental Procedures, Bromodomain Inhibitors

Overview of the bromodomain inhibitors (BDIs) used in this study. Listed are names, chemical structures, specificities and respective references.

BDI	Structure	Specificity
Bromosporine	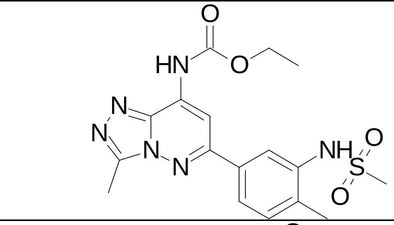	Bromosporine - inhibitor of several bromodomain proteins
17	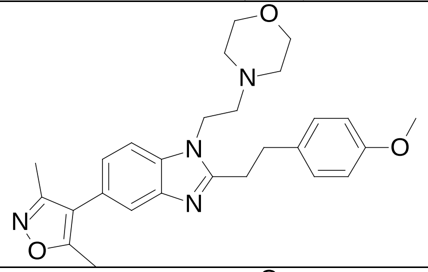	Potent and selective inhibitor of CBP/p300 bromodomain.<br>Kd < 100 nM<br>(Hay, et al., 2014)
33	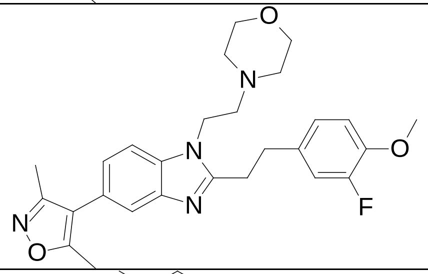	Analogue of 17, more potent, Kd = 50 nM and more selective over BRD4 (1μM)<br>(Hay, et al., 2014)
I-CBP112	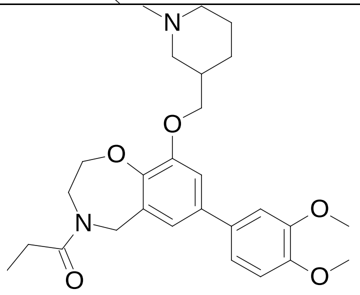	Selective bromodomain inhibitor with dissociation constant<br>Kd = 151 ± 6 nM and 167 ± 8 nM for CBP and p300, respectively<br>(Picaud, et al., 2015)
SGC-CBP30	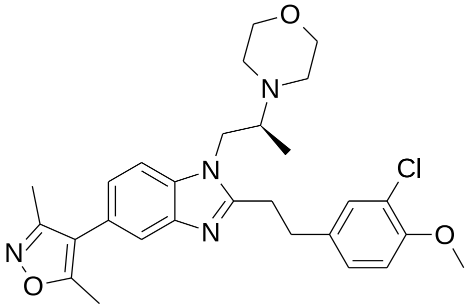	Chemical probe for CPB/p300,<br>Kd = 21/30 nM, 40 fold selectivity over BRD4<br>(Hay, et al., 2014)

Table S1 (continued), related to Experimental Procedures, Bromodomain Inhibitors

Overview of the bromodomain inhibitors (BDIs) used in this study. Listed are names, chemical structures, specificities and respective references.

BDI	Structure	Specificity
(-)-JQ1	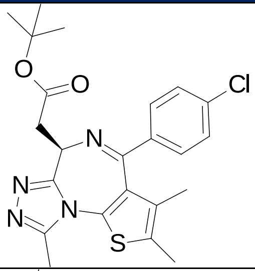	Negative control for (+)-JQ (Filippakopoulos, et al., 2010)
(+)-JQ1	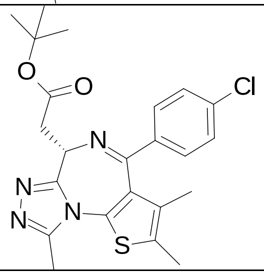	Chemical probe for BET bromodomains,<br>Kd < 100 nM<br>(Filippakopoulos, et al., 2010)

Filippakopoulos, P., Qi, J., Picaud, S., Shen, Y., Smith, W.B., Fedorov, O., Morse, E.M., Keates, T., Hickman, T.T., Felletar, I., et al. (2010). Selective inhibition of BET bromodomains. Nature 468, 1067-1073.

Hay, D.A., Fedorov, O., Martin, S., Singleton, D.C., Tallant, C., Wells, C., Picaud, S., Philpott, M., Monteiro, O.P., Rogers, C.M., et al. (2014). Discovery and optimization of small-molecule ligands for the CBP/p300 bromodomains. Journal of the American Chemical Society 136, 9308-9319.

Picaud, S., Fedorov, O., Thanasopoulou, A., Leonards, K., Jones, K., Meier, J., Olzscha, H., Monteiro, O., Martin, S., Philpott, M., et al. (2015). Generation of a Selective Small Molecule Inhibitor of the CBP/p300 Bromodomain for Leukemia Therapy. Cancer Res 75, 5106-5119.

Table S2, related to Figure 6

Proteins detected in mass spectrometry upon filter retardation. Gene symbols, potential acetylation and percentages of lysine and arginine residues are displayed as well as molecular weight and PI.

Accession no.	Gene Symbol	PI	Mass	Acetylation databases Phosida, UniProt, MaxQB, CPLM and PhosphoSitePlus)	Lysine residues in %	Arginine residues in %
A6NHL2	TUBAL3	5.68	50675	Yes	3.6	6.1
A6NJY1	SLC9B1P1	7.66	31093	No	5	2.1
A6NMY6	ANXA2P2	6.49	38806	Yes	9.4	5.9
A6NNZ2	TBB8L	4.75	50168	No	2.7	5
O00567	NOP56	9.24	66408	Yes	10.9	5.2
O14979	HNRPDL	9.59	46580	Yes	6.7	7.9
O15049	N4BP3	8.33	60889	No	4.4	10.3
O43175	PHGDH	6.29	57356	Yes	4.9	4.1
O60282	KIF5C	5.86	109997	Yes	9.9	5.7
O94925	GLS	7.85	74269	Yes	6.1	4.9
O95573	ACSL3	8.65	81338	Yes	8.2	3.8
P00387	CYB5R3	7.18	34441	Yes	5.3	6
P04181	OAT	6.57	48846	Yes	6.4	4.8
P04350	TUBB4A	4.78	50010	Yes	3.2	5
P04792	HSPB1	5.98	22826	Yes	3.4	7.8
P05198	EIF2S1	5.02	36374	Yes	6.7	7.9
P07814	EPRS	7.02	172080	Yes	9.8	3.9
P07900	HSP90AA1	4.94	85006	Yes	10.9	4.1
P07902	GALT	6.49	43621	No	3.2	6.9
P08107	HSPA1A	5.48	70294	Yes	7.8	4.8
P08134	RHOC	6.2	22334	No	7.8	7.8
P08559	PDHA1	8.35	43952	Yes	5.9	7.2
P08865	RPSA	4.79	32947	Yes	3.7	5.1
P09382	LGALS1	5.34	15048	Yes	5.9	3.7
P0CG38	POTEI	5.83	122858	Yes	8	4.8
P11142	HSPA8	5.37	71082	Yes	8.4	4.3
P11177	PDHB	6.2	39550	Yes	5.3	5
P11586	MTHFD1	6.89	102180	Yes	6.6	4.6
P13639	EEF2	6.41	96246	Yes	7.6	5.1
P13804	ETFA	8.62	35400	Yes	7.8	3.3
P14618	PKM	7.96	58470	Yes	7	5.8
P14625	HSP90B1	4.76	92696	Yes	9.7	4.7
P15880	RPS2	10.25	31590	Yes	8.2	8.2
P17066	HSPA6	5.81	71440	Yes	6.1	7
P18621	RPL17	10.18	21611	Yes	13.6	8.2
P21333	FLNA	5.7	283301	Yes	6	3.5
P21817	RYR1	5.18	570517	Yes	4.5	5.8
P22087	FBL	10.18	33877	Yes	5.9	9

Table S2 (continued), related to Figure 6

Proteins detected in mass spectrometry upon filter retardation. Gene symbols, potential acetylation and percentages of lysine and arginine residues are displayed as well as molecular weight and PI.

Accession no.	Gene Symbol	PI	Mass	Acetylation databases Phosida, UniProt, MaxQB, CPLM and PhosphoSitePlus)	Lysine residues in %	Arginine residues in %
P22492	HIST1H1T	11.71	22006	Yes	19.3	6.8
P23246	SFPQ	9.45	76216	Yes	4.7	8.5
P23396	RPS3	9.68	26842	Yes	8.2	7.4
P23526	AHCY	5.92	48255	Yes	6.7	3.9
P25705	ATP5A1	9.16	59828	Yes	5.4	6.9
P31415	CASQ1	4.03	45132	Yes	6.1	2.3
P31943	HNRNPH1	5.89	49484	Yes	3.6	6.9
P32119	PRDX2	5.66	22049	Yes	7.1	5.1
P36578	RPL4	11.07	47953	Yes	13.3	9.6
P40939	HADHA	9.16	83688	Yes	9.3	4.3
P42704	LRPPRC	5.81	159003	Yes	7.3	4.8
P46776	RPL27A	11	16665	Yes	12.8	8.8
P46777	RPL5	9.73	34569	Yes	11.8	8.1
P49327	FASN	6.01	275877	Yes	3.4	5.7
P49411	TUFM	7.26	49852	Yes	6.6	6
P49736	MCM2	5.34	102516	Yes	4.8	7.6
P50914	RPL14	10.94	23531	Yes	17.2	6
P50990	CCT8	5.42	60153	Yes	7.5	3.3
P52701	MSH6	6.5	154514	Yes	7.4	6.3
P54136	RARS	6.26	76129	Yes	8.3	5.3
P57721	PCBP3	8.22	39725	Yes	5.1	3.8
P61160	ACTR2	6.3	45017	Yes	6.3	6.3
P61978	HNRNPK	5.39	51230	Yes	4.8	7.8
P62241	RPS8	10.32	24475	Yes	14.9	11.1
P62244	RPS15A	10.14	14944	Yes	8.5	8.5
P62249	RPS16	10.21	16549	Yes	11	9.6
P62701	RPS4X	10.16	29807	Yes	10.3	8.4
P62753	RPS6	10.85	28834	Yes	14.5	12.9
P62826	RAN	7.01	24579	Yes	8.3	4.2
P62829	RPL23	10.51	14970	Yes	10.7	7.9
P62913	RPL11	9.64	20468	Yes	9	9.6
P62937	PPIA	7.68	18229	Yes	8.5	3.6
P63244	GNB2L1	7.6	35511	Yes	5.4	4.4
P68363	TUBA1B	4.94	50804	Yes	4.2	4.9
P68371	TUBB4B	4.79	50255	Yes	3.4	5.2
P78527	PRKDC	6.75	473749	Yes	6.7	5.5
Q00839	HNRNPU	5.76	91269	Yes	8.5	11.9

Table S2 (continued), related to Figure 6

Proteins detected in mass spectrometry upon filter retardation. Gene symbols, potential acetylation and percentages of lysine and arginine residues are displayed as well as molecular weight and PI.

Accession no.	Gene Symbol	PI	Mass	Acetylation databases Phosida, UniProt, MaxQB, CPLM and PhosphoSitePlus)	Lysine residues in %	Arginine residues in %
Q02543	RPL18A	10.73	21034	Yes	9.7	3.9
Q05639	EEF1A2	9.11	50780	Yes	9.9	3.9
Q06210	GFPT1	6.66	79555	Yes	6.4	6.3
Q06830	PRDX1	8.27	22324	Yes	9.5	3
Q08211	DHX9	6.41	142181	Yes	5.1	5.8
Q12931	TRAP1	8.3	80345	Yes	6.3	7.5
Q13268	DHRS2	9.21	27764	Yes	4.6	6.1
Q13439	GOLGA4	5.33	261892	No	12.7	4
Q13509	TUBB3	4.83	50856	Yes	3.6	4.9
Q13885	TUBB2A	4.78	50274	Yes	3.4	4.9
Q14103	HNRNPD	7.62	38581	Yes	9	3.9
Q14204	DYNC1H 1	6.01	534809	Yes	6.3	6.2
Q14315	FLNC	5.65	293407	Yes	5.3	4.3
Q14568	HSP90AA 2	4.57	39454	Yes	10.8	2
Q15149	PLEC	5.74	533462	Yes	5.3	9.7
Q15233	NONO	9.01	54311	Yes	5.7	9.8
Q15365	PCBP1	6.66	37987	Yes	3.9	4.5
Q16695	HIST3H3	11.13	15613	Yes	9.6	13.2
Q3ZCM7	TUBB8	4.79	50257	No	3.2	5.2
Q562R1	ACTBL2	5.39	42318	Yes	4.8	5.3
Q58FF6	HSP90AB 4P	4.65	58855	No	10.1	2.4
Q58FF7	HSP90AB 3P	4.71	68624	No	9.5	3
Q58FF8	HSP90AB 2P	4.79	44492	Yes	12.3	2.6
Q58FG1	HSP90AA 4P	5.07	47796	No	9.1	4.3
Q5JNZ5	RPS26P1 1	10.55	13336	No	10.4	12.2
Q5VTE0	EEF1A1P 5	9.15	50495	Yes	10.4	3.7
Q5VX52	SPATA1	8.54	50504	No	10.5	5
Q6NVV1	RPL13AP 3	10.76	12184	No	20.6	9.8
Q70CQ4	USP31	9.35	148328	No	5.8	6.7
Q71DI3	HIST2H3 A	11.27	15436	Yes	9.6	13.2

Table S2 (continued), related to Figure 6

Proteins detected in mass spectrometry upon filter retardation. Gene symbols, potential acetylation and percentages of lysine and arginine residues are displayed as well as molecular weight and PI.

Accession no.	Gene Symbol	PI	Mass	Acetylation databases Phosida, UniProt, MaxQB, CPLM and PhosphoSitePlus)	Lysine residues in %	Arginine residues in %
Q71DI3	HIST2H3A	11.27	15436	Yes	9.6	13.2
Q7KZ85	SUPT6H	4.81	200203	Yes	6.8	6.3
Q8IYB3	SRRM1	11.84	102331	Yes	11.3	15.9
Q8N257	HIST3H2BB	10.31	13900	Yes	15.9	6.3
Q8WZ42	TTN	6.02	3842904	Yes	8.6	4.8
Q92538	GBF1	5.48	208367	Yes	5	5
Q92841	DDX17	8.53	80906	Yes	4.3	8.1
Q96AE4	FUBP1	7.18	67690	Yes	4.3	4.5
Q96KN1	FAM84B	5.34	34681	No	3.5	7.1
Q99714	HSD17B10	7.66	27134	Yes	4.2	4.2
Q9BUF5	TUBB6	4.77	50281	Yes	3.1	4.9
Q9BYX7	POTEKP	5.91	42331	No	5.9	4.5
Q9NQC3	RTN4	4.43	130250	Yes	7	2.1
Q9NR30	DDX21	9.32	87804	Yes	11.6	5.5
Q9NY65	TUBA8	4.94	50746	Yes	4.2	4.7
Q9UQ35	SRRM2	12.05	300179	Yes	4.7	17
Q9Y3R0	GRIP1	6.03	123202	Yes	5.7	5.2
**Mean**					**7.5**	**6.1**

Supplemental experimental procedures

Antibodies

Anti-Ub (P4D1) (Santa Cruz, sc-8017), anti-acetyl-histone H3 (Millipore, 06-599), anti-LC3B (Cell Signaling, 2775), anti-p53 (Santa Cruz, sc-126), anti-acetylated tubulin (Sigma, T7451), anti-proteasome 26S S2 (Abcam, ab197054), anti-p62/SQSTM1 (Santa Cruz, sc-28359), anti-HSP90α/β (F-8) (Santa Cruz, sc-13119), anti-acetylated-lysine (Cell Signaling, 9441), anti-HSP70/HSC70 (W27) (Santa Cruz, sc-24), anti-α-tubulin (Santa Cruz, sc-8035), anti-p300 (C-20) (Santa Cruz, sc-585), anti-CBP (C-1) (Santa Cruz, sc-7300), anti-GFP (Roche, 11814460001), anti-puromycin (clone 12D10) (Millipore, MABE343), anti-HA.11 (Clone 16B12) (Covance, MMS-101P), anti-Flag (Sigma, F3165), anti-mouse-Alexa Fluor®488 (Invitrogen, R37114), anti-rabbit-Alexa Fluor®488 (Invitrogen, A-21206), anti-mouse-Alexa Fluor®594 (Invitrogen, A-21203), anti-rabbit-Alexa® Fluor 594 (Invitrogen, A-21207), anti-mouse-HRP (Millipore, 12-349), anti-rabbit-HRP (Millipore, 12-348).

Cell viability and measurement of apoptosis

The overall cell viability was determined by means of MTT assay. Cells treated with inhibitors and/or transfected in a 96-well plate in 100 µl medium were incubated with MTT (3-(4,5-dimethylthiazol-2-yl)-2,5-diphenyltetrazolium bromide) tetrazolium solution (Sigma, M2128) with a final concentration of 0.5 mg/ml. Cells were incubated for exactly 120 min, and the reaction of the conversion of the MTT to purple formazan crystals was stopped by adding 100 µl of solubilizing solution (20 % SDS, 50 % dimethylformamide (DMF)). Cells and crystals were solubilized in this solution overnight and the quantity of formazan directly proportional to the number of viable cells was measured by recording changes in absorbance at 570 nm using a plate reading spectrophotometer (Tecan), as modified by (Mosmann, 1983). SubG1 phase obtained after cell cycle analysis in flow cytometry (see flow cytometry) correlated with the number of dying cells, whereby it could not be distinguished between apoptosis, necrosis or other types of cell death. To differentiate between the different events, cleaved caspase-3 or cleaved PARP served as apoptotic markers (see also section immunoblotting).

RNA interference

U2OS cells were transfected with CBP esiRNA or p300 esiRNA (Sigma, EHU155151-20UG and EHU075681-20UG) or control GFP siRNA, mixed in OptiMEM and Oligofectamine (Invitrogen, 12252011) as described in the manufacturer's instructions. The mixture was added to 1 x 10^5 cells in 1 ml medium (2 ml total) for 6 h, seeded into a 6-well plate and incubated for 4 days. 24 h before the lysis for immunoblotting or fixation for microscopy or FACS, cells were treated with SAHA (5 µM) and the respective BDIs (2.5 µM).

Cell lysis and immunoblotting

Cells were split and 1 day later they were seeded in 10 cm dishes, for each sample 1.5 million of cells. The cells were then treated 24 h later with the respective compounds and/or were transfected or the protein was induced by doxycycline treatment. The cells were harvested and washed twice with ice-cold PBS and lysed with ice-cold modified RIPA buffer (50 mM Tris pH 7.4, 150 mM NaCI, 1 mM EDTA, 0.25 % sodium deoxycholate, 1.0 % NP-40, protease inhibitors 10 µg/ml Aprotinin (Sigma), 10 µg/ml Leupeptin (Sigma), 10 µg/ml Pepstatin (Roche), 50 µg/ml AEBSF (Sigma), 1 µM MG132 (Calbiochem), 10 µM SAHA, 20 µM NEM (Sigma)) at 4ºC for 20 min. Protein concentration was determined according to Bradford. 40 µg of cell lysate was diluted in 2x Laemmli buffer and an SDS-PAGE was performed, followed by immunoblot. Equal loading was monitored *via* Ponceau S staining. The membranes were blocked for 1 h in 5 % dry milk in TBS and then decorated with the primary antibodies over-night. On the next day, the membranes were washed 3 x with TBS and then decorated with a secondary antibody conjugated to HRP for 2 h. After washing 2 x with TBS-T and once with TBS, the membranes were incubated with ECL and the signal detected with films.

Filter retardation assay

In order to isolate aggregated species of different unknown proteins under native conditions, at least 24 million differently treated (DMSO and SAHA) cells were lysed. They were washed twice in PBS and were lysed in 1400 µl of a mild lysis buffer containing 0.1 % NP40, 100 mM NaCl, 20 mM Tris, 10 mM $MgCl_2$, pH 7.4, protease inhibitors (10 µg/ml Aprotinin (Sigma), 10 µg/ml Leupeptin (Sigma), 10 µg/ml Pepstatin (Roche), 50 µg/ml AEBSF (Sigma), 1 µM MG132 (Calbiochem, 474790)), 10 µM SAHA, 20 µM NEM (Sigma, E3876) and 80 units benzonase® nuclease (Millipore, 70746) for 1 h. The lysate was centrifuged with only 2000 g for 5 min, in order to remove the cell debris, but to keep the aggregates soluble (Scherzinger, et al., 1997). The supernatant with the

aggregates was kept on ice and the protein concentration was determined according to Bradford. Detergent was titrated to optimize the filter retardation process, but not to destroy the aggregates, and for this reason the final concentration was brought to 1.3 % SDS and 33 mM DTT. Similar protein amounts were then filtered through a cellulose acetate membrane with a pore size of 0.2 μm (Sterlitech) in a dot blot apparatus. Membranes were washed 3 times with 400 μl PBS and air dried. They were then stained with Ponceau S to visualize the retained protein amount. The membranes were then either treated as described in immunoblotting to detect individual proteins amongst the retained proteins with antibodies, or 10 dots from the membrane with the retained proteins on it were cut and pooled and further processed to analyse the proteins *via* mass spectrometry.

Preparation for mass spectrometry (MS) analysis

The digestion of peptides from a membrane was modified after (Fernandez and Mische, 2001), since cellulose acetate was used in this case. Briefly, membrane dots with retained proteins after performing the filter retardation assay were cut, 10 of them were air dried, cut into 1 mm large pieces, pooled and put in a 1.5 ml pre-lubricated microcentrifuge tube (Fisher Scientific). Usually, empty membranes were run as negative control to determine any protein adherent to the empty membrane and exclude them from the results. 100 μl digestion buffer (50 mM NH_4HCO_3 in ddH_2O, pH 8.0) was added and the samples were sonicated for 5 min and vortexed for a minute. The samples were then incubated at room temperature for 30 min with rigorous shaking at 1400 rpm, and then again sonicated for 5 min. and vortexed for a minute. 5 μl DTT was then added to a final concentration of 10 mM, and samples were incubated at room temperature for 1 h. Then 20 μl chloracetamide was added to a final concentration of 55 mM and incubated for 1 h. 20 μl DTT was added to a final concentration of 10 mM and the samples were again incubated for 1 h at room temperature with shaking. The samples were then filled up with 775 μl digestion buffer, and 25 μl of a 0.2 mg/ml stock solution of trypsin (Promega, V5111) in digestion solution was added, and the samples were digested in solution overnight at 37 ºC. They were then for 5 min sonicated, and vortexed for 1 min. This was repeated twice, the samples were then spun at 16000 g and the supernatant given in a new pre-lubricated tube. To the remaining cellulose acetate fragments, 100 μl digestion buffer was added, and the samples were sonicated for 5 min. and vortexed for 1 min., this was repeated once, then spun and the supernatant combined with the previous one. Peptides were then purified by the Sep-Pak C18 light (Waters) purification columns according to the manufacturer's recommendation. Samples were washed with washing solution (98 % MilliQ-H_2O, 2 % CH_3CN, 0.1 % formic acid), and eluted with elution solution (35 % MilliQ-H_2O, 65 % CH_3CN, 0.1 % formic acid).

Analysis of MS data

Analysis of the proteins was carried out as described previously (New, et al., 2013). In brief, samples were subjected to nLC-MS/MS analysis, and peptides and proteins identified by Mascot (v2.3.01 CBRG Cluster) *via* automated database searching of all MS/MS spectra against the UniProt SwissProt swissprot/uniprot_sprot.v2012.07.13 homo sapiens database (20,306 sequences). The data were analysed with the following search parameters: Type of search: MS/MS Ion Search; Enzyme: Trypsin; Fixed modifications: Carbamidomethyl (C); Variable modifications: Oxidation (M), Deamidated (NQ), Acetyl (K), GlyGly (K); Mass values: Monoisotopic; Protein mass: Unrestricted; Peptide mass tolerance: ± 1.8 Da (# 13C = 1); Fragment mass tolerance: ± 0.5 Da; Max missed cleavages: 3; Instrument type: ESI-TRAP. After removing known contaminants from the list of detected proteins, only those proteins, for which at least two significant peptide matches were identified, were accepted. The UniProt accession numbers were searched against the STRING database version 9.0 (Jensen, et al., 2009) for protein-protein interactions. Only interactions between the proteins belonging to the dataset were selected. STRING defines a metric called confidence score to define interaction confidence; all interactions for the dataset were chosen which had a confidence score ≥ 0.4. The clustering was performed *via* the MCL option which accepts a parameter called "inflation" and was set to 2. Further analysis and the determination of GO enrichments were carried out with Cytoscape 2.8.3 (Shannon, et al., 2003) and plugin BiNGO 2.44 (Maere, et al., 2005). The following parameters were applied for the BiNGO search: Hypergeometric test; Benjamini and Hochberg false discovery rate correction; significance level: 0.05; GO biological process; organism: Homo sapiens. The following databases were searched against acetylation (K) and acetylation sites: MaxQB, UniProt, Phosida, Compendium of Protein Lysine Modifications (CPLM) and PhosphoSitePlus.

Analysis of protein synthesis by puromycin labelling

In order to measure translation by non-radioactive means, puromycin labelling during protein synthesis was applied. The experiments were modified after (Schmidt, et al., 2009). Cells were grown in 10 cm dishes, treated

accordingly to the experiment, trypsinized and harvested at 37 ºC, and the cell number were adjusted to 1 million in a microtube with 1 ml pre-warmed medium. In control samples, cycloheximide was brought to a final concentration of 100 μM and cells were incubated at 37 ºC for 30 min, and the tubes were gently mixed every 10 min. Puromycin was added to a final concentration of 10 μg/ml and the cells were incubated for further 10 min at 37 ºC with gently shaking every 5 min. The cells were then spun for 2 min at 500 g in a pre-warmed (37 ºC) centrifuge and once quickly washed with pre-warmed PBS. They were then immediately frozen to – 80 ºC, and after 15 min they were lysed as described in RIPA buffer. With the lysates, immunoblotting was performed, and an anti-puromycin antibody (clone 12D10, Millipore) was used to detect puromycinylated protein species. The signal was quantitated with ImageJ and the Fiji plugin (Schneider, et al., 2012) and normalized to the total protein amount, visualized by ponceau S staining.

Fluorescence imaging

Fluorescence imaging of the adherent or semi-adherent cells was performed using an Olympus BX60 inverted microscope or a Zeiss LSM 780 confocal laser scanning microscope equipped with a with the 32 channel GaAsP detector. Images were acquired using the Leica HCX PL APO 63X oil immersion objective with numerical aperture of 1.4 and a resolution of 1024 x 1024. Virtual zoom for most confocal image was set to 1.00 or 2.5 and scales are indicated by a white bar and image acquisition and analysis was performed using Leica TCS Analysis software. Images were recorded in czi image format, processed with the blue ZEN Zeiss software and converted into Tiff files. X-34, DAPI and Thioflavin S stained samples were recorded in the blue fluorescent channel or, if the confocal microscope was used, with an excitation wavelength of a diode laser (405 nm, CW/pulsed) 30 mW, Proteostat stained samples were excitated with red fluorescent light or a HeNe-laser (543 nm) 1 mW, and structures stained with an antibody coupled to Alexa 594 were excitated with red fluorescent light or a HeNe-laser (594 nm) 2 mW. GFP or proteins which were detected by an Alexa 488 labeled antibody were excited with an Ar-laser (488 nm) 35 mW. The filters were set to the respective emission wavelength of the different dyes, which is 500 nm for X-34 and Thioflavin S determined by a λ-scan in the Zeiss LSM 780 microscope, and 620 nm for Proteostat.

Fluorescence Recovery After Photobleaching (FRAP)

U2OS cells were grown in 8-well μ-slide chambers (ibidi) and were treated with the respective pan-HDAC inhibitor and different BDIs, or transfected with HA-Htt-96Q. After two days in case of the pan-HDAC treatment or one day in case of Htt transfection, cells were stained with the dye X-34 for 45 minutes, which can penetrate through cell membranes and selectively stain amyloid-like aggregates. Cells were then washed for 15 minutes with pre-warmed medium. The FRAP and imaging system consisted of a Zeiss LSM 780 AxioObserver microscope equipped with a high-numerical-aperture PlanApochromat 63x/1.40 Oil DIC M27 oil immersion objective (Zeiss). Cells in the chambers were placed in an incubator capable of maintaining temperature (37 ºC), humidity and CO_2 (5 %) atmosphere. FRAP imaging was carried out with a 405 nm laser and with a PMT detector set to detect fluorescence between 416 nm and 585 nm. A region of interest within the aggregate was empirically selected for bleaching. A time lapse series was taken to record X-34 fluorescence recovery using 0.5 % of the power used for bleaching. Initial scanning prior to bleaching was carried out to record the baseline for correcting the bleaching caused by the detection. 150 frames were recorded; with an image size of 33.74 μm. Zoom was 4.0 and the frame time was 0.97 s. The data were fitted by the ZEN 2012 microscope control software according to a one parameter exponential model to determine the average half-time for recovery for 5-10 cells per treatment in at least 5 independent experiments.

Analysis of aggregates with the IN Cell analyser

To quantify microscopic changes of aggregate number and size, 1500 to 5000 cells were seeded on a 96-well plate and treated accordingly. Cells were fixed, permeabilized and stained as described for immunofluorescence staining, with the difference that one staining was always performed with 5 μg/ml FITC (Sigma, F7250) or 1 μg/ml propidium iodide without prior RNAse A digestion. Nuclear staining was either performed with DAPI or propidium iodide with prior RNAse A treatment. Aggregates were stained either with X-34 or Proteostat as described in the section immunofluorescence staining. Cells were fixed again after all staining procedures for 30 min with 4% paraformaldehyde and then covered with 200 μl PBS. Intracellular structures were measured with an IN Cell analyser 1000 instrument (GE Healthcare) according to the manufacturer's instructions. Briefly, cells were analysed with 3 different wavelengths and filters in order to record blue, green and red fluorescence with a 20 x objective. 12 fields in each well were acquired in the centre of the well, so that in total per experiment and condition at least 1400 cells were analysed. Exposure times for X-34 were 1000 ms, for Proteostat 2000 ms, for the FITC counterstain and DAPI/PI staining 500 ms.

The raw images were then automatically analysed by the Multi Target Analysis Module for the IN Cell Analyzer 1000 (GE Healthcare), with the following parameters: Wave 1: Object nuclei, top hat, minimum area 200 μm^2, sensitivity 100; Wave 2: Object cells, multiscale top hat, minimum area 500 μm^2, sensitivity 25; Wave 3: Object organelles, multiscale top hat, granule size minimum 0.5 µm, granule size maximum 2 µm for HDAC inhibitor induced aggregates, 10 µm for Htt aggregates, process parameters 3 pixels, sensitivity 80, detect inclusions in cells; Reference in wave3: pseudo.

Flow cytometry

Aggregation analysis

Cells were harvested 24 h or 48 h after different treatments. Cells were washed once with pre-warmed PBS, trypsinized (Lonza), centrifuged at 400 g for 3 min. and washed again with PBS. They were fixed for 30 min. with 4 % paraformaldehyde in PBS, washed once in PBS and permeabilized for 30 min at 4 ºC in 0.5 % Triton. Cells were then washed 2 times in PBS and stained for 30 min with Proteostat in a 1: 7000 dilution in the provided buffer (EnzoLifesciences). Without further washing, cells were then analysed in a BD Accuri C6 FACS machine. At least 20000 events were analysed per condition and final gating. Cells were analysed in the FL3 channel, after gating from the FSC/SSC plot in order to exclude cell debris. In case the reporter protein Ub-EGFP was analysed in parallel, an additional gate were set to ensure the analysis in the FL1 channel. Compensation were then set correcting the FL1 channel for 7 % for the FL3 channel, and to correct the FL3 channel, compensation for the FL1 channel was set to 10 %. Log fluorescence intensities were plotted against each other and quadrants were set in order to determine the different degrees of UPS inhibition and aggregation. The mean FL1 and FL3 intensities in total or according to the set quadrants were used. The raw cytometry data were analysed using CFlowPlus (BD Bioscience). Flow cytometry data of aggregation in FL3 channel were analysed by comparison of mean fluorescence *via* calculation of a term referred to as the Aggregation Propensity Factor (APF), as defined below. APF = 100 x $(MFI_{treated} – MRI_{control})/MFI_{treated})$, wherein $MFI_{treated}$ and $MFI_{control}$ are the mean fluorescence intensity values from control and treated samples. The UPS inhibition factor (UIF) was calculated accordingly.

Cell cycle analysis

After treatment, cells were harvested using trypsin, washed in phosphate buffered saline in PBS (Oxoid) and fixed in 70 % ethanol in PBS. For analysis, the samples were incubated for 30 min with 100 µg/ml RNAse A (Sigma, R6513) and 1 µg/ml propidium iodide (Sigma, P4864) at 4 ºC. Samples were run on a BD Accuri C6 Flow Cytometer (BD Bioscience) and the analysis was carried out using CFlowPlus (BD Biosciences) software. Virtual gain was set to the G1 peak of DMSO treated cells, and other treatments were set accordingly to this peak, in order to have an alignment for the set the different markers for the cell cycle phases.

Statistical Analysis

Student's t test was used to test for statistical significance of the differences between the different group parameters. p values of less than 0.05 were considered statistically significant and error bars represent standard deviation (SD).

Supplemental References

Fernandez, J., and Mische, S.M. (2001). Enzymatic digestion of proteins on PVDF membranes. Current protocols in protein science / editorial board, John E. Coligan ... [et al.] Chapter 11, Unit 11 12.

Jensen, L.J., Kuhn, M., Stark, M., Chaffron, S., Creevey, C., Muller, J., Doerks, T., Julien, P., Roth, A., Simonovic, M., et al. (2009). STRING 8--a global view on proteins and their functional interactions in 630 organisms. Nucleic acids research 37, D412-416.

Maere, S., Heymans, K., and Kuiper, M. (2005). BiNGO: a Cytoscape plugin to assess overrepresentation of gene ontology categories in biological networks. Bioinformatics 21, 3448-3449.

Mosmann, T. (1983). Rapid colorimetric assay for cellular growth and survival: application to proliferation and cytotoxicity assays. J Immunol Methods 65, 55-63.

New, M., Olzscha, H., Liu, G., Khan, O., Stimson, L., McGouran, J., Kerr, D., Coutts, A., Kessler, B., Middleton, M., et al. (2013). A regulatory circuit that involves HR23B and HDAC6 governs the biological response to HDAC inhibitors. Cell Death Differ 20, 1306-1316.

Scherzinger, E., Lurz, R., Turmaine, M., Mangiarini, L., Hollenbach, B., Hasenbank, R., Bates, G.P., Davies, S.W., Lehrach, H., and Wanker, E.E. (1997). Huntingtin-encoded polyglutamine expansions form amyloid-like protein aggregates in vitro and in vivo. Cell 90, 549-558.

Schmidt, E.K., Clavarino, G., Ceppi, M., and Pierre, P. (2009). SUnSET, a nonradioactive method to monitor protein synthesis. Nature methods 6, 275-277.

Schneider, C.A., Rasband, W.S., and Eliceiri, K.W. (2012). NIH Image to ImageJ: 25 years of image analysis. Nature methods 9, 671-675.

Shannon, P., Markiel, A., Ozier, O., Baliga, N.S., Wang, J.T., Ramage, D., Amin, N., Schwikowski, B., and Ideker, T. (2003). Cytoscape: a software environment for integrated models of biomolecular interaction networks. Genome research 13, 2498-2504.
